# Mincle-mediated translational regulation is required for strong nitric oxide production and inflammation resolution

**DOI:** 10.1038/ncomms11322

**Published:** 2016-04-18

**Authors:** Wook-Bin Lee, Ji-Seon Kang, Won Young Choi, Quanri Zhang, Chul Han Kim, Un Yung Choi, Jeongsil Kim-Ha, Young-Joon Kim

**Affiliations:** 1 Department of Biochemistry, College of Life Science and Biotechnology, Yonsei University, Seoul 03722, Republic of Korea; 2 Department of Integrated Omics for Biomedical Science, Graduate School, Yonsei University, Seoul 03722, Republic of Korea; 3 Department of Integrative Bioscience and Biotechnology, College of Life Sciences, Sejong University, Seoul 05006, Republic of Korea

## Abstract

In response to persistent mycobacteria infection, the host induces a granuloma, which often fails to eradicate bacteria and results in tissue damage. Diverse host receptors are required to control the formation and resolution of granuloma, but little is known concerning their regulatory interactions. Here we show that Mincle, the inducible receptor for mycobacterial cord factor, is the key switch for the transition of macrophages from cytokine expression to high nitric oxide production. In addition to its stimulatory role on TLR-mediated transcription, Mincle enhanced the translation of key genes required for nitric oxide synthesis through p38 and eIF5A hypusination, leading to granuloma resolution. Thus, Mincle has dual functions in the promotion and subsequent resolution of inflammation during anti-mycobacterial defence using both transcriptional and translational controls.

## Abstract (web summary)

Resolution of granulomas in mycobacterial infection requires macrophages to switch from cytokine to nitric oxide (NO) production. Here the authors show that Mincle stimulates translation of the key NO synthesis genes by a mechanism dependent on p38-mediated hypusination of eiF5A.

## Graphical abstract

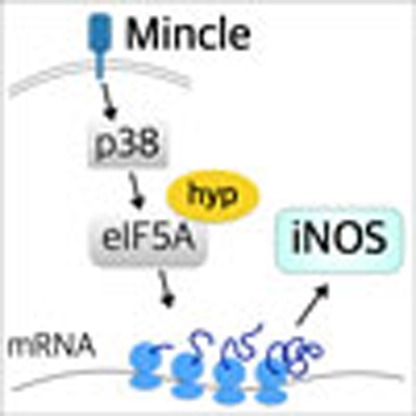

Mycobacteria are notorious pathogens that are difficult to treat^1^. Their unusual cell wall components allow them to survive harsh conditions, and they easily evade the host immune system by blocking the maturation of macrophage phagolysosomes^2^,^3^. In response to evasive mycobacterial infection, the host induces granuloma formation to limit the spread of mycobacteria^4^. A granuloma is mostly composed of activated macrophages. In an immunocompetent host, macrophages can control mycobacterial infection; thus, a small granuloma can proceed to granuloma-inflammation resolution. However, a granuloma in an immunodeficient host fails to control a high bacterial load and results in necrotic granuloma-inflammation with the release of free mycobacteria and high tissue damage^5^. Therefore, proper regulation of activated macrophages is important for immunocompetent granuloma-mediated anti-mycobacterial immunity. Several cytokines, including tumour necrosis factor α (TNFα) and interferon-γ (IFNγ), have been shown to promote granuloma formation^6^. However, the detailed mechanism leading to the alternative fates of granuloma is largely unknown.

Various host receptors can recognize mycobacteria and often induce a proinflammatory response^7^. However, depending on the environmental context of their activation, certain receptors produce anti-inflammatory signals, which benefit mycobacterial survival. Toll-like receptors (TLRs) detect diverse mycobacterial molecules and induce various proinflammatory cytokines and anti-bacterial effector molecules^8^. However, mannose receptors cause phagosome maturation arrest and inhibit the inflammatory response on activation by mannose-containing molecules of mycobacteria^9^. Moreover, there are also cases in which a single receptor is responsible for both proinflammatory and anti-inflammatory responses. Dendritic cell-specific intercellular adhesion molecule-3 grabbing non-integrin (DC-SIGN) not only recognizes α-glucan on mycobacteria, leading to the phagocytosis of mycobacteria by dendritic cells, but also induces anti-inflammatory cytokines such as interleukin (IL)-10 (ref. 10). Macrophage-inducible C-type lectin (Mincle) recognizes mycobacterial cell wall component trehalose-6,6′-dimycolate (TDM), and has been shown to induce pro-inflammation^11^. Overall, the outcome of mycobacterial infection depends on the interactions between the diverse host receptors and mycobacterial molecules. However, it remains unclear how these receptor-driven signals are integrated in the alternative granuloma development during the course of mycobacterial infection.

Among the various receptors, TLRs, particularly TLR2 and Mincle appear to be the key players in anti-mycobacterial immunity^12^. The mycobacterial cell wall is highly enriched with lipoproteins and trehalose mycolic acids, which are specific ligands for TLRs and Mincle, respectively. TLR2 signalling is mediated through myeloid differentiation primary-response protein 88 (MyD88) to nuclear factor kappa-light chain-enhancer of activated B cells (NFκB). TDM-bound Mincle initiates Fc receptor γ recruitment and induces spleen tyrosine kinase (Syk) signalling, leading to strong NFκB activation^11^,^13^. Eventually, these two activated receptor signalling pathways converge on NFκB enhancing further proinflammatory responses. However, Wevers *et al.*^14^ demonstrated a suppressive role of human Mincle in antifungal defence. Human Mincle degrades interferon regulatory factor 1 by activating the E3 ubiquitin-protein ligase Mdm2, thereby suppressing IL12A transcription. These observations demonstrate that two roles of Mincle, stimulation and resolution of inflammation, allow crosstalk with other receptor signalling events to achieve balanced immune responses. However, the regulating mechanism concerning these opposing functions of Mincle remains unclear.

Here, we examined the regulatory interactions between TLRs and Mincle in the control of granuloma formation and resolution. We found that Mincle enhanced the immune response by activating the transcription of proinflammatory genes. However, continued activation of TLRs and Mincle by persisting mycobacterial components inhibited the general translational machinery by 4EBP-1 dephosphorylation. Instead, Mincle-induced strong nitric oxide (NO) release by enhancing the translation of key enzymes via p38-dependent eIF5A hypusination, inhibiting Nod-like receptor protein 3 (NLRP3)-dependent IL-1β production. Moreover, genetic or chemical inhibition of Mincle-mediated translational control resulted in poor inflammation resolution and survival. Therefore, Mincle is essential not only for proinflammatory stimulation by enhancing NFκB signalling, but also for the translational control of specific transcripts required for NO production and inflammation resolution in macrophages.

## Results

### Mincle enhances the transcription of inflammatory genes

To examine the crosstalk between the TLR and Mincle signalling pathways, wild type (WT) and Mincle^−/−^ macrophages were stimulaed with their respective ligands, and their regulatory interactions were examined by transcriptome analysis. Pam3CSK4 (Pam3) treatment caused strong transcriptional changes (more than threefold) in 636 genes, mostly enriched in the innate immune response ontology group. Specifically, genes involved in leucocyte chemotaxis, and inflammatory response were highly activated, whereas genes involved in cell division and adhesion were suppressed (Supplementary Fig. 1a and Supplementary Table 1). In general, TDM co-treatment resulted in a transcription profile similar to that of Pam3-treated cells (Supplementary Fig. 2a). However, several genes were specifically up- or downregulated by TDM treatment; upregulated genes were predominantly involved in chronic inflammation, prostaglandin biosynthesis, NO-mediated signalling and wound healing, whereas downregulated genes were mostly in the type I interferon response and antiviral gene groups (Supplementary Fig. 1b and Supplementary Table 1). In addition, a comparison of differential levels of transcripts with TDM treatment (wt_Pam3 & TDM versus wt_Pam3) or Mincle deficiency (wt_Pam3 & TDM versus Mincle KO_ Pam3 & TDM) revealed high correlation (*R*^2^=0.7767; Supplementary Fig. 2b), confirming that most TDM-stimulated transcriptional changes were dependent on Mincle. Therefore, Mincle provides strong co-stimulation for TLR-induced immune signals and regulates additional anti-bacterial response genes.

### Mincle suppresses NLRP3-dependent caspase-1 activation

**Figure 1:**
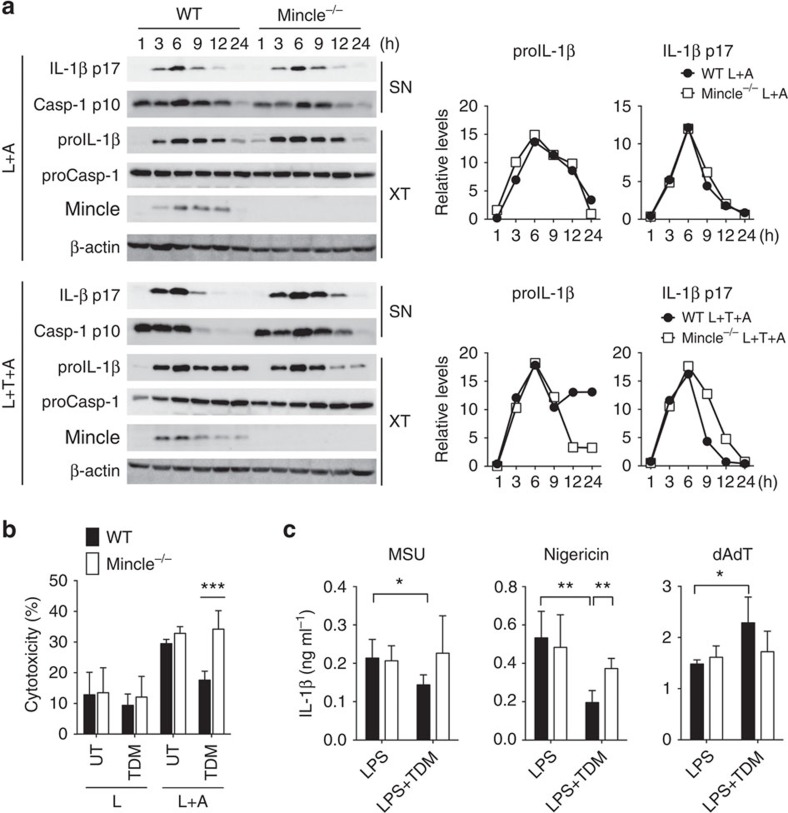
TDM stimulation diminishes NLRP3-dependent inflammasome activation and IL-1β production. (**a**,**b**) WT and Mincle^−/−^ BMDMs were stimulated with LPS or co-stimulated with LPS and TDM for 12 h or the indicated times and then treated with ATP for 1 h (L: LPS; T: TDM; A: ATP; UT: untreated). (**a**) Left: immunoblot analysis of proIL-1β, mature IL-1β (p17), procaspase-1 (proCasp-1) and cleaved caspase-1 (p10) in cell culture supernatants (SN) and whole-cell lysates (XT). Right: kinetic quantitative analysis of proIL-1β and mature IL-1β (p17) from the immunoblots. (**b**) Release of lactate dehydrogenase (LDH) (assessing cell death) from cells. (**c**) ELISA of released IL-1β from LPS-treated WT and Mincle^−/−^ BMDMs, stimulated with TDM for 12 h and then treated with MSU, nigericin or poly(dA:dT) for 3 h. *P<0.05, **P<0.01, ***P<0.001 (two-tailed unpaired *t*-test). Data are representative of at least three independent experiments. (**b**,**c**: mean and s.d.).

The stimulatory effect of Mincle on TLR signalling prompted us to examine its effect on inflammasome activation. To enhance inflammasome activation, exogenous ATP, a NLRP3 agonist, was added after varying durations of lipopolysaccharides (LPS) pretreatment^15^. LPS treatment enhanced proIL-1β messenger RNA (mRNA)/protein and enabled IL-1β maturation at 6 h after ATP addition (Fig. 1a and Supplementary Fig. 3). Although LPS treatment induced Mincle in WT macrophages, WT and Mincle^−/−^ macrophages showed no difference in inflammatory responses in the absence of the Mincle ligand. Moreover, TDM alone was not able to induce proIL-1β mRNA, not to mention protein expression, before 48 h of stimulation (Supplementary Fig. 3). In contrast to sole treatment of TDM or LPS, co-treatment of TDM and LPS before ATP addition induced stronger proIL-1β mRNA and protein expression, which continued up to 48 h in WT macrophages (Supplementary Fig. 3). Furthermore, despite high-precursor protein levels, IL-1β secretion was abruptly reduced after 6 h (Fig. 1a and Supplementary Fig. 4a). In comparison to WT macrophages, Mincle^−/−^ macrophages did not show extended proIL-1β mRNA and protein production, but produced much higher levels of mature IL-1β. Similar proIL-1β expression and IL-1β maturation were also observed with trehalose-6,6-dibehenate (TDB; a synthetic analogue of TDM) treatment (Supplementary Fig. 4b). However, the secretion levels of other cytokines, TNFα and IL-6, were not affected by TDM treatment or Mincle mutation (Supplementary Fig. 4c). In addition, caspase-1 maturation was similarly inhibited by TDM-mediated Mincle signalling (Fig. 1a). Therefore, TDM treatment is responsible for reduced pyroptotic cell death in WT macrophages (Fig. 1b). Taken together, these results demonstrate that TDM-mediated Mincle activation reduces NLRP3-inflammasome activation and cell death.

To determine whether Mincle specifically inactivates a certain type of inflammasome, we tested the effect of prolonged Mincle activation on IL-1β secretion induced by several NLRP3 or AIM2 agonists (Fig. 1c). IL-1β secretion, triggered by the NLRP3 agonists, monosodium urate crystals (MSU), nigericin, as well as extracellular ATP, was significantly inhibited by TDM in a Mincle-dependent manner. However, IL-1β induction by treatment with cytosolic double-stranded DNA poly(dA:dT), an AIM2 agonist, was not inhibited by Mincle signalling. This suggests that Mincle inhibits NLRP3-inflammasome activation.

### NO production by Mincle inhibits NLRP3-inflammasome

**Figure 2:**
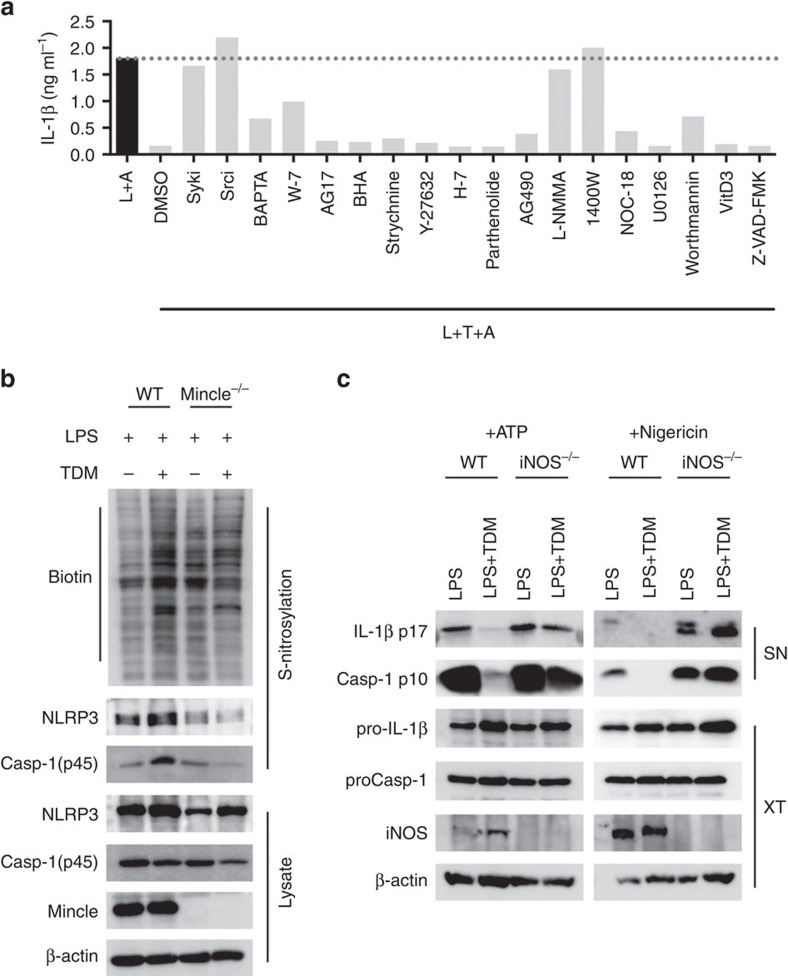
TDM-induced nitric oxide upregulation inhibits activation of the NLRP3 inflammasome. (**a**) WT and Mincle^−/−^ BMDMs were stimulated with LPS (L+A) or co-stimulated with LPS and TDM (L+T+A) for 12 h in the presence of the indicated chemical inhibitors, and then treated with ATP for 1 h. ELISA of released IL-1β. (**b**) Immunoblot analysis of total *S*-nitrosylated proteins (biotin), NLRP3 or caspase-1 in BMDMs treated with LPS or stimulated with TDM for 12 h. Below (lysate), immunoblot analysis of total lysate fractions. (**c**) Immunoblot analysis of IL-1β and caspase-1 from LPS-treated WT and iNOS^−/−^ BMDMs, stimulated with TDM for 12 h, and then treated with ATP for 1 h or nigericin for 3 h. Data are representative of one (**a**) or two (**b**,**c**) independent experiments.

To define the pathways involved in Mincle-mediated IL-1β suppression, we inhibited diverse signalling molecules with specific chemical inhibitors, and examined their effects on IL-1β production with Mincle activation (Fig. 2a). As expected from previous studies regarding the requirement of Src and Syk kinases for Mincle signalling^16^,^17^, inhibitors of Src (Srci) and Syk (Syki) effectively prevented the inhibitory effect of Mincle on IL-1β secretion (Fig. 2a). In addition, inducible nitric oxide synthase (iNOS) inhibitors (1400W and L-NMMA) strongly inhibited Mincle-mediated downregulation of IL-1β secretion, indicating NO as a mediator of IL-1β inhibition by Mincle (Fig. 2a). These inhibitors did not affect other immune signalling pathways leading to proIL-1β (and proCaspase-1) production (Supplementary Fig. 5a).

As shown previously^18^, Mincle is required for high NO production at the later stage of inflammation (Supplementary Fig. 5b,c). Although LPS treatment alone caused a slight increase in NO release due to its promiscuous signalling, TDM co-treatment with either TLR ligand further induced significant NO release in a Mincle-dependent manner. NO is known to inhibit IL-1β secretion by inactivating the inflammasome through NO-mediated *S*-nitrosylation of NLRP3^19^,^20^. Consistent with the high levels of NO release, we detected increased levels of Mincle-dependent *S*-nitrosylated proteins including NLRP3 and caspase-1 in TDM-treated cell extracts (Fig. 2b).

To determine whether Mincle-mediated inflammasome inactivation mainly depends on iNOS, we compared WT and iNOS^−/−^ macrophages and found that robust mature IL-1β production and caspase-1 cleavage following NLRP3-inflammasome activation with ATP or nigericin treatment were detected in iNOS^−/−^ macrophages, regardless of Mincle activation with TDM (Fig. 2c). Therefore, in the absence of iNOS expression, Mincle was not able to cause IL-1β downregulation.

### Mincle promotes the translational regulation of iNOS

**Figure 3:**
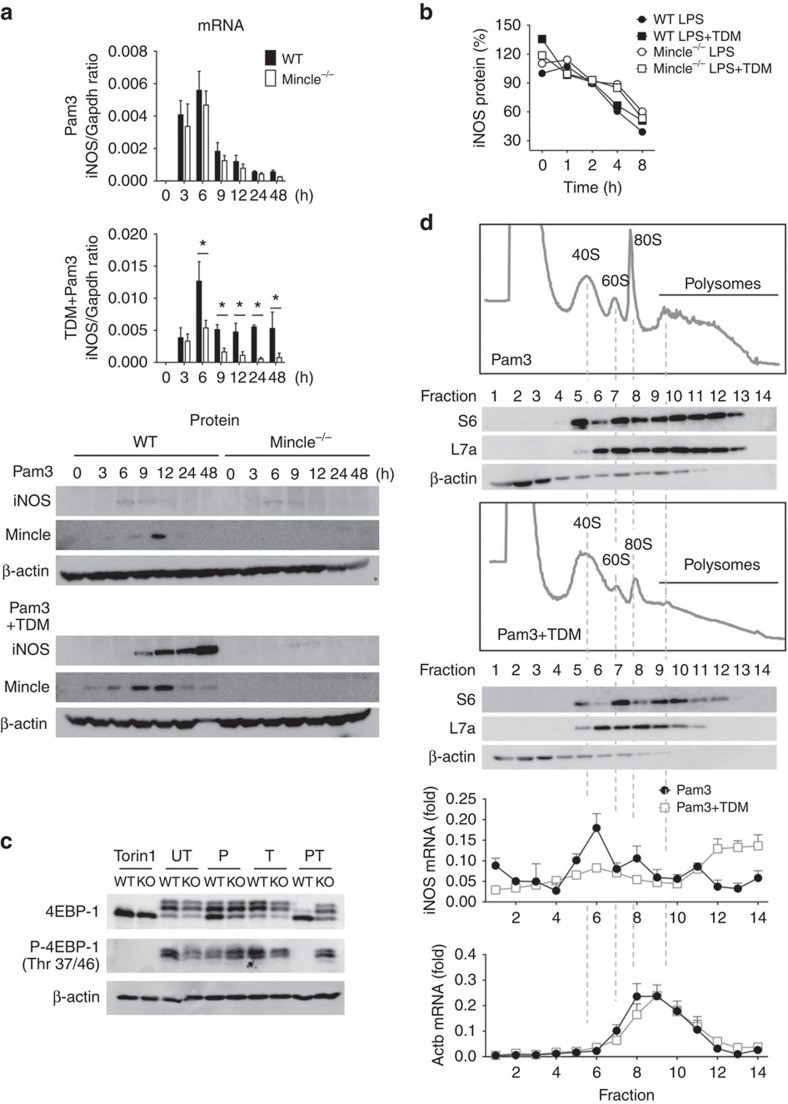
Mincle signalling results in efficient iNOS mRNA translation. (**a**) WT and Mincle^−/−^ BMDMs were stimulated with Pam3 or co-stimulated with Pam3 and TDM for the indicated times. Left: qRT-PCR analysis of iNOS mRNA levels from each type of stimulated macrophage; right: immunoblot analysis of iNOS protein expression. *P<0.05 (Student's *t*-test). (**b**) The half-life of the iNOS protein was measured from Supplementary Fig. 7. The ratio of iNOS signals from the actinomycin D- and cycloheximide-treated samples to those from samples treated only with LPS or co-treated with LPS and TDM for 6 h were calculated and plotted as percentages (initial value set at 100%). (**c**) Immunoblot analysis of 4EBP-1 and specific phosphor-4EBP-1 (Thr 37/46) from WT and Mincle^−/−^ BMDMs treated with Torin1, Pam3 (P), TDB (T) or co-treated with Pam3 and TDB (PT) for 24 h. UT, untreated. (**d**) Polysome profiles and immunoblot analysis of the S6 (small) and L7a (large) ribosomal subunit components and β-actin (nonribosomal protein) in lysates of WT macrophages stimulated with Pam3 or co-stimulated with Pam3 and TDM for 12 h. qRT-PCR analysis of iNOS (left) and β-actin (right) mRNA in polysome fractions from WT macrophages stimulated with Pam3 or co-stimulated with Pam3 and TDM, presented for each fraction relative to the sum of all 14 fractions. Data are representative of at least three independent experiments (**a**,**b**,**d**: mean and s.d.).

To understand how Mincle stimulates iNOS expression, we examined iNOS mRNA and protein levels in WT and Mincle mutant macrophages following stimulation. Pam3 treatment increased iNOS mRNA, which peaked at 6 h in both WT and Mincle^−/−^ macrophages (Fig. 3a). However, iNOS protein was not detectable, hinting that these mRNA may not be actively translated. TDM co-treatment dramatically enhanced iNOS mRNA and protein in a Mincle-dependent manner, and iNOS protein was gradually increased even 48 h after stimulation, temporally distinct from the mRNA peak. Although moderate levels of iNOS mRNA were induced by the treatment, only slight amount of iNOS protein was detected in Mincle^−/−^ macrophages, demonstrating the requirement of Mincle for iNOS protein expression. Because LPS activates diverse immune signalling pathways^21^, LPS treatment elicited strong iNOS mRNA production independent of Mincle, and triggered iNOS protein synthesis (Supplementary Fig. 6). Nevertheless, TDM co-stimulation further extended iNOS protein synthesis beyond 24 h, only in WT macrophages. These results suggest possible post-transcriptional regulation of iNOS by Mincle.

To find out the mechanism of enhanced-iNOS protein level by Mincle, we tested iNOS protein stability in the presence of both transcriptional and translational inhibitors. Although the chemical treatment of cycloheximide with actinomycin D might influence protein stability indirectly, iNOS was degraded at the same rate (50% reduction in about 8 h) in all conditions tested. These results suggest that the half-life of iNOS was not mainly affected by TDM co-stimulation or Mincle deficiency (Fig. 3b and Supplementary Fig. 7).

To test whether Mincle regulates iNOS translation, we first examined the effect of prolonged TLR and Mincle stimulation on two aspects of general translation: polysome fraction enrichment^22^ and 4EBP-1 phosphorylation. 4EBP-1 binds and negatively regulates eIF-4E, a key rate-limiting initiation factor for translation^23^. Phosphorylation of 4EBP-1 by mammalian target of rapamycin (mTOR) release eIF-4E, allowing translation initiation, so the general translational activity can be monitored with phosphorylation status of 4EBP-1 (ref. 24). Pam3 treatment retained 4EBP-1 phosphorylation, allowing translational initiation in both WT and Mincle^−/−^ macrophages (Fig. 3c). Consistent with the retained levels of 4EBP-1 phosphorylation, polysome fractions were highly enriched with transcripts along with a strong 80S peak under Pam3-stimulation condition (Fig. 3d). However, prolonged co-stimulation of Pam3 and TDM for 12 h suppressed the transcripts in the polysome fraction. Instead, transcripts associated with translationally arrested 40S fractions were increased, indicating global downregulation of polysome in Mincle-activated macrophages (Fig. 3d). In addition, 4EBP-1 became dephosphorylated as the co-stimulation continued more than 24 h (Fig. 3c and Supplementary Fig. 8a), as showing identical patterns of 4EBP-1 dephosphorylation by Torin1, an mTOR inhibitor^25^ (Fig. 3c). Addition of iNOS inhibitor (1400W) or NO donor (SNAP) during co-stimulation had no effect on 4EBP-1 dephosphorylation (Supplementary Fig. 8b), ruling out the connection between NO production and mTOR inhibition. However, the distribution pattern of an individual iNOS mRNA along the polysome analysis fractions showed the opposite trend of association from other mRNA (Fig. 3d). iNOS mRNA was predominantly found in translationally arrested subribosomal fractions of Pam3- (or LPS)-treated macrophages, while TDM co-treatment with either TLR ligand shifted it to translationally active heavy polysome fractions (Fig. 3d and Supplementary Fig. 9b). Mincle^−/−^ macrophages showed low level of iNOS mRNA compared to that of WT, and its association with the polysome fraction on TDM treatment was not apparent (Supplementary Fig. 9a,b). These data demonstrated that TDM-induced Mincle promotes translational upregulation of iNOS mRNA. As a control, the distribution patterns of the Actb mRNA, a housekeeping gene, were not affected by the types of ligand treatment nor by the absence of Mincle in the cells. On the other hand, most RNAs including Rps28 mRNA, a ribosomal protein transcript, were shifted from polysome (fractions 8–10) to monosome fractions (fractions 4–6) after co-stimulation of Pam3 and TDM (Supplementary Fig. 10). Therefore, while co-stimulation of TLRs with Mincle appears to inhibit general translation, Mincle selectively enhanced iNOS mRNA translation in activated macrophages.

### Mincle-mediated iNOS translation requires for p38 activation

**Figure 4:**
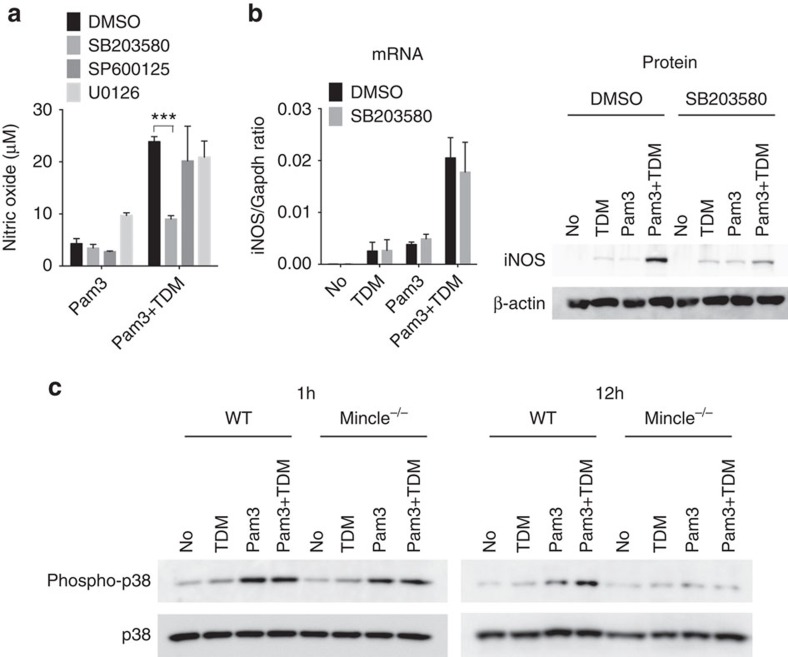
Mincle-induced p38 activation is required for iNOS translation. (**a**,**b**) WT BMDMs were stimulated with TDM or Pam3 or co-stimulated with Pam3 and TDM in the presence of the indicated chemical inhibitors, for 12 h. (**a**) Nitric oxide production in culture supernatants. ***P<0.001 (Student's *t*-test). (**b**) left: qRT-PCR analysis of iNOS mRNA levels from stimulated macrophages; right: immunoblot analysis of iNOS protein expression. (**c**) Immunoblot analysis of phospho-p38 from WT and Mincle^−/−^ BMDMs treated with Pam3, TDM or co-stimulated with Pam3 and TDM for 1 or 12 h. Data are representative of at least three independent experiments.

We previously found that TDM-induced Mincle signalling activates MAP kinases via Syk^17^. So we investigated whether these MAP kinases were involved in regulating iNOS translation. MAP kinase inhibitors had no effect (p38 inhibitor, SB203580 and Jnk inhibitor, SP600125) or enhanced (Erk inhibitor, U0126) NO release under Pam3 stimulation. However, the p38 inhibitor, SB203580 strongly and specifically inhibited NO release induced by Pam3 and TDM co-treatment (Fig. 4a). SB203580 appears to inhibit post-transcriptionally iNOS protein expression, but not mRNA expression, induced by Pam3 and TDM co-stimulation (Fig. 4b). Similarly, SB203580 also blocked TDM-dependent iNOS protein expression under LPS and TDM co-stimulation (Supplementary Fig. 11a,b). In addition, another p38 selective inhibitor, PD169316 also suppressed iNOS protein expression specifically (Supplementary Fig. 12a,b). Therefore, p38 activation appears to be required for Mincle-mediated iNOS protein expression.

Because p38-mediated translational control occurred long after TDM stimulation (over 12 h), we wondered whether p38 activation is directly involved in iNOS translation, and examined the p38 phosphorylation profile after Mincle stimulation. Phosphorylation of p38 was highly induced 30 m after TLR2 (or TLR4) stimulation and turned off within 6 h (Supplementary Fig. 13). However, TDM co-treatment extended p38 phosphorylation up to 24 h, until the later activation stage, overlapping with iNOS protein expression. This extended p38 phosphorylation was dependent on Mincle activation, because only WT macrophages co-stimulated with TDM and Pam3 showed high levels of extended p38 phosphorylation for more than 12 h (Fig. 4c). As Mincle expression was induced by Pam3 after 6 h (Fig. 3a), a second wave of p38 phosphorylation by Mincle may lead to a prolonged phosphorylation of p38 and iNOS protein expression at later stages.

### eIF5A^hyp^ by Mincle is critical for iNOS mRNA translation

**Figure 5:**
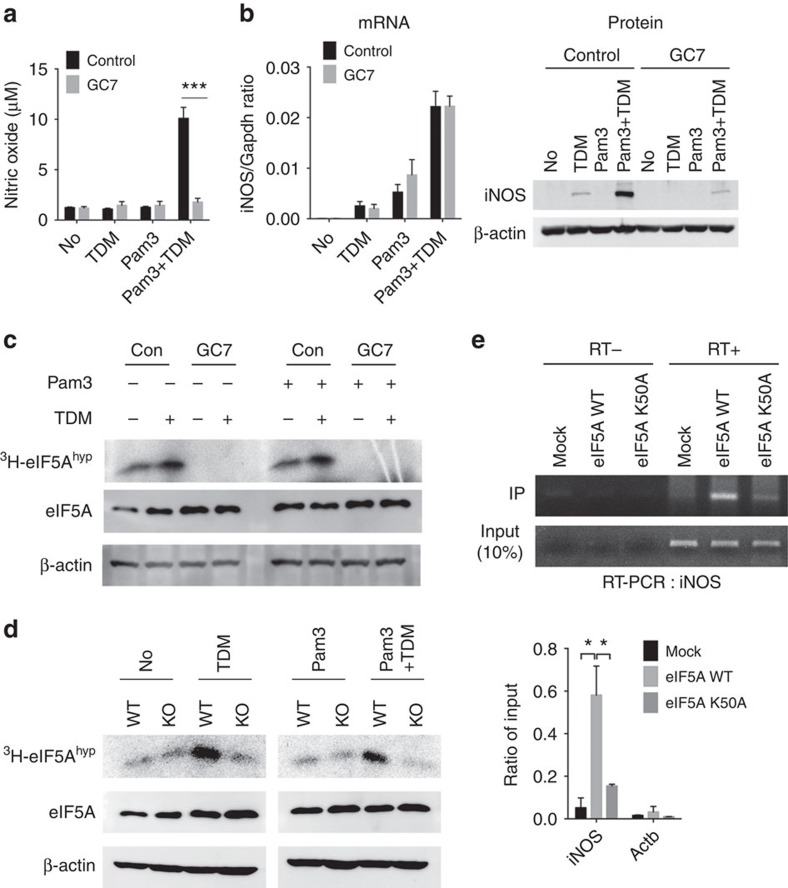
eIF5A hypusination is required for Mincle-mediated iNOS translation. (**a**,**b**) WT BMDMs were stimulated with TDM, Pam3 or co-stimulated with Pam3 and TDM in the presence of GC7 for 12 h. (**a**) Nitric oxide release in culture supernatants. (**b**) qRT-PCR (left) and immunoblot (right) analysis of iNOS mRNA or protein expression. (**c**) Fluorographic analysis of hypusinated eIF5A from WT BMDMs treated with Pam3, TDM or co-treated with Pam3 and TDM in the presence of control (con) or GC7. (**d**) Fluorographic analysis of hypusinated eIF5A from WT and Mincle^−/−^ (KO) BMDMs treated with Pam3, TDM or co-treated with Pam3 and TDM. (**e**) RIP analysis of iNOS and Actb mRNAs from NIH-3T3 cells co-transfected with expression vector for Flag alone (Flag-Mock) or Flag-tagged eFI5A WT or K50A mutant, plus an iNOS mRNA expression vector. Agarose gel electrophoresis (top) and qRT-PCR analysis (bottom) for the indicated genes. Data are expressed as per cent recovery relative to input RNA. *P<0.05, ***P<0.001 (Student's *t*-test). Data are representative of three (**a**,**b**,**e**) or two (**c**,**d**) independent experiments (**a**,**b**,**e**: mean and s.d.).

Previously, iNOS translation was shown to be regulated by p38-dependent eIF5A hypusination in islet β-cells^26^,^27^. This led us to ask whether the apparent translational effect of the Mincle-p38 axis might be related to eIF5A function. The hypusine modification of eIF5A is processed sequentially by two enzymes, deoxyhypusine synthase (DHS) and deoxyhypusine hydroxylase (DOHH)^28^. First, we examined the effect of DHS inhibition by GC7 (*N*′-guanyl-1,7-diaminoheptane, a DHS inhibitor) on Mincle-dependent NO synthesis. GC7 completely inhibited NO release (Fig. 5a and Supplementary Fig. 14a) and iNOS protein expression, but not mRNA expression, when induced by co-stimulation of TDM and Pam3 (Fig. 5b) or co-stimulation of TDM and LPS (Supplementary Fig. 14b). These results indicate the involvement of eIF5A hypusination in iNOS expression in activated macrophages. To confirm Mincle-dependent eIF5A hypusination, we added ^3^H-spermidine to the bone marrow-derived macrophage (BMDM) culture and examined eIF5A hypusination (eIF5A^hyp^) under various stimulation conditions. Basal levels of eIF5A^hyp^ in BMDMs were highly stimulated by TDM in a Mincle-dependent manner, but completely inhibited by the addition of GC7 or p38 inhibitor (Fig. 5c,d and Supplementary Fig. 14c,d). We obtained a similar result when eIF5A hypusination was inhibited by ciclopirox (CPX), a DOHH inhibitor^29^,^30^. CPX inhibited NO release and iNOS protein expression from the macrophages stimulated with Pam3 and TDM, without causing a defect in iNOS mRNA expression (Supplementary Fig. 15a,b). Therefore, these results demonstrate that Mincle-p38-mediated eIF5A hypusination is required for iNOS translation in activated macrophages.

To determine the specificity of eIF5A-mediated translational regulation, we examined the direct association of iNOS mRNA with eIF5A using RNA immunoprecipitation (RIP) followed by quantitative reverse transcription-coupled PCR(qRT-PCR) assays (Fig. 5e). WT eIF5A specifically bound to significant amounts of iNOS mRNA, but the hypusine modification-defective eIF5A-K50A mutant was not able to bind iNOS mRNA. As a control, β-actin (Actb) mRNAs did not immunoprecipitate with either WT or K50A eIF5A. Therefore, Mincle-dependent hypusination of eIF5A is required for iNOS mRNA binding, suggesting Mincle is the main regulator of iNOS translation.

To examine the specificity of the Mincle-mediated regulation, we tested several pathogen-associated ligands and IFNγ for their ability to regulate iNOS expression. TDM, Poly(I:C) and IFNγ induced strong NO production when co-treated with Pam3, but, GC7 treatment inhibited NO production induced only by TDM, but not by poly(I:C) and IFNγ. Unlike TDM, poly(I:C) and IFNγ appeared to stimulate iNOS expression mainly through the mRNA augmentation (Supplementary Fig. 16a,b). These results indicate that Mincle-induced NO production is distinct from those depending on other ligands, such as IFNγ and poly(I:C).

### Polysome profiling reveals Mincle-eIF5A^hyp^-regulated genes

**Figure 6:**
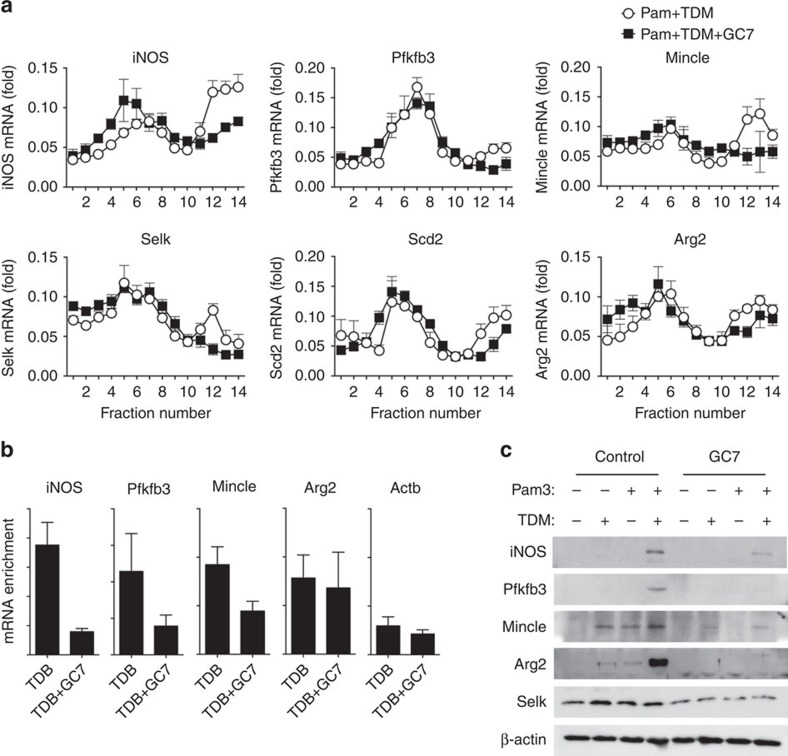
Specific genes selected from the polysome profiling assay are regulated by Mincle-eIF5A-dependent translation. (**a**) Polysome fraction mRNAs from WT BMDMs co-treated with Pam3, TDM, with or without GC7 were analysed. qRT-PCR analysis of the indicated mRNAs in cells treated as indicated, presented for each fraction relative to the sum of all fourteen polysome fractions. (**b**) RIP analysis of the indicated gene transcripts from iBMDM cells stably expressing Flag-eIF5A, stimulated as indicated. Immunoprecipitation with anti-Flag antibody and qRT-PCR analysis of the indicated genes. (**c**) WT BMDMs were stimulated with Pam3, TDM or co-stimulated with Pam3 and TDM, in the presence of GC7 or control vehicle for 12 h. Immunoblot analysis of the indicated protein expression levels. Data are representative of three independent experiments (**a**–**c**: mean and s.d.).

To identify additional transcripts regulated by Mincle-dependent eIF5A hypusination, we examined RNAs preferentially enriched in the polysome fraction under TDM and Pam3 co-stimulation, but shifted to the subpolysome fractions when GC7 was added. To this end, we sequenced total and polysome-associated RNAs from Pam3 and TDM co-treated macrophages with or without GC7 (Supplementary Fig. 17a,b). We observed 282 mRNAs located on the polysome by Pam3 and TDM co-stimulation; among these, 111 mRNAs were depleted from polysome fractions on GC7 treatment, revealing small number of transcripts highly translated in the activated macrophages, possibly with the help from hypusine (Supplementary Fig. 18a). To validate their Mincle and eIF5A^hyp^-dependent polysome association, we examined the ratios of polysome-associated versus monosome-associated transcripts (P/M ratios) induced by TDM and Pam3 co-treatment with/without GC7 by qRT-PCR. Among 20 relatively abundant candidate transcripts tested, six transcripts (iNOS, Mincle, Arg2, Pfkfb3, Selk and Scd2) showed significant decrease in P/M ratios (Supplementary Fig. 19a) as well as eIF5A^hyp^-dependent polysome association in GC7-treated cells (Fig. 6a and Supplementary Fig. 19b). In addition to iNOS and Mincle, these genes are involved in high NO production and cell survival: arginase 2 (Arg2) catalyzes arginine and plays important role in NO and polyamine metabolism^31^. The key enzyme for glycolysis, 6-phosphofructo-2-kinase/fructose-2,6-biphosphatase 3 (Pfkfb3), is the main energy source of macrophages^32^. Selenoprotein k (Selk) is required for Ca^2+^ flux from endoplasmic reticulum (ER) during immune response^33^. Steroyl-Coenzyme A desaturase 2 (Scd2) controls intracellular level of monounsaturated fatty acids in immune cells, which is important for inflammation^34^. Except for Selk, mRNA of the polysome-associated transcripts identified above (iNOS, Pfkfb3, Arg2 and Mincle) were abundantly induced by Mincle (Supplementary Fig. 18a,b).

We further investigated their interaction with eIF5A^hyp^ using RIP with Flag-tagged eIF5A. eIF5A specifically co-precipitated significant amounts of endogenous iNOS, Pfkfb3, Mincle mRNAs, except for Arg2 and Actb mRNAs, in the absence of GC7, confirming their direct interaction with eIF5A (Fig. 6b). Western blot analysis confirmed high levels of protein expression of iNOS, Pfkfb3, Arg2 and Mincle only in the Mincle-activated cells in the absence of GC7, despite the high mRNA levels, as seen with iNOS proteins (Fig. 6c and Supplementary Fig. 18b). Although Selk mRNA was not induced under TDM with Pam3 co-stimulation condition, its protein expression was decreased significantly after GC7 treatment, supporting the eIF5A^hyp^-dependent translational regulation. Therefore, Mincle-eIF5A controls the translation of several specific transcripts required for NO production in mycobacteria-stimulated macrophages.

### Inhibition of eIF5Ahyp inflames TDM-induced lung granuloma

**Figure 7:**
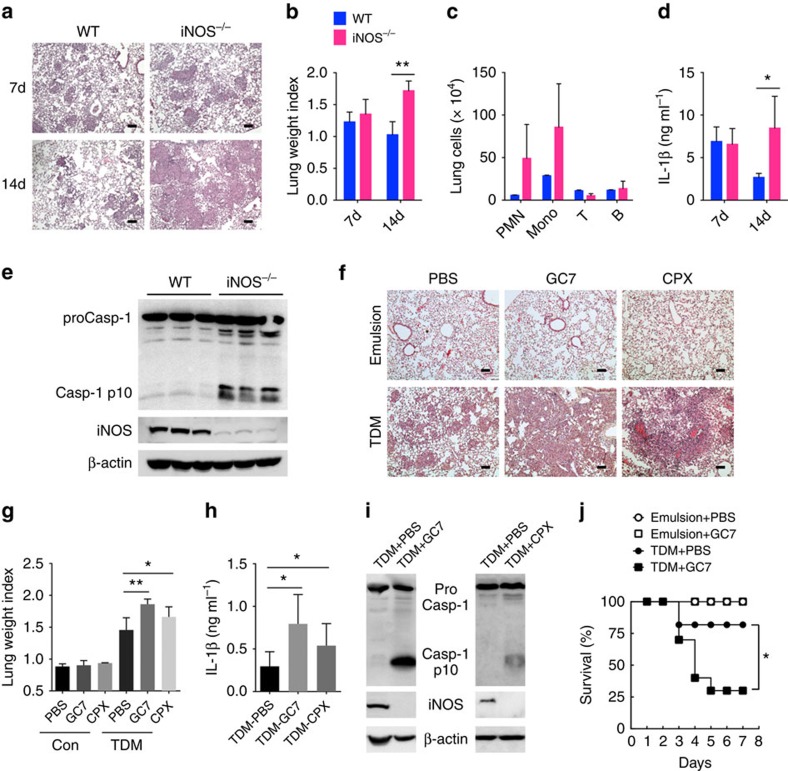
iNOS deficiency and inhibition of eIF5A hypusination aggravate granuloma formation by TDM. (**a**–**e**) WT and iNOS^−/−^ mice were injected intravenously with an oil-in-water emulsion of TDM (*n*>6 mice/group). (**a**) Histology of lungs at 7 and 14 days after injection (scale bars, 100μm). (**b**) TDM-induced lung swelling. On days 7 and 14 after injection of TDM, lung swelling was evaluated by LWI. (**c**) Identification of leucocyte subsets in lung granulomas on day 14 after TDM injection by flow cytometry. The number of neutrophils (PMN, CD11b^+^ Ly6G^+^), monocytes (Mono, CD11b^+^ Ly6G^−^), T cells (CD3^+^) and B cells (CD19^+^) are indicated. (**d**) ELISA of IL-1β in lung lysates on day 7 or 14 after TDM injection. (**e**) Immunoblot analysis of active caspase-1 (Casp1 p10) and iNOS in lysates of lungs from WT and iNOS^−/−^ mice injected with TDM; β-actin serves as a loading control (each lane represents an individual mouse). (**f**–**j**) Mice were administered with PBS, GC7 or CPX via intraperitoneal injection, and with oil-in-water emulsion of TDM or emulsion alone, via intravenous injection. On day 7 after the indicated injection, lungs were harvested and experiments were performed. (**f**) Lung histology was examined by H&E staining (scale bars, 100 μm.) (**g**) TDM-induced lung swelling. At day 7 after the indicated injection, lung swelling was evaluated by LWI. (**h**) ELISA of IL-1β in lung lysates on day 7 TDM injection. (**i**) Immunoblot analysis of active caspase-1 (Casp1 p10) and iNOS in lysates of lungs from mice injected with TDM with/without GC7 or CPX, assessed on day 7 after injection; β-actin serves as a loading control (each lane represents an individual mouse). (**j**) Lethal systemic inflammation by TDM (*n*=five mice/group, *P*-value calculated by Mantel–Cox test). *P<0.05, **P<0.01 (two-tailed unpaired Student's *t*-test). Data are representative of two experiments (**b**–**d**,**g**–**i**: mean and s.d. of three to five mice per group).

A granuloma is an organized collection of immune cells, mostly macrophages, induced by sustained presence of mycobacteria before killing them^35^. Excessive granuloma formation by high IL-1β would result in severe tissue damage due to uncontrolled necrotic cell death^36^,^37^,^38^. Thus, delayed NO production following IL-1β activation may contribute not only to bacterial killing, but also to anti-inflammation by downregulating IL-1β. To confirm the negative regulatory function of NO in the inflammatory response, we injected TDM intravenously with oil-in-water emulsion, and compared the extent of pulmonary granuloma formation in WT and iNOS^−/−^ mice. Both WT and iNOS^−/−^ mice showed an increase in lung weight index (LWI) and granuloma formation at day 7 after TDM injection (Fig. 7a,b). Similar to previous reports^39^, TDM-induced granuloma was resolved and partially disappeared 14 days post administration in the WT lung. However, the granuloma area and LWI were increased in iNOS^−/−^ mice, rather than being resolved. Consistent with the severe histopathology, iNOS^−/−^ mouse lungs at day 14 showed significantly increased infiltration of neutrophil (CD11b^+^Ly6G^+^) and monocyte (CD11b^+^Ly6G^−^), but not lymphocyte (Fig. 7c). Although we cannot rule out an indirect effect caused by NO deficiency, this pathological damage appeared to be caused by increased inflammasome activation: increased IL-1β secretion and cleavage of caspase-1 was observed in iNOS mutant mouse lungs (Fig. 7d,e). Therefore, iNOS is required for IL-1β downregulation after the confinement of mycobacterial substances within the granuloma to prevent the excessive spread of inflammatory response.

Next, we examined whether blocking eIF5A hypusination would have similar effects on TDM-induced granuloma formation. Although intraperitoneal injection of GC7 alone or CPX alone elicited no discernible immune responses, co-injection of GC7 with TDM, or CPX with TDM caused severely aggravated inflammatory responses. Granuloma formation, LWI, IL-1β secretion and cleavage of caspase-1 were significantly increased, along with decreased iNOS expression in the lungs of TDM with GC7, or TDM with CPX co-injected mice, probably due to blocked eIF5A hypusination (Fig. 7f–i). Eventually, these hyper-responses under TDM-induced inflammatory conditions provoked severe lethality in GC7-injected mice (Fig. 7j). These lines of evidence demonstrate that hypusination of eIF5A by Mincle activation is required for autonomous feedback control of the TDM-mediated inflammatory response in innate immune cells.

## Discussion

The evasive nature of mycobacteria inside macrophages makes them difficult to eradicate, leading to persistent pathogen signals and subsequent immunopathogenic damage^1^. In response, hosts form granulomas to kill persisting bacteria inside macrophages while limiting inflammatory damage^4^,^5^. However, the regulation process of immune activation and suppression during the anti-mycobacterial response is not well understood. Mincle is the major pattern recognition receptor for the mycobacterial cell wall component TDM and activates proinflammatory cytokine expression^11^,^17^,^40^,^41^. Therefore, identification of Mincle as a key late-stage regulator of immune suppression is intriguing, and we find its pivotal role in the cycle of immune activation and suppression to control the anti-mycobacterial immune response. In addition, we present several lines of evidence showing distinct functions of Mincle geared to optimize the anti-mycobacterial immune response during immune activation and resolution.

First of all, Mincle-dependent NO production suppresses the inflammasome during inflammation resolution. Although IL-1β secretion is not significantly different in the early phase, Mincle^−/−^ macrophages showed much higher IL-1β production and caspase-1 cleavage during inflammation resolution than WT macrophages. This suggests that Mincle is involved in inhibiting late-stage NLRP3-inflammasome activation to prevent excessive inflammation. Therefore, Mincle plays a key role in the transition from inflammation to NO-mediated cytotoxicity and necroptosis, leading to optimal eradication of mycobacteria with inflammation resolution.

Moreover, high-level NO production is dependent strictly on Mincle signalling. Although iNOS mRNA was increased by TLR signalling, iNOS protein was undetectable until robust Mincle activation. Concerning the fact that cap-dependent translational initiation machinery of stressed macrophages is generally inhibited via eIF-4E sequestration by 4EBP due to deficient mTOR signalling^23^,^24^, robust iNOS translation under the same stressed conditions indicates the presence of a specialized regulatory mechanism for iNOS translation by Mincle. There are three transcript variants encoding two different isoforms in iNOS gene, but we could not definitively prove whether translation of different iNOS mRNA isoforms is differentially affected by Mincle signalling. However, we found that Mincle enhanced iNOS translation through a mechanism requiring p38-dependent eIF5A hypusination. Although eIF5A normally has no discernible effect on protein synthesis, it is essential for protein synthesis under stress, such as oxidative stress, apoptosis and hypoxia, in several cancer cells^42^,^43^. Therefore, Mincle appears to utilize stress-resistant translational control to induce high levels of cytotoxic NO at the late stage of the immune response.

Furthermore, Mincle is designed to handle persisting pathogenic signals. Mincle is induced in macrophages on TLR stimulation and reaches maximum induction after 12 h. In the absence of TLR stimulation, TDM treatment alone did not induce detectable inflammatory response from BMDM. Therefore, most early responses are triggered by TLR signalling, but strengthened by additional Mincle signalling if pathogenic ligands are still present. In addition, Mincle signalling is geared to handle cellular processes under the stress of a prolonged immune response. Genes required to handle such stress (for example, genes involved in glycolysis, ER stress response and cellular responses to reactive oxygen and hypoxia) are enriched specifically in TLR- and Mincle-codependent genes (Supplementary Table 1).

Contrary to our observation, previous studies showed a stimulatory effect of TDM by itself on NLRP3-inflammasome activation and IL-1β secretion^44^,^45^. However, this difference may be resulted from the different cell types (BMDM versus bone marrow-derived dendritic cells (BMDC)) or different TDM presentation methods (plate-coated form versus particulate form) used in the assays. Indeed, Desel *et al.*^45^ showed that only the particulate form of TDB could induce IL-1β secretion from BMDCs. These results indicate that phagocytosis of particulate form of TDB may provide additional signals required for IL-1β activation.

Second, genome-wide polysome profiling assay revealed a small group of transcripts specifically regulated by Mincle-eIF5A-dependent translational control. By analyzing polysome profiles, we confirmed that at least three mRNAs, generally known to be required for the high metabolic activity of activated macrophages, are specifically regulated by the Mincle-eIF5A axis. iNOS is the major metabolic enzyme required for NO production^31^. Pfkfb3 is a rate-limiting enzyme in the glycolytic pathway and catalyzes the production and degradation of fructose-2,6-bisphosphate, an allosteric activator of the key glycolytic enzyme 6-phosphofructo-1-kinase^32^,^46^. In immune cells, Pfkfb3 activates the glycolytic pathway to generate sufficient energy for high levels of NO generation. Pfkfb3 has also been suggested to activate autophagic machinery^47^. Interestingly, autophagy contributes to IL-1β production inhibition by eliminating active inflammasomes^48^. In addition, the translation of Mincle itself is regulated by Mincle-dependent eIF5A hypusination, enabling continued synthesis of its own protein for late function.

Although the direct interactions with eIF5A^hyp^ remained to be tested, several studies have suggested that steroyl-coenzyme A desaturase is involved in the protection of inflammation. Scd1 inhibition promotes atherosclerosis and raises LPS-induced proinflammatory responses in peritoneal macrophages^49^. In addition, Scd1 activity reduces lipid-induced ER stress responses in macrophages, indicating the regulation of macrophage-induced inflammation^34^,^50^. Similarly, Selk is required for the palmitoylation of CD36 and foam cell formation^51^. Selk has been shown to protect macrophages from oxidative stress and ER stress-induced apoptosis^33^. Mincle signalling did not activate Scd2 and Selk at the transcription level, but the enhanced translation of these genes may protect the activated macrophages from ER stress to produce NO and repair inflammation-associated damage.

The identification of Arg2 as a putative target of Mincle-mediated expression in the activated macrophages is particularly intriguing. Arginase 1 turns L-Arg into ornithine, thus reducing NO production by limiting the common substrate in macrophages^52^. However, Arg2 is highly expressed together with iNOS and is required for the expression of proinflammatory cytokines and iNOS in LPS-induced macrophages^53^. Although direct interaction between Arg2 mRNAs and eIF5A^hyp^ was not confirmed, its expression was highly activated by Mincle, probably by the activated p38 as shown previously^54^. The function of Arg2 in activated macrophages is not clear, but it may contribute to iNOS expression by generating a polyamine pool required for hypusine synthesis.

Third, Mincle sustains p38 phosphorylation until the late immune response. This prolonged p38 activation is responsible for Mincle-dependent translational control during inflammation resolution. We previously demonstrated p38, Jnk and Erk phosphorylation in response to mycobacterial cord factor in neutrophils^17^. This phosphorylation is usually turned off in a few hours; however, Mincle-induced p38 phosphorylation extends for days, sufficient to mediate eIF5A hypusination during the late immune response. Hypusine modification is known to occur only at eIF5A^55^, and is processed by two enzymatic steps, involving DHS and DOHH^28^. However, whether p38 directly targets these enzymes is unknown. Consistent with our results, Nishiki *et al.*^27^ showed that p38 inhibition reduced hypusination in β-islet cells. How Mincle sustains late p38 phosphorylation, and identifying the targets of activated p38, remains to be addressed.

Finally, RNA-seq analysis revealed that Mincle signalling at the initial stage synergistically modulates the transcription of most TLR2-regulated genes toward anti-mycobacterial responses. The Pam3 and TDM-activated macrophages resemble the phenotype of M1 macrophages with the expression of iNOS, IL-12 and CCL2 (ref. 56). Interestingly, Mincle further suppressed genes involved in type I interferon responses, indicating reinforcement of anti-mycobacterial functions. Since type I IFNs (IFN-α, IFN-β) are potent antiviral signalling molecules and inhibit the inflammatory pathways by limiting bioactive IL-1 production^57^,^58^, their regulation is required for host resistance against mycobacterial infection. Moreover, Mincle signalling further increased gene expression in the prostaglandin biosynthesis ontology group, consistent with previous observations that IL-1-mediated PGE2 suppresses type I interferon and limits mycobacterial growth^59^. In addition, genes involved in anti-inflammation (IL-4ra, IL-6, IL-10 and IL-13ra) and wound healing (Mmp9, Mmp 12, Mmp 13 and Timp1) were strongly enhanced by Mincle stimulation. These genes are usually associated with M2 macrophages that promote tissue repair^52^,^56^. Therefore, Mincle potentiates the initial inflammatory response by synergistically enhancing the transcription of innate immune genes related to anti-mycobacterial responses. Furthermore, Mincle triggers the transition of macrophage polarity from an inflammatory to a repairing phenotype.

**Figure 8:**
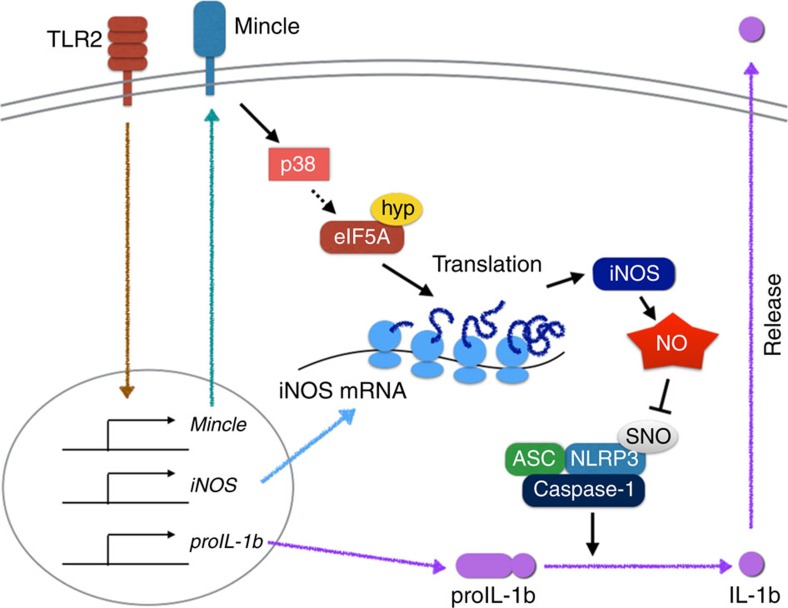
Model explaining Mincle-mediated translational regulation for nitric oxide production and inflammation resolution. In this model, Mincle is essential not only for transcription of proinflammatory genes, but also for translation of the key NO synthesis genes. Mincle promotes strong NO release by enhancing iNOS translation via a mechanism dependent on p38-mediated hypusination of eIF5A, inhibiting NLRP3 inflammasome and caspase-1-dependent IL-1β production. ASC, Apoptosis-associated speck-like protein containing a caspase recruitment domain; Hyp, hypusination; SNO, S-nitrosylation.

In summary, in addition to the stimulatory effect of Mincle on TLR signalling, our study provides evidence that TDM-induced Mincle signalling promotes the p38- and eIF5A-dependent translational regulation of specific genes including iNOS, which relieve inflammation by inhibiting IL-1β production (Fig. 8). Mincle was initially identified as an activating receptor for inflammation induced by cord factor, well known as an adjuvant. Although Mincle's function in suppressing inflammation is unexpected and initially counterintuitive, our findings demonstrate the pivotal role of Mincle in maintaining balanced immune responses for effective mycobacterial eradication without significant immunopathological damage.

## Methods

### Mice

C57BL/6 mice, aged 6–10 weeks, were purchased from Orient Bio (Gwangju, Gyeonggi, Korea). Mincle^−/−^ mice (Clec4e^MNA^) were kindly provided by the Consortium for Functional Glycomics (http://www.functionalglycomics.org) and were backcrossed for 10 generations to the C57BL/6 background. iNOS^−/−^ mice (B6.129P2-Nos2^tm1Lau^/J) were purchased from The Jackson laboratory (Bar Harbor, ME). iNOS^−/−^ mice and their WT control littermates were injected with TDM intravenously at 6–11 weeks of age, and both sexes were included without randomization or ‘blinding'. The survival study was performed with age- and sex-matched mice for an appropriate observation period. All mice were maintained in the specific pathogen-free facility of the Laboratory Animal Research Center at Yonsei University. Protocols were approved by the Institutional Animal Care and Use Committees of the Laboratory Animal Research Center at Yonsei University (Permit Number: IACUC-201409-212-01).

### Plasmid construction

The Flag-tagging eIF5A expression vectors (eIF5A WT and K50A) were constructed by insertion of the expression unit into the pCS4-3x Flag plasmid. The open reading frame of eIF5A complementary DNA (cDNA) was obtained from BMDMs by PCR amplification using the following primers: eIF5A forward primer 5′-CCCGAATTCAATGGCAGATGATTTGGACTTCGAG-3′ and reverse primer 5′-CCGCTCGAGTTATTTTGCCATGGCCTTGATTGCAAC-3′. The eIF5A K50A mutant form was constructed using a site-directed mutagenesis kit (Stratagene, Palo Alto, CA, USA) with the following primers: eIF5A K50A forward primer 5′-GTCTACTTCGAAGACTGGCGCGCATGGCCATGCCAAGGTCC-3′ and reverse primer 5′-GGACCTTGGCATGGCCATGCGCGCCAGTCTTCGAAGTAGAC-3′. Plasmid vector encoding iNOS mRNA was obtained from the Harvard PlasmID Database (Clone ID: MmCD00317105).

### Cell culture and *in vitro* treatment

For BMDM differentiation, primary bone marrow cells were cultured for 7 days in DMEM supplemented with 20% fetal bovine serum (FBS) (Gibco), 50 U ml^–1^ penicillin, 50 mg ml^–1^ streptomycin and 20% cultured supernatant from L929 cells. BMDMs were incubated for the indicated times in the presence of 100 ng ml^–1^ Pam3CSK4 (InvivoGen), 10 ng ml^–1^ ultrapure lipopolysaccharides (LPS) (InvivoGen), 10 μg ml^–1^ Poly (I:C) (Invivogen, HMW), 10 μg ml^–1^ CpG DNA (Invivogen, ODN1826), 10 ng ml^–1^ IFNγ (Pierce), 10 μg ml^–1^ Curdlan (Invivogen), 50 μg ml^–1^ trehalose 6,6'-dimycolate (TDM) (Sigma) or 50 μg ml^–1^ trehalose 6,6'-dibehenate (TDB) (Avanti Polar Lipids). Then, stimulations were carried out. ATP (5 mM) and monosodium urate (MSU) (300 μg ml^–1^) were from Sigma. Nigericin (0.4 μM) was from InvivoGen. ATP stimulations were performed for 1 h, other stimulations for 3 h. For the stimulation of the AIM2 inflammasome, poly(dA:dT) (purchased from InvivoGen) was admixed at the indicated concentrations to Lipofectamine 2000 (from Invitrogen) according to the manufacturer's instructions, and cells were stimulated for 3 h. For inhibitor assay, indicated inhibitors were pretreated for 30 m before stimulation. PP1 (5 μM, Src inhibitor, 529579), Syk inhibitor (10 μM, 574711), BAPTA (5 μM, 196419), W-7 (10 μM, 681629), AG17 (10 μM, 658425), SB203580 (10 μM, 559389) were from CalBiochem. BHA (10 μM, B1253), Strychnine (1 μM, 50532), Y-27632 (10 μM, Y0503), H-7 (300 μM, I7016), AG490 (25 μM, T3434), L-NMMA (100 μM, M7033), 1400W (10 μM, W4262), NOC-18 (10 μM, A5581), SP600125 (10 μM, S5567), PD169316 (10 μM, P9248) and ciclopirox (20 μM, C0415) were from Sigma. Parthenolide (5 μM, 0610), U0126 (10 μM, 1144) and Wortmannin (10 μM, 1232) were from Tocris. z-VAD-FMK (50 μM, 550377) was from BD Bioscience. GC7 (125 μM, 259545) was from EMD Millipore.

### ELISA and immunoblot analysis

TNFα and IL-1β (BioLegend) in culture supernatants were measured by ELISA. For immunoblot analysis, cells and tissues were lysed for 10 m at 4 °C in RIPA buffer (100 mM Tris–HCl (pH 8.0), 50 mM NaCl, 5 mM EDTA, 0.5% NP-40, 1% Triton X-100, 50 mM β-glycerophosphate, 50 mM NaF, 0.1 mM Na_3_VO_4_, 0.5% sodium deoxycholate, with a protease inhibitor ‘cocktail' (1 mM PMSF, 10 μg ml^–1^ aprotinin, 5 μg ml^–1^ pepstatin and 5 μg ml^–1^ leupeptin), followed by centrifugation at 13,000*g* for 10 m at 4 °C for the removal of debris. Proteins from the cell culture supernatants were precipitated by methanol–chloroform extraction. Equal amounts of proteins were analysed by immunoblot, and signals developed with Amersham ECL reagents were detected with the ImageQuant LAS 4000 system (GE Healthcare). Full blottings are shown in Supplementary Fig. 20. Image J was used for densitometry calculation^60^. Antibody information is provided in Supplementary Table 2. We used all antibodies in 1:1,000 dilution condition for immunoblot analysis.

### Detection of *S*-nitrosylated proteins

The biotin-switch assay for the detection of *S*-nitrosylated proteins was performed using an *S*-nitrosylated protein detection assay kit (10006518, Cayman), as recommended by the manufacturer. Biotinylated proteins were immunoprecipitated by streptavidin-sepharose (GE Healthcare) for 30 m at room temperature. Bound proteins were visualized by immunoblot analysis.

### Polysome analysis

The translation efficiency of mRNA was analysed by measurement of the relative distribution of mRNA in a 10–50% sucrose density gradient polysomal fractions, as previously described^61^. In this study, BMDMs (4.5 × 10^7 ^cells) treated for 12 h with indicated stimulation. Washed cells were suspended for 10 m in 500 μl polysome lysis buffer (5 mM Tris–HCl (pH 7.5), 2.5 mM MgCl_2_, 1.5 mM KCl, 200 μg ml^–1^ cycloheximide, 1,000 U ml^–1^ RNAsin (Promega), 2 mM dithiothreitol, 0.5% Triton X-100, and the protease inhibitor ‘cocktail' described above) and then centrifuged at 13,000*g* for 10 m at 4 °C. The collected supernatants were then loaded onto a sucrose gradient prepared in 20 mM HEPES–KOH (pH 7.5), 100 mM KCl, and 5 mM MgCl_2_ and centrifuged at 35,000*g* for 2 h at 4 °C with an SW41 Ti rotor. Fractions were collected by piercing the tube with a Brandel tube piercer, passing 60% sucrose through the bottom of the tube, and monitoring the absorbance at 254 nm (UV flow cell of Äkta Prime; GE Healthcare) to obtain the polysome profiles. Immunoblot analyses used equal volumes of samples from each fraction. RNA was isolated from individual fractions using the TRIzol reagent (Invitrogen) and quantitated by qRT-PCR with gene-specific primers.

### RNA analysis

Total RNA from tissues and cells was isolated with the TRIzol reagent (Invitrogen) according to the manufacturer's instructions. Then, cDNA was synthesized by SuperScript II reverse transcriptase (Invitrogen) with random hexamers or oligo-dT as the primers. The expression of individual genes was measured by real-time PCR using a Bio-Rad CFX, and was quantitatively normalized to the housekeeping gene *Gapdh* by the change-in-cycling-threshold (ΔΔ*C*_T_) method (primers, Supplementary Table 3).

### Measurement of eIF5A hypusination

The measurement of eIF5A^Hyp^ level was described previously^62^. In this study, BMDMs (1.5 × 10^6^ cells) were stimulated with the indicated treatment for 16 h and co-cultured with 1.5 μCi ^3^H-Spermidine (Perkin Elmer) in the presence of 1 mM aminoguanidine, an inhibitor of amine oxidase in serum. Then, cells were lysed with 2 × Laemmli buffer and proteins were loaded for electrophoresis on a 12% SDS-polyacrylamide gel. The radioactivity incorporated into eIF5A was determined by fluorography. The gel was destained with deionized water for 15 min and exposed to X-Ray film (BioMax XAR film) at −70 °C for more than 20 days.

### Cross-linked RIP

NIH-3T3 cells were co-transfected with iNOS-mRNA-plasmid along with Flag-Mock or Flag-eIF5A(WT) or Flag-eIF5A(K50A), and trypsinized after 48 h. Then, cells were fixed with 3% formaldehyde in PBS for 10 m. And glycine was added for blocking the formaldehyde. After washing the cells twice with cold PBS, the cells were resuspended in 500 μl of IP lysis buffer (50 mM Hepes (pH7.5), 0.4 M NaCl, 1 mM EDTA, 1 mM DTT, 0.5% TritonX-100, 10% Glycerol) and lysed by sonication (10 pulses for 10 s). And then, Flag-tagged protein in the lysate was immunoprecipitated using the anti-Flag M2 Affinity Gel (Sigma-Aldrich) overnight at 4 °C. After collection by centrifugation at 2,000 g and washing 3–4 times with IP lysis buffer, the complexes were reverse cross-linked by adding RIP buffer (50 mM Hepes (pH7.5), 0.1 M NaCl, 5 mM EDTA, 10 mM DTT, 0.5% Triton X-100, 10% glycerol, 1% SDS in diethylpyrocarbonate-treated water). RNA was extracted using Trizol reagent (Invitrogen) and iNOS mRNA was quantitated by qRT-PCR.

### Administration of TDM

For the induction of pulmonary granulomas through the administration of TDM, TDM (Sigma) was dissolved with choloroform:methanol (9:1) solution to make 1 mg ml^–1^ concentration. For 20 μg TDM injection per a mouse, solubilized 100 μl of 1 mg ml^–1^ TDM was transferred into a Potter–Elvehjem style glass pestle, and the solvent was evaporated under a stream of nitrogen gas. And then, a TDM-oil-in-water emulsion was prepared by grinding the dry TDM into 9 μl of mineral oil, then homogenizing with a 1 μl Tween-80 in 90 μl PBS vehicle (oil-in-water emulsion consisting of mineral oil (9%), Tween-80 (1%) and PBS (90%)). Additional sonication of the emulsion was helpful to make granuloma formation. And then, 100 μl of water-in-oil emulsion containing 20 μg of TDM was injected intravenously into an 8–11-week-old mouse. For co-injection of GC7 with TDM or CPX with TDM, we daily intraperitoneally injected GC7 at a dose of 4 mg kg^–1^ or CPX at a dose of 50 mg kg^–1^ mouse weight throughout the duration of the experiments. At days 7 and 14, lungs were weighed and the left lung was fixed in 10% formaldehyde for hematoxylin–eosin (H&E) staining and immunohistochemistry. Results are expressed as relative granuloma area per fixed field of view at a magnification of 10 × . Quantitation of granuloma areas from at least five randomly selected fields from each slide of three independent experiments was analysed using Adobe Photoshop software (Adobe Systems). For the analysis of cellular composition profile, the right lungs were recovered, weighed, incubated in 2 mg ml^–1^ collagenase D (Roche) and 40 U ml^–1^ DNase I (Roche) solution, and dispersed by passage through a 70-μm mesh. After lysis of red blood cells, viable cells were counted. Subsequently, cells were incubated with fluorescence-conjugated antibodies, and the flow cytometry assay was performed as previously described^63^. Antibodies (BD Pharmingen) used were against Gr-1 (RB6-8C5; 1:100), CD11b (M1/70; 1:100), Ly6G (1A8; 1:100), CD3ɛ (145-2C11; 1:100) and CD19 (1D3; 1:100). In some experiments, the remaining right upper lobes were conserved with ice-cold saline containing 1 mM PMSF, and then snap-frozen and stored at −70 °C for ELISA and immunoblot.

### RNA sequencing and data analysis

RNAs from cell lysates or polysome fractions were purified with RNeasy mini kit (Qiagen), including a DNase digestion, and were converted to cDNA libraries according to the Illumina TruSeq RNA SamplePrep Guide. We obtained polysome-seq data from sequencing of two biological replicates. Total lysate-seq and polysome-seq were performed with Illumina HiSeq-2000 platform at KRIBB in Korea. Then, we used Tuxedo tools for processing RNA-seq data, introduced in Trapnell *et al.*^64^. The RNA-seq data were mapped to the genome mm10 using TopHat (version 2.0.10), which includes Bowtie as a short-read aligner. Mapped reads (alignments) were assembled on the genome by Cufflinks (version 2.1.1). Cufflinks calculates the relative abundance between transcripts. When assembling transcripts, we used the NCBI mRNA reference sequence collection (RefSeq). Subsequently, the sample set was normalized by Cuffnorm (version 2.2.1) to minimize inter-sample variation and compare fragments per kilobase of transcript per million mapped reads values of all samples from different experimental conditions. The GEO accession number for the RNA sequencing data reported in this paper is GSE70793. Furthermore, we used the gene ontology analysis programme in innateDB (www.innatedb.com), an online-based analysis system. Gene lists used in ontology analysis were annotated by RefSeq ID. We chose hypergeometric analysis as ontology analysis algorithm and the Benjamini–Hochberg method with *P* value correction.

### Statistical analysis

Statistical analysis was performed with Prism 6.0 software (GraphPad). An unpaired two-tailed *t-*test with 95% confidence interval was used for calculation of *P* values. For survival experiments, a log-rank (Mantel–Cox) test was used for calculation of *P* values. Group sizes, reproducibility and *P* values for each experiment are given in figure legends.

## Additional information

**How to cite this article:** Lee, W.-B. *et al.* Mincle-mediated translational regulation is required for strong nitric oxide production and inflammation resolution. *Nat. Commun.* 7:11322 doi: 10.1038/ncomms11322 (2016).

## Supplementary Material

Supplementary Information Supplementary Figures 1-20 and Supplementary Tables 1-3
