# Supplementary material for: Mincle-mediated translational regulation is required for strong nitric oxide production and inflammation resolution
Source: Nat Commun. 2016 Apr 18;7:11322. doi: 10.1038/ncomms11322 (PMC4837483; doi:10.1038/ncomms11322)
Supplement: Supplementary Information — Supplementary Figures 1-20 and Supplementary Tables 1-3 [file ncomms11322-s1.pdf]

Supplementary Figure 1

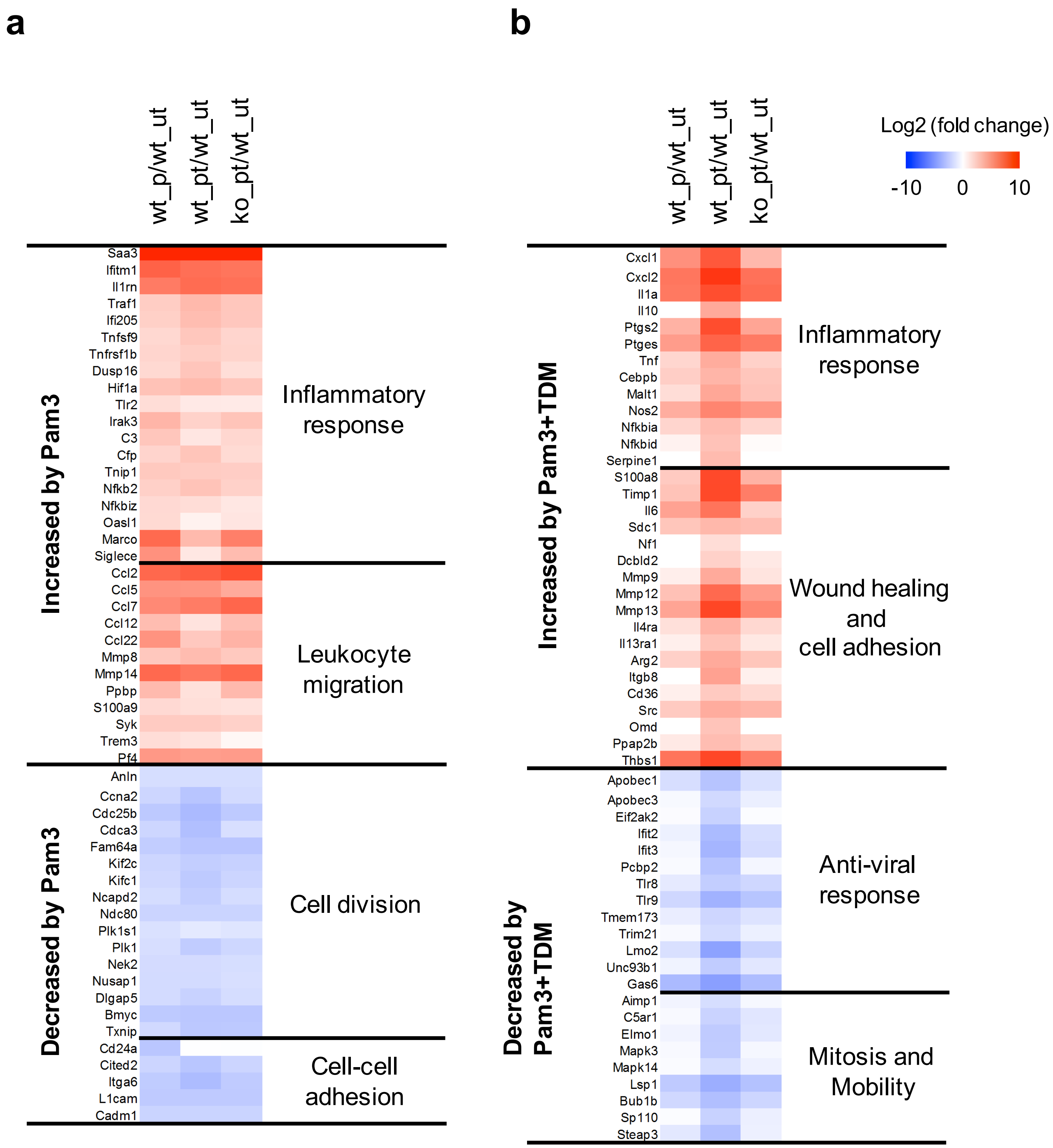

Supplementary Figure 1. RNA-seq analysis shows a strong immune signal by Mincle and TLR2 co-stimulation. Wild-type (wt) and Mincle$^{-/-}$ (ko) BMDMs were stimulated with Pam3 (p) or co-stimulated with Pam3 and TDM (pt) for 12 h, and then performed RNA-sequencing. Heatmap showing log2 expression ratio to untreated (ut). wt_p/wt_ut; comparing Pam3 treated to untreated in wt, wt_pt/wt_ut; comparing Pam3 and TDM co-treated to untreated in wt, ko_pt/wt_ut; comparing Pam3 and TDM co-treated in ko to untreated in wt. (**a**) Heatmap distribution showing the gene expression changes induced by Pam3. For showing the genes only influenced by Pam3, first we selected genes up- or down-regulated by Pam3 ((wt_p/wt_ut) > 2 fold or (wt_p/wt_ut) < 0.5 fold). And then, we discard TDM influenced genes (0.5 fold < {(wt_pt/wt_ut)/(wt_p/wt_ut)} < 2 fold). (**b**) Heatmap distribution showing the gene expression changes induced by Pam3 and TDM co-stimulation. For showing the genes influenced by TDM, first we selected genes up- or down-regulated by Pam3 and TDM co-treatment ((wt_pt/wt_ut) > 2 fold or (wt_pt/wt_ut) < 0.5 fold). And then, we chose TDM influenced genes ({(wt_pt/wt_ut)/(wt_p/wt_ut)} > 2 fold or {(wt_pt/wt_ut)/(wt_p/wt_ut)} < 0.5 fold).

Supplementary Figure 2

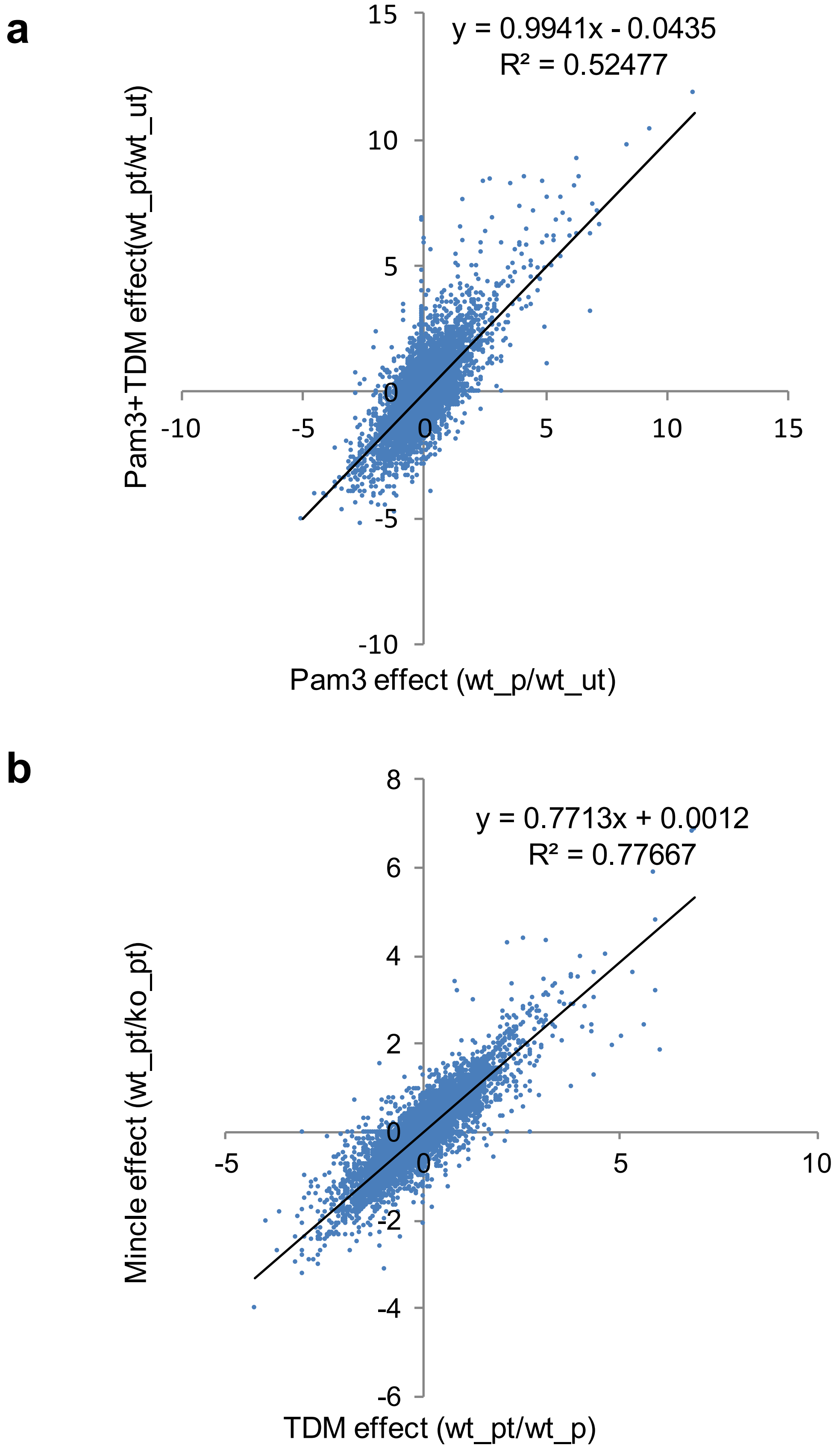

Supplementary Figure 2. TDM-stimulated transcriptional changes are dependent on Mincle. The scatter plots represent RNA-seq data from **Supplementary Fig. 1**. (**a**) Scatter plot between the transcription profile of Pam3-treated cells (wt_p/wt_ut) and Pam3-TDM co-treated cells (wt_pt/wt_ut). (**b**) Scatter plot between the transcription profile of TDM effect (wt_pt/wt_p) and Mincle effect (wt_pt/ko_pt). Genes that had low expression level (FPKM<3) were substituted with 3 FPKM value to filter out insignificant expression level change.

Supplementary Figure 3

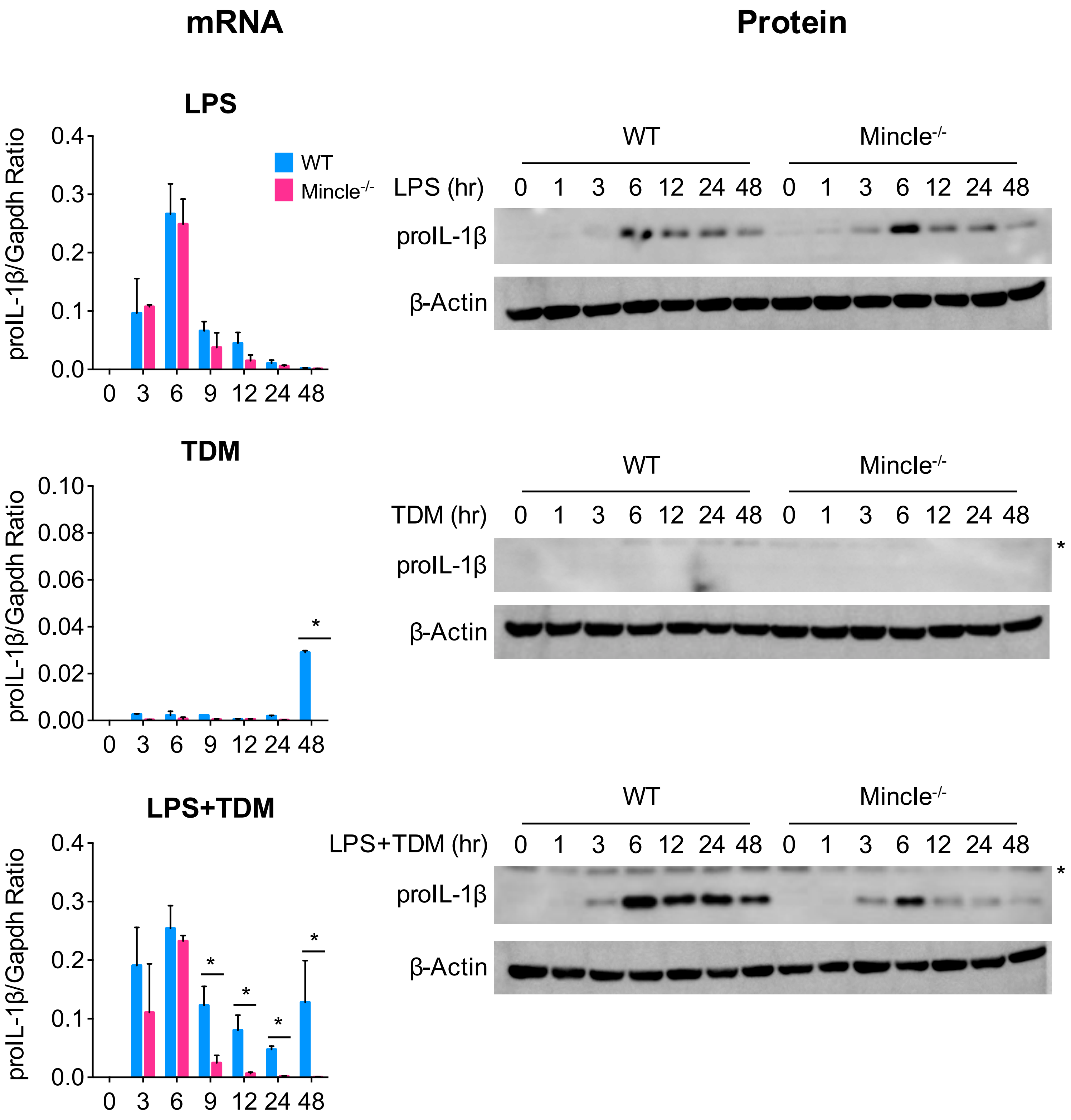

Supplementary Figure 3. TDM alone fails to induce proIL-1β expression WT and Mincle$^{-/-}$ BMDMs were stimulated with LPS, TDM, or co-stimulated with LPS and TDM for indicated time. Left; qRT-PCR analysis of proIL-1β mRNA levels from each stimulated macrophages. *$p < 0.05$ (Student's *t*-test). Right; Immunoblot analysis of proIL-1β protein expressions from each stimulated macrophages. Data are representative of two independent experiments (mean and s.d.). The asterisk represents a nonspecific band.

Supplementary Figure 4

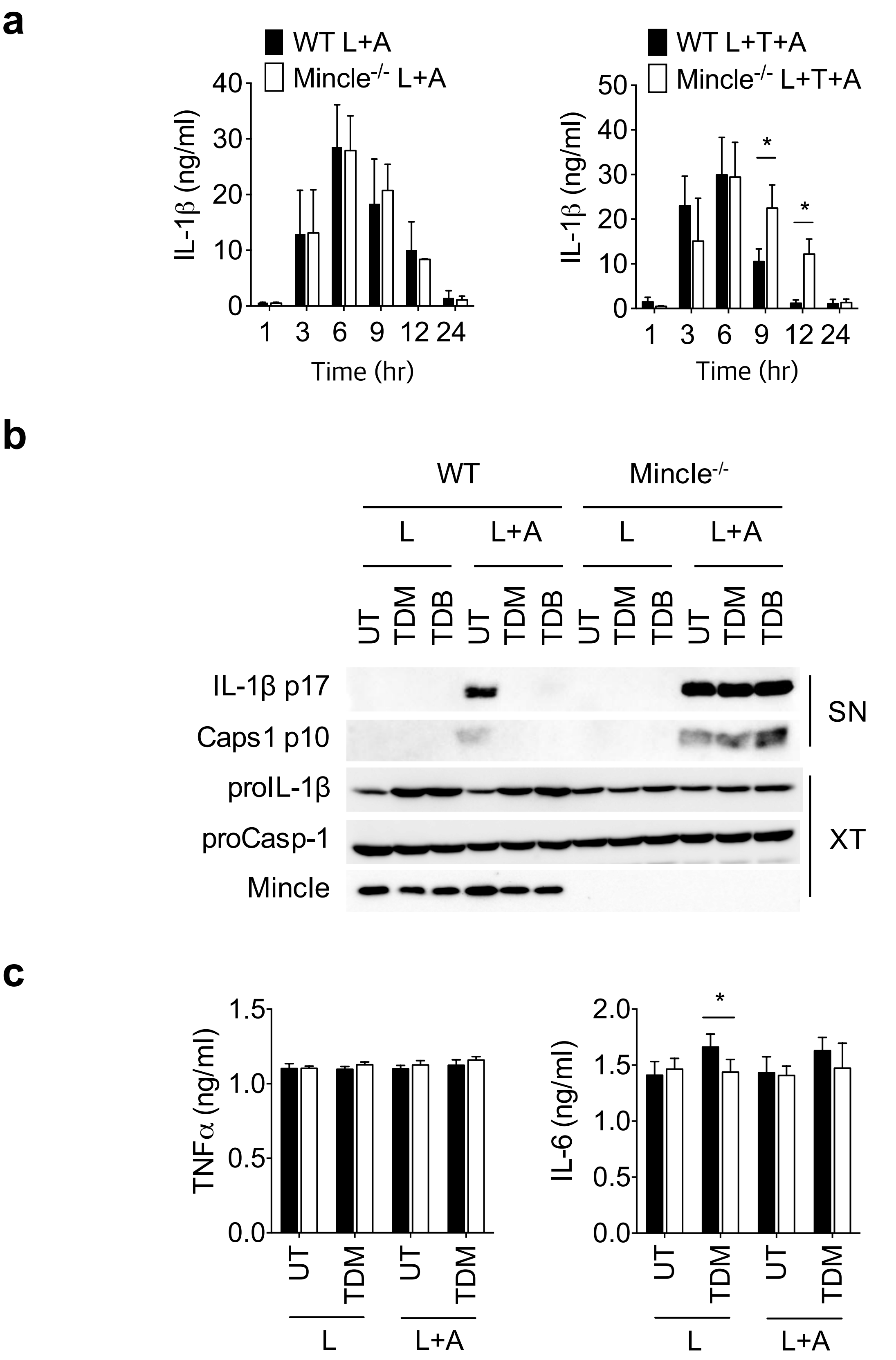

Supplementary Figure 4. Late phase of LPS and TDM co-stimulation shows the reduction of caspase-1 activation and IL-1β secretion WT and Mincle$^{-/-}$ BMDMs were stimulated with LPS or co-stimulated with LPS and TDM/TDB for 12 h or indicated time, and then treated with ATP for 1 h (L;LPS, T;TDM, A; ATP, UT;untreated). (**a**) ELISA of secreted IL-1β. (**b**) Immunoblot analysis of IL-1β and Caspase-1. (**c**) ELISA of TNFα or IL-6. *$p < 0.05$(two-tailed unpaired t-test). Data are representative of two (**b**) or three (**a,c**) independent experiments. (**a,c**; mean and s.d.)

Supplementary Figure 5

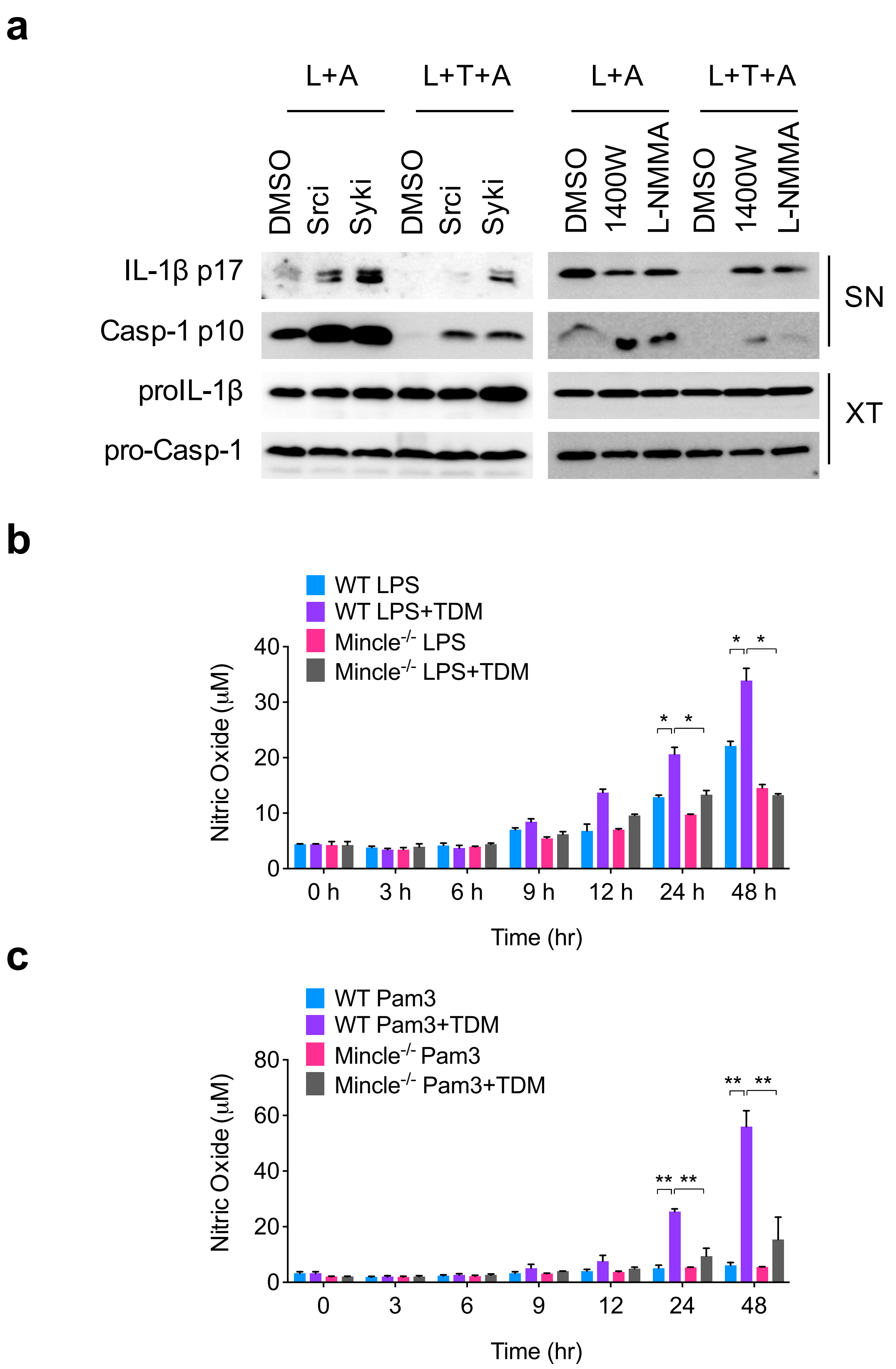

Supplementary Figure 5. NO production is synergistically increased by co-stimulation of Pam3 and TDM (**a**) Immunoblot analysis of processing of IL-1β and caspase-1 from WT BMDMs stimulated with LPS (L+A) or co-stimulated with LPS and TDM (L+T+A) for 12 h in the presence of indicated chemical inhibitors, and then treated with ATP for 1 h. Cell culture supernatants (SN) and whole-cell lysates (XT) were analyzed. (**b-c**) Nitric oxide production from cell culture supernatant of WT and Mincle$^{-/-}$ BMDMs (**b**) treated with LPS or co-treated with LPS and TDM, (**c**) treated with Pam3 or co-treated with Pam3 and TDM. *p<0.05, **p<0.001 (two-tailed unpaired t-test). Data are representative at least three independent experiments. (mean and s.d.)

Supplementary Figure 6

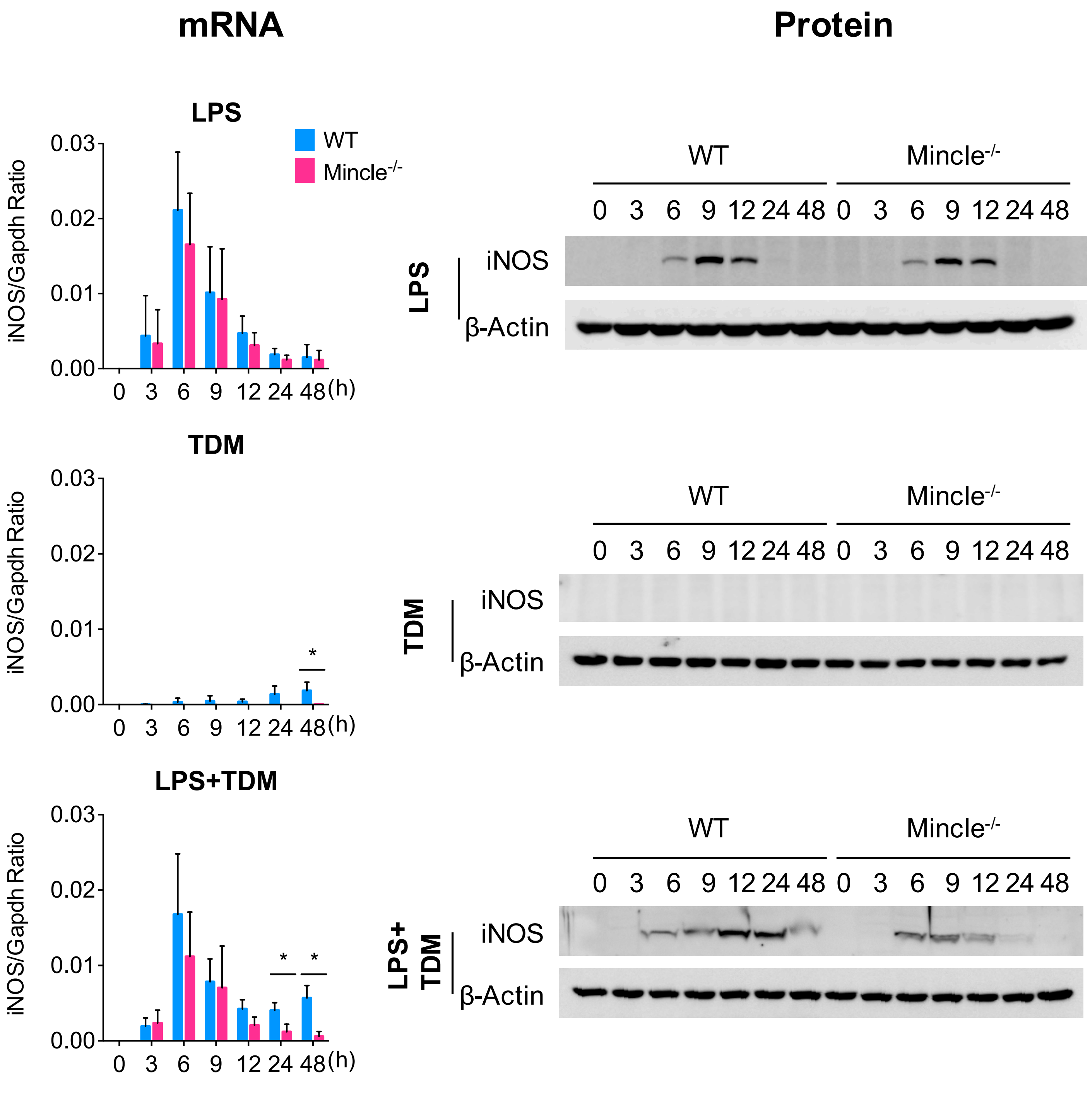

Supplementary Figure 6. TDM-induced iNOS expression is related on translational regulation WT and Mincle$^{-/-}$ BMDMs were stimulated with LPS, TDM, or co-stimulated with LPS and TDM for indicated time. Left; qRT-PCR analysis of iNOS mRNA levels from each stimulated macrophages. *$p < 0.05$ (Student's *t*-test). Right; Immunoblot analysis of iNOS protein expression from each stimulated macrophages. Data are representative of three independent experiments (mean and s.d.).

Supplementary Figure 7

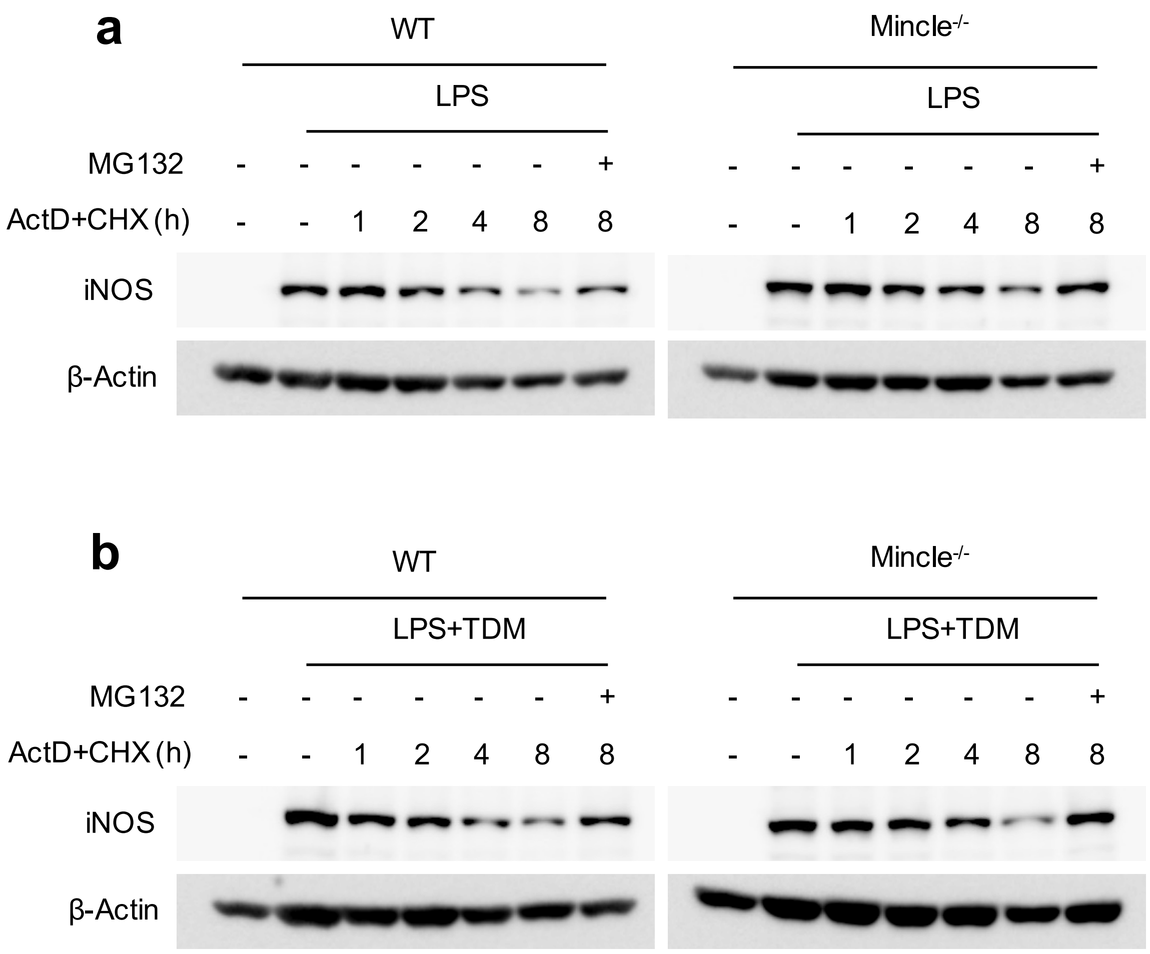

Supplementary Figure 7. iNOS protein stabilities are independent from Mincle signaling The half-life of the iNOS protein was measured after inhibiting both mRNA transcription and protein translation with Actinomycin D (ActD, 10 µg/ml) and cycloheximide (CHX, 100 µg/ml). WT and Mincle$^{-/-}$ BMDMs were stimulated with LPS or co-stimulated with LPS and TDM for 6 h and directly harvested, or stimulated cells were additionally treated with ActD and CHX for the indicated times (h) before harvest. For negative control of protein degradation, ActD and CHX treated cells are additionally treated with MG132 (10 µM). Macrophages were lysed and immunoblotted with indicated antibodies. β-Actin level is shown as a loading control. The band intensities were quantified using Multi Gauge 2.2 (Fuji Film).

Supplementary Figure 8

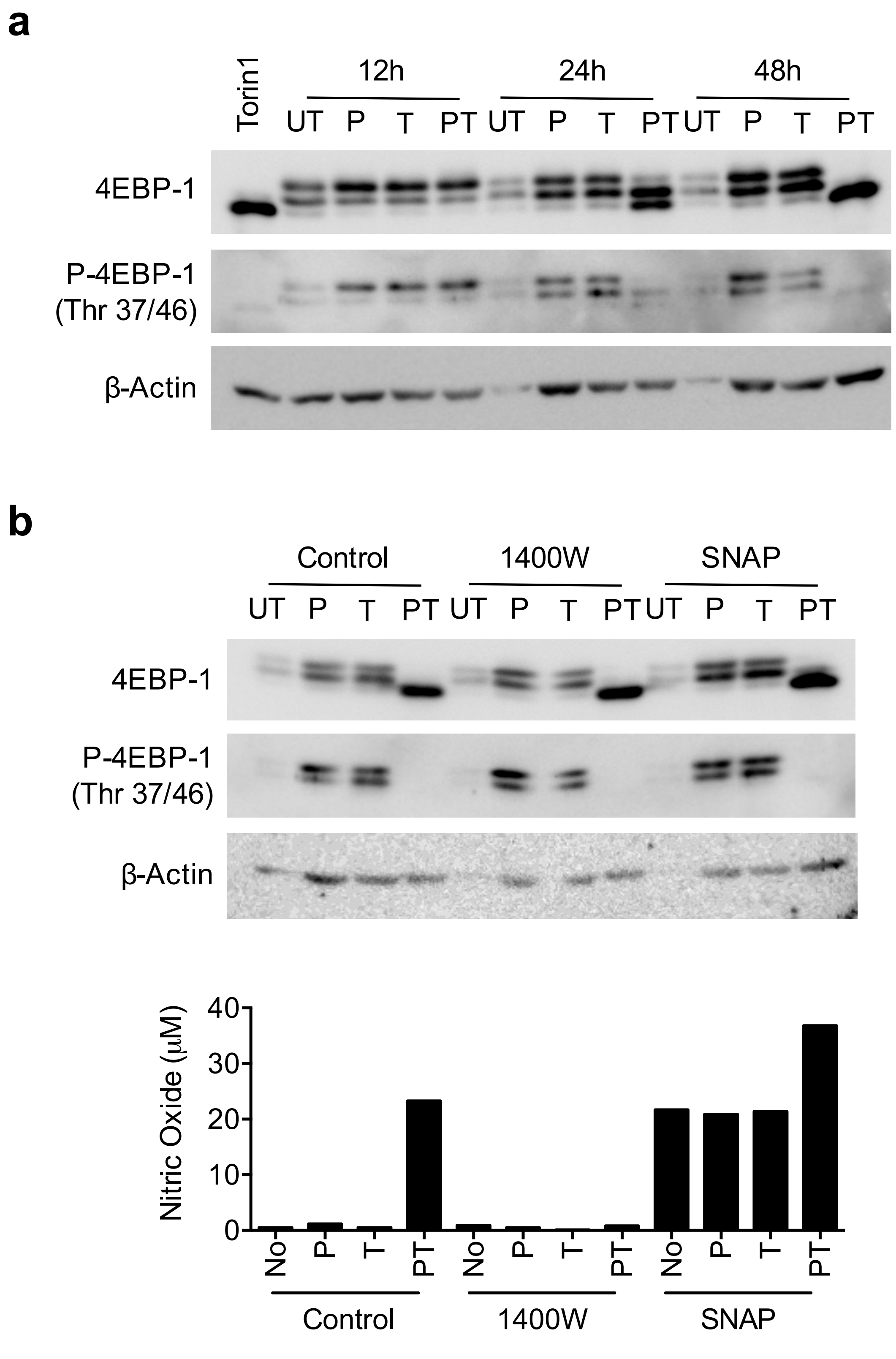

Supplementary Figure 8. Dephosphorylation of 4EBP-1 in a Mincle-dependent manner (**a**) Immunoblot analysis of 4EBP-1 and specific phosphor-4EBP-1 (Thr 37/46) from macrophages treated with Torin1, Pam3 (P), TDB (T), or co-treated with Pam3 and TDB (PT) for indicated time. UT; untreated. (**b**) Top; Immunoblot analysis of 4EBP-1 and specific phosphor-4EBP-1 (Thr 37/46) from macrophages pre-treated with 100 μM 1400W or 100 μM SNAP, and then treated with Pam3 (P), TDB (T), or co-treated with Pam3 and TDB (PT) for 24 h. UT; untreated. Bottom; Nitric oxide production in the indicated sample culture supernatants.

Supplementary Figure 9

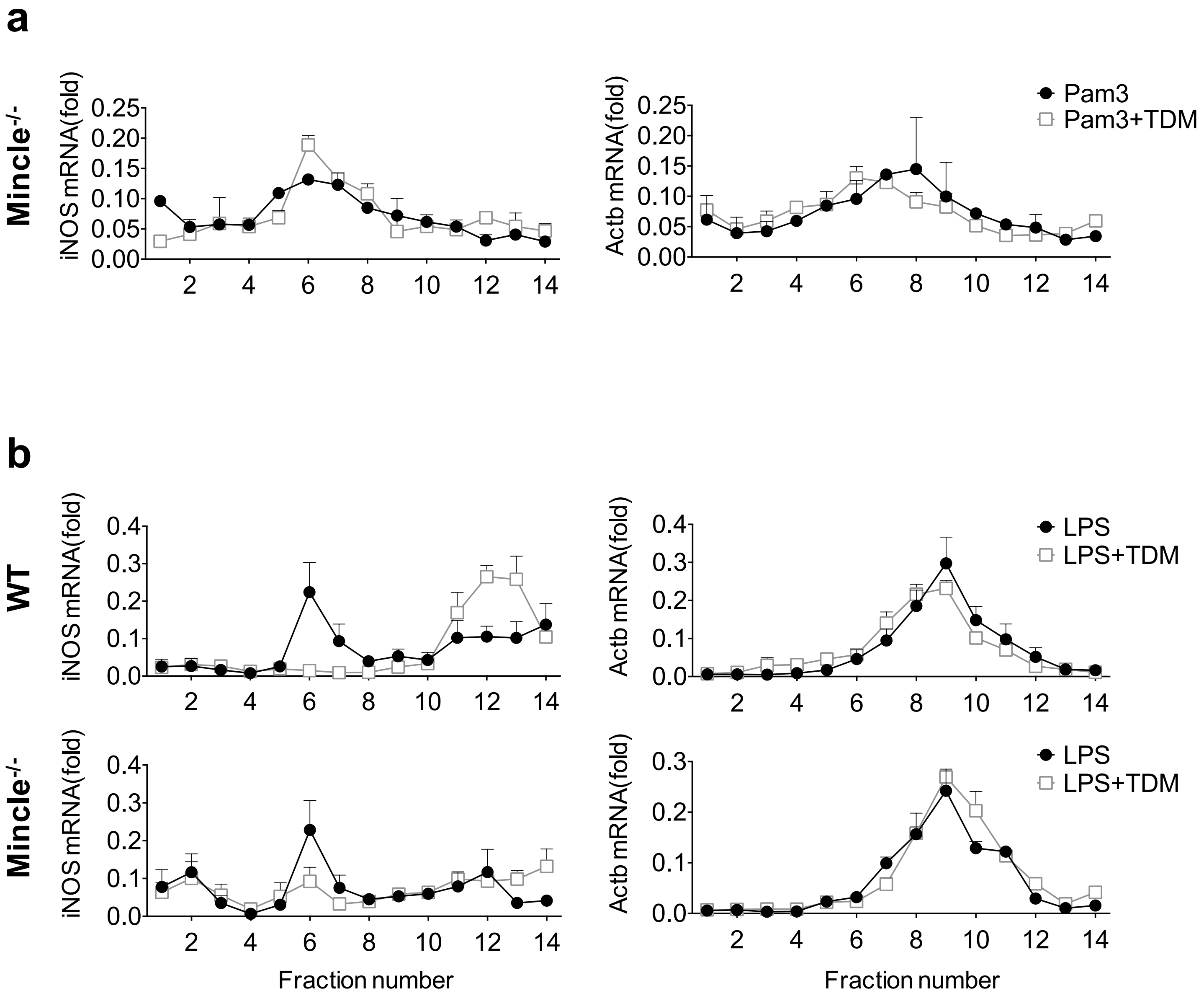

Supplementary Figure 9. The distribution pattern of individual mRNAs along the polysome analysis fractions (**a-b**) qRT-PCR analysis of iNOS (left) and β-Actin (right) mRNA in polysome fractions from WT and Mincle$^{-/-}$ macrophages (**a**) stimulated with Pam3 or co-stimulated with Pam3 and TDM, or (**b**) stimulated with LPS or co-stimulated with LPS and TDM, presented for each fraction relative to the sum of all 14 fractions. Data are representative of at least three independent experiments

Supplementary Figure 10

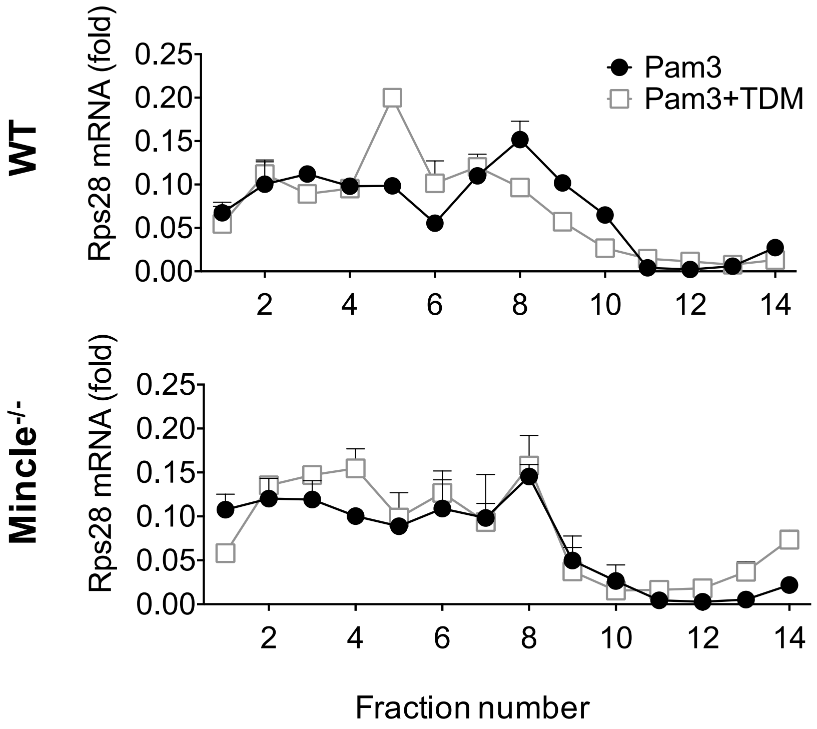

Supplementary Figure 10. The distribution pattern of Rps28 mRNAs along the polysome analysis fractions qRT-PCR analysis of Rps28 mRNA in polysome fractions from WT and Mincle $^{-/-}$ macrophages stimulated with Pam3 or co-stimulated with Pam3 and TDM, presented for each fraction relative to the sum of all 14 fractions. Data are representative of at least three independent experiments.

Supplementary Figure 11

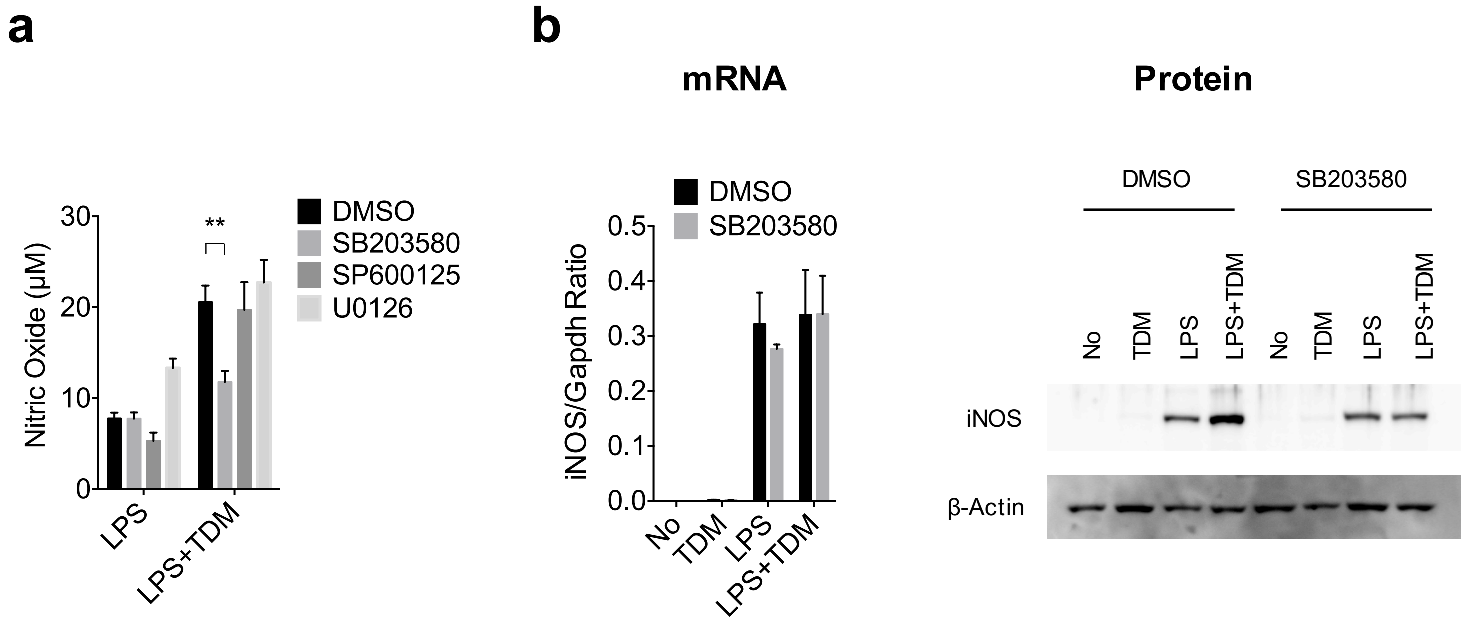

Supplementary Figure 11. p38 activation is required for Mincle-induced iNOS translation (a) Nitric oxide production in the culture supernatants from LPS-treated WT BMDMs, stimulated with TDM in the presence of indicated chemical inhibitors for 12 h. **p < 0.01 (Student's *t*-test). (**b**) WT BMDMs were stimulated with LPS, TDM, or co-stimulated with LPS and TDM in the presence of DMSO or SB203580 for 12 h. qRT-PCR analysis of iNOS mRNA levels (left) and immunoblot analysis of iNOS protein expression (right) from each stimulated macrophages. Data are representative of at least three independent experiments (**a**,**b**; mean and s.d.).

Supplementary Figure 12

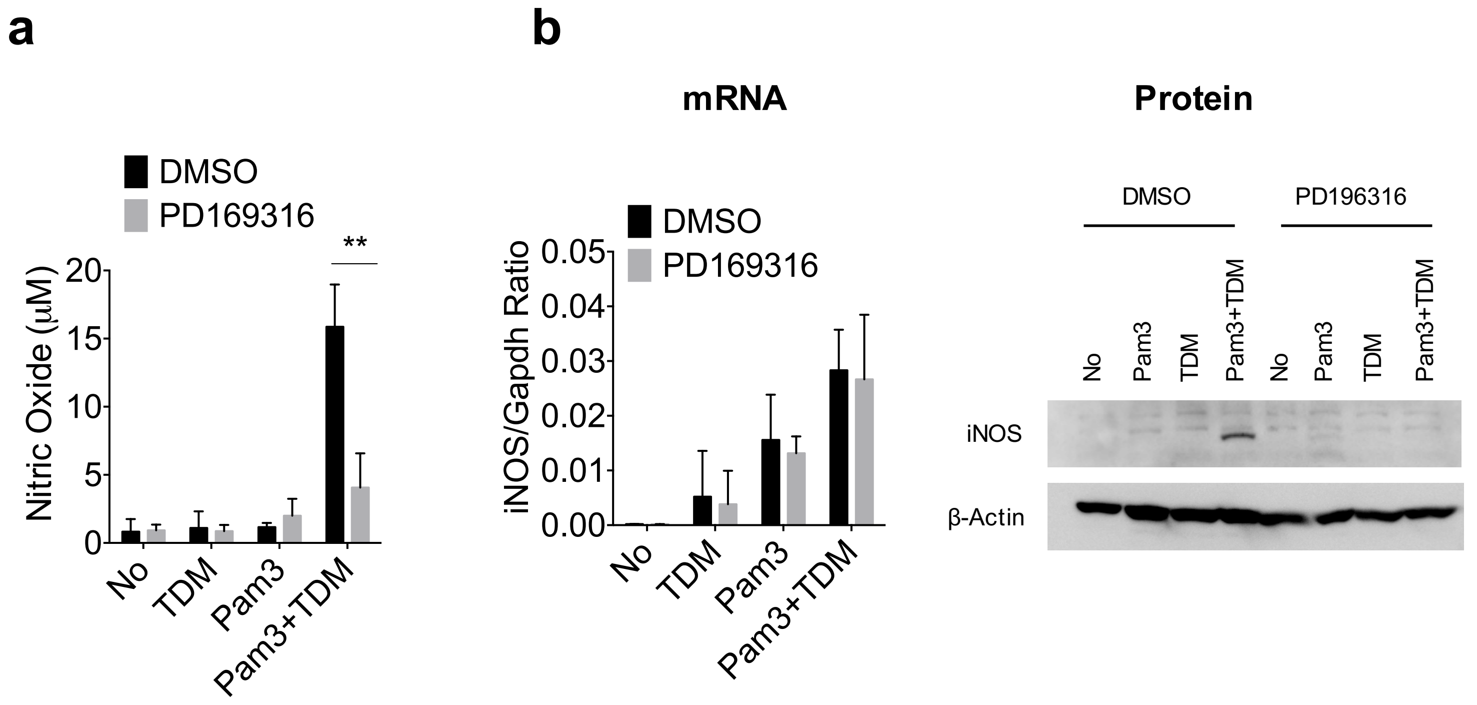

Supplementary Figure 12. PD169316, a p38 inhibitor, blocks TDM-induced iNOS translation WT BMDMs were stimulated with TDM, Pam3, or co-stimulated with Pam3 and TDM in the presence of PD169316, for 12 h. (**a**) Nitric oxide production in culture supernatants. **p < 0.01 (Student's *t*-test). (**b**) left: qRT-PCR analysis of iNOS mRNA levels from stimulated macrophages; right: Immunoblot analysis of iNOS protein expression. Data are representative of at least three independent experiments (**a-b**; mean and s.d.).

Supplementary Figure 13

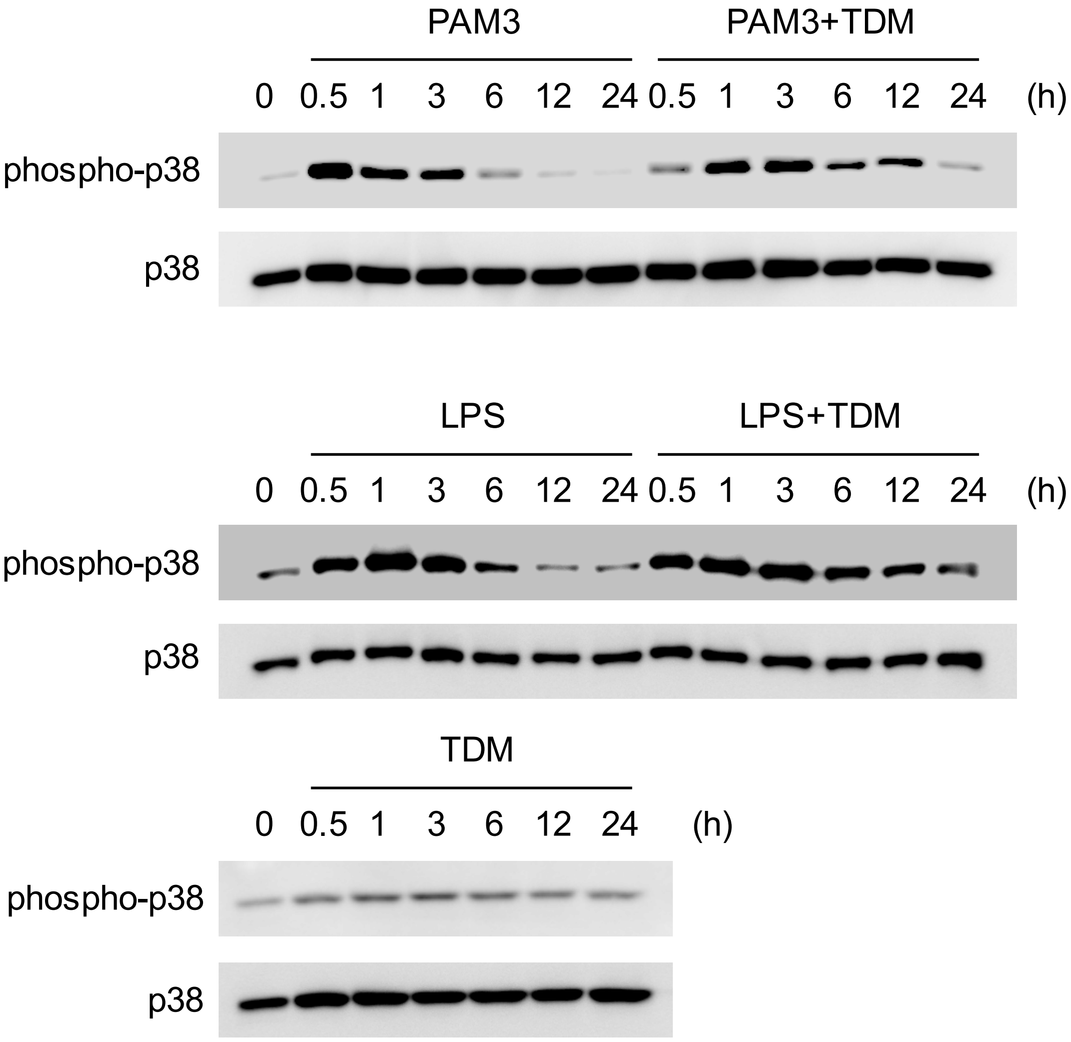

Supplementary Figure 13. Phosphorylation of p38 is extended by Mincle Immunoblot analysis of phospho-p38 from WT BMDMs stimulated with Pam3, LPS, or co-stimulated each with TDM for indicated time. Data are representative of at least three independent experiments

Supplementary Figure 14

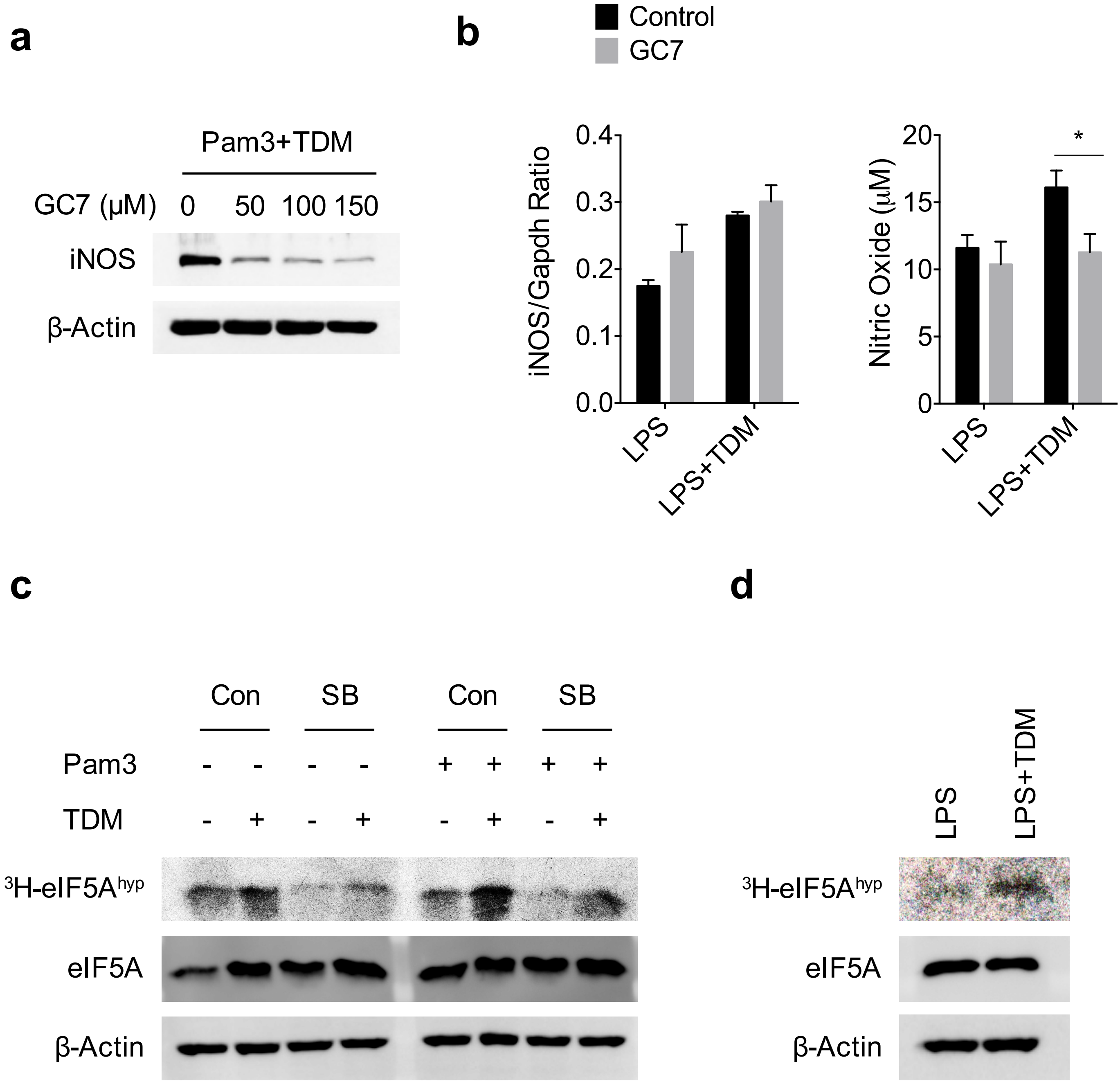

Supplementary Figure 14. Mincle controls eIF5A hypusination for iNOS translation (**a**) Immunoblot analysis of iNOS from WT BMDMs co-stimulated with Pam3 and TDM in the presence of indicated concentration of GC7. (**b**) WT BMDMs were stimulated with LPS or co-stimulated with LPS and TDM in the presence of GC7 for 12 h. left: Nitric oxide release in the culture supernatants; right: qRT-PCR analysis of iNOS mRNA levels. (**c**) Fluorographic analysis of hypusinated eIF5A from WT BMDMs treated with Pam3, TDM, or co-treatment of Pam3 and TDM in the presence of control (con) or SB203580 (SB). (**d**) Fluorographic analysis of hypusinated eIF5A from WT BMDMs treated with LPS or co-treatment of LPS and TDM. $^{*}p<0.05$ (Student's *t*-test). Data are representative of three (**b**) or two (**a**,**c**,**d**) independent experiments (**b**; mean and s.d.).

Supplementary Figure 15

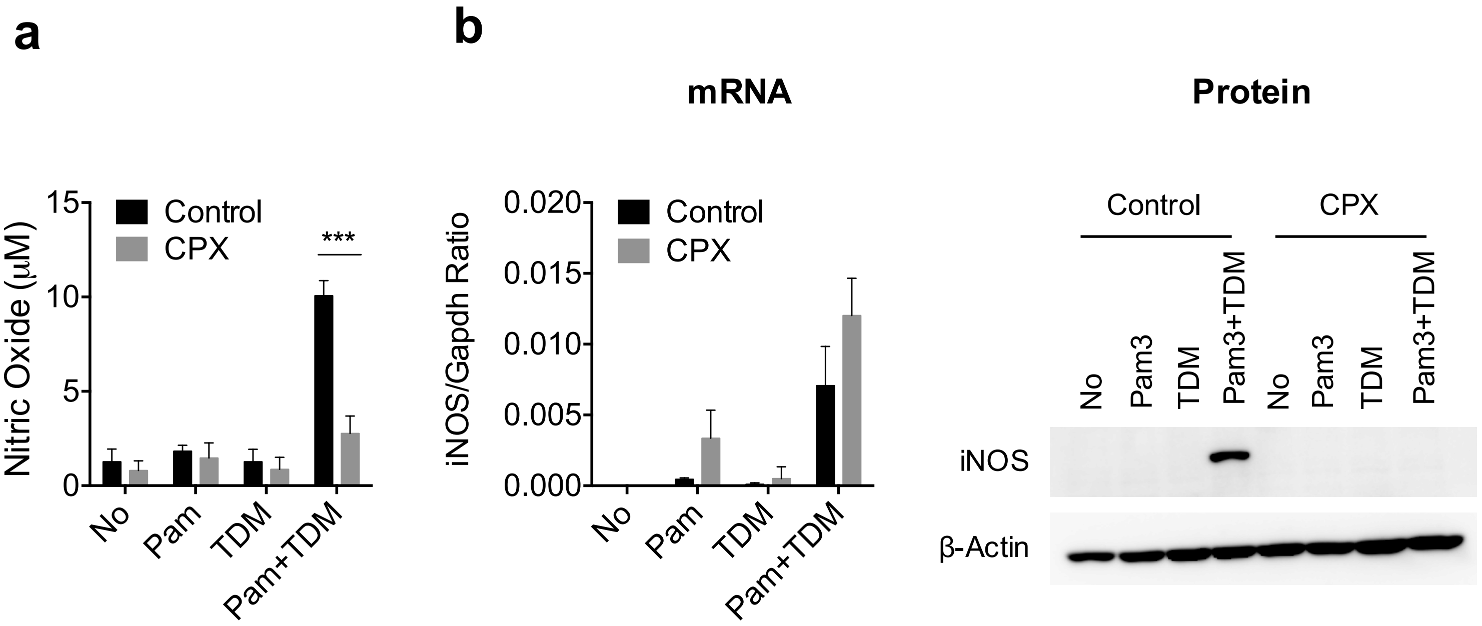

Supplementary Figure 15. CPX, a DOHH inhibitor, selectively suppresses translation of iNOS WT BMDMs were stimulated with TDM, Pam3, or co-stimulated with Pam3 and TDM in the presence of 20 μm CPX, for 12 h. (**a**) Nitric oxide production in culture supernatants. ***p < 0.001 (Student's *t*-test). (**b**) left: qRT-PCR analysis of iNOS mRNA levels from stimulated macrophages; right: Immunoblot analysis of iNOS protein expression. Data are representative of at least three independent experiments (**a-b**; mean and s.d.).

Supplementary Figure 16

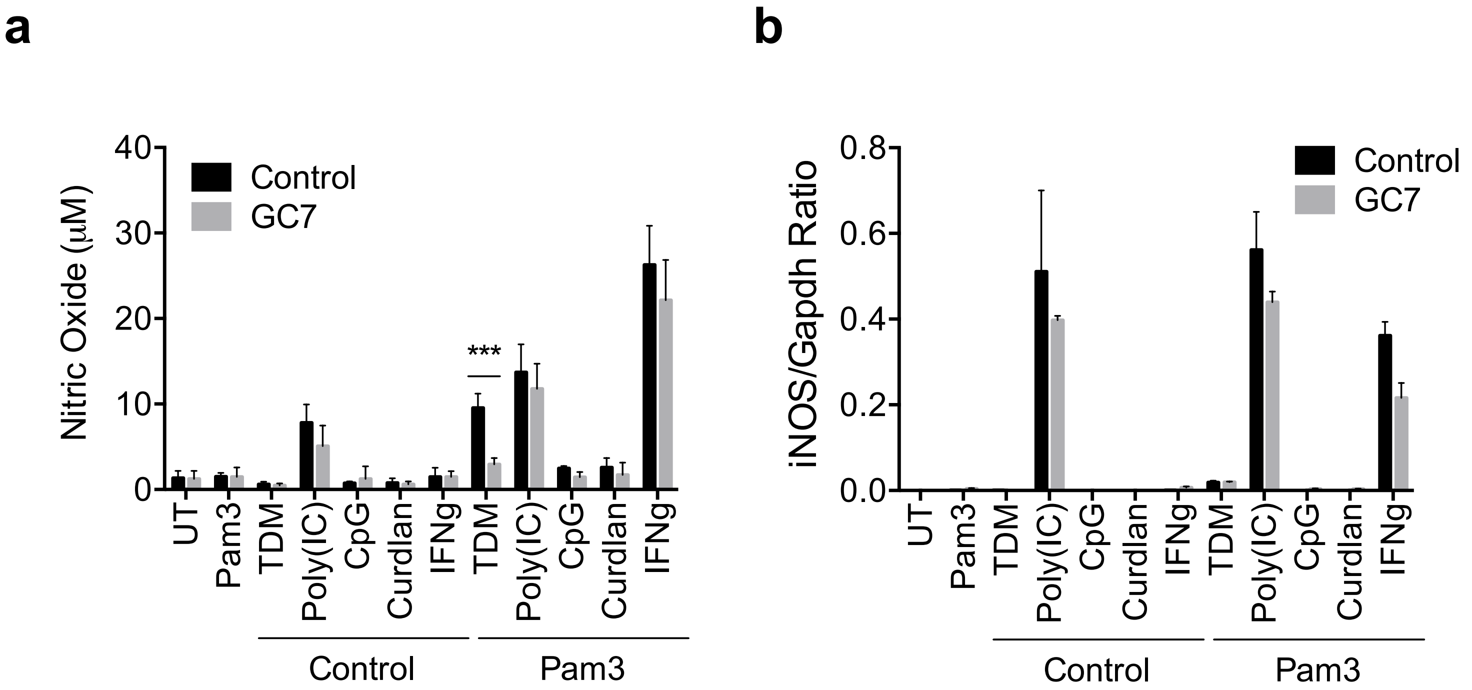

Supplementary Figure 16. Mincle-eIF5A^{hyp} mediated translational regulation is a unique mechanism for iNOS synthesis WT BMDMs were stimulated with TDM, Poly(I:C), CpG DNA, Curdlan, IFNγ or co-stimulated Pam3 with each of them for 12 h. (**a**) Nitric oxide production in culture supernatants. ***p < 0.001 (Student's *t*-test). (**b**) qRT-PCR analysis of iNOS mRNA levels from stimulated macrophages. Data are representative of at least three independent experiments (**a-b**; mean and s.d.).

Supplementary Figure 17

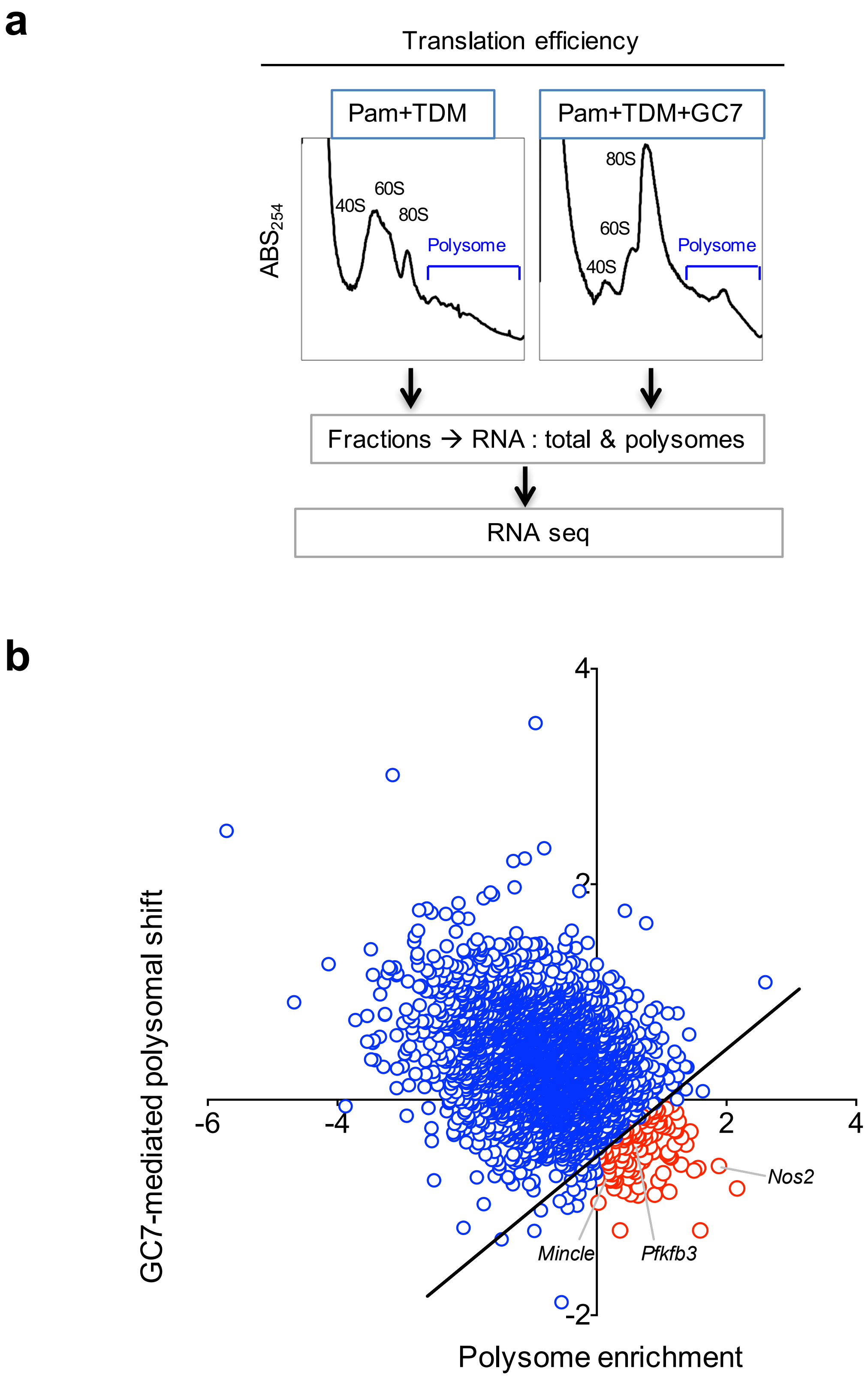

Supplementary Figure 17. Analysis of RNA-sequencing and polysome profiling (**a**) Work flow for the combined analysis of Mincle-eIF5A-dependent translation efficiency. (**b**) Screening results for wild-type BMDMs treated with Pam3, TDM or Pam3, TDM, GC7, presented as polysome enrichment (log ratio of polysomal RNA over total RNA in cells treated with Pam3 and TDM) and GC7-mediated polysomal shift (log ratio of polysome enrichment in cells treated with Pam3, TDM, GC7 over polysome enrichment in cells treated with Pam3, TDM). 111 genes (red dots) located in high score of polysome enrichment and low score of GC7-mediated polysomal shift were selected (polysome enrichment;x, GC7-mediated polysomal shift;y, x>0; y<0; x-2y>1).

Supplementary Figure 18

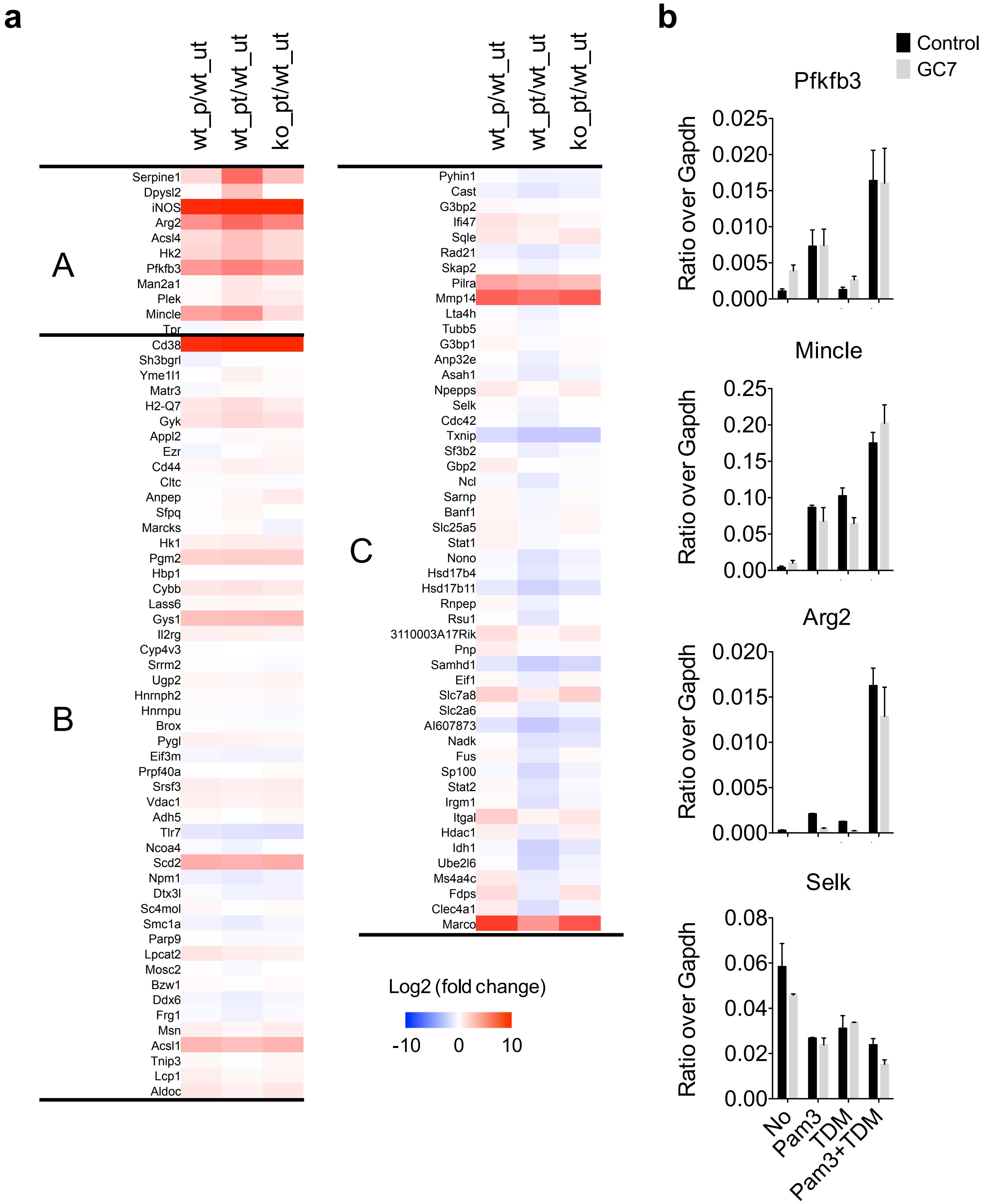

Supplementary Figure 18. Analysis of mRNA expression for selected genes from polysome profiling assay (**a**) Heatmap of RNAseq for selected 111 genes from polysome profiling assay of **Supplementary Figure 11b**. Wild-type (wt) and Mincle$^{-/-}$ (ko) BMDMs were untreated (ut) or stimulated with Pam3 (p) or co-stimulated with Pam3 and TDM (pt). Genes were classified into three groups from total RNAseq : Mincle-dependent induced genes (group A), no changed genes (group B), and Mincle-dependent reduced genes (group C). (**b**) qRT-PCR analysis of the indicated mRNA levels from each stimulated macrophages. WT BMDMs were stimulated with Pam3, TDM, or co-stimulated with Pam3 and TDM, in the presence of GC7 or control vehicle for 12 h.

Supplementary Figure 19

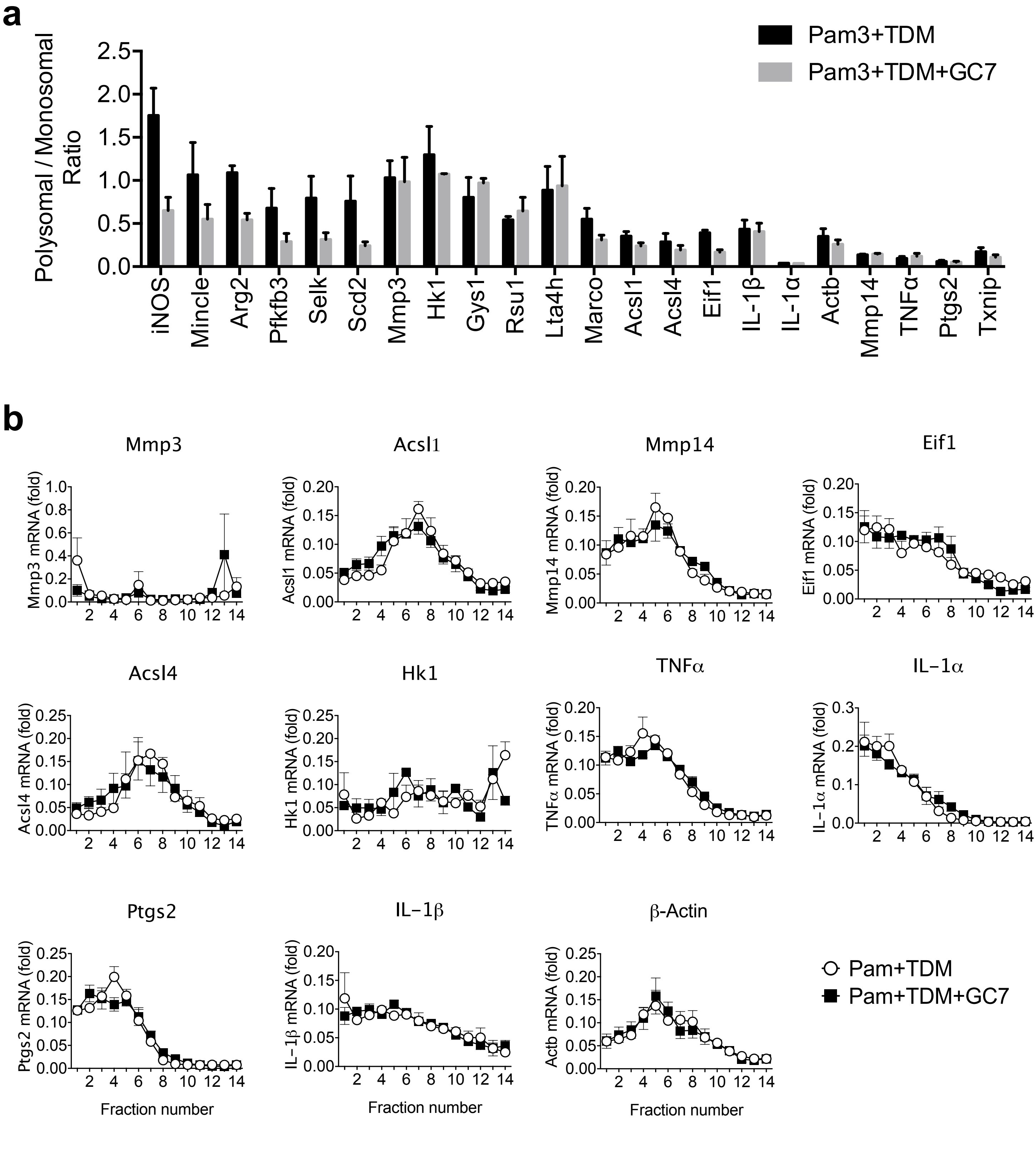

Supplementary Figure 19. Validation of polysome profiling assay by analyzing each fractions of the potential Mincle-eIF5A target mRNAs The polysome fraction mRNAs from WT BMDMs stimulated by Pam3 and TDM with or without GC7 were analyzed. (**a**) The translational efficiency of indicated mRNAs is represented by the ratio of mRNA content in polysomes (fraction 11-13) versus monosomes (fraction 4-6). (**b**) qRT-PCR analysis of indicated mRNAs in cells treated as indicated, presented for each fraction relative to the sum of all 14 polysome fractions.

Supplementary Figure 20

Figure 1a

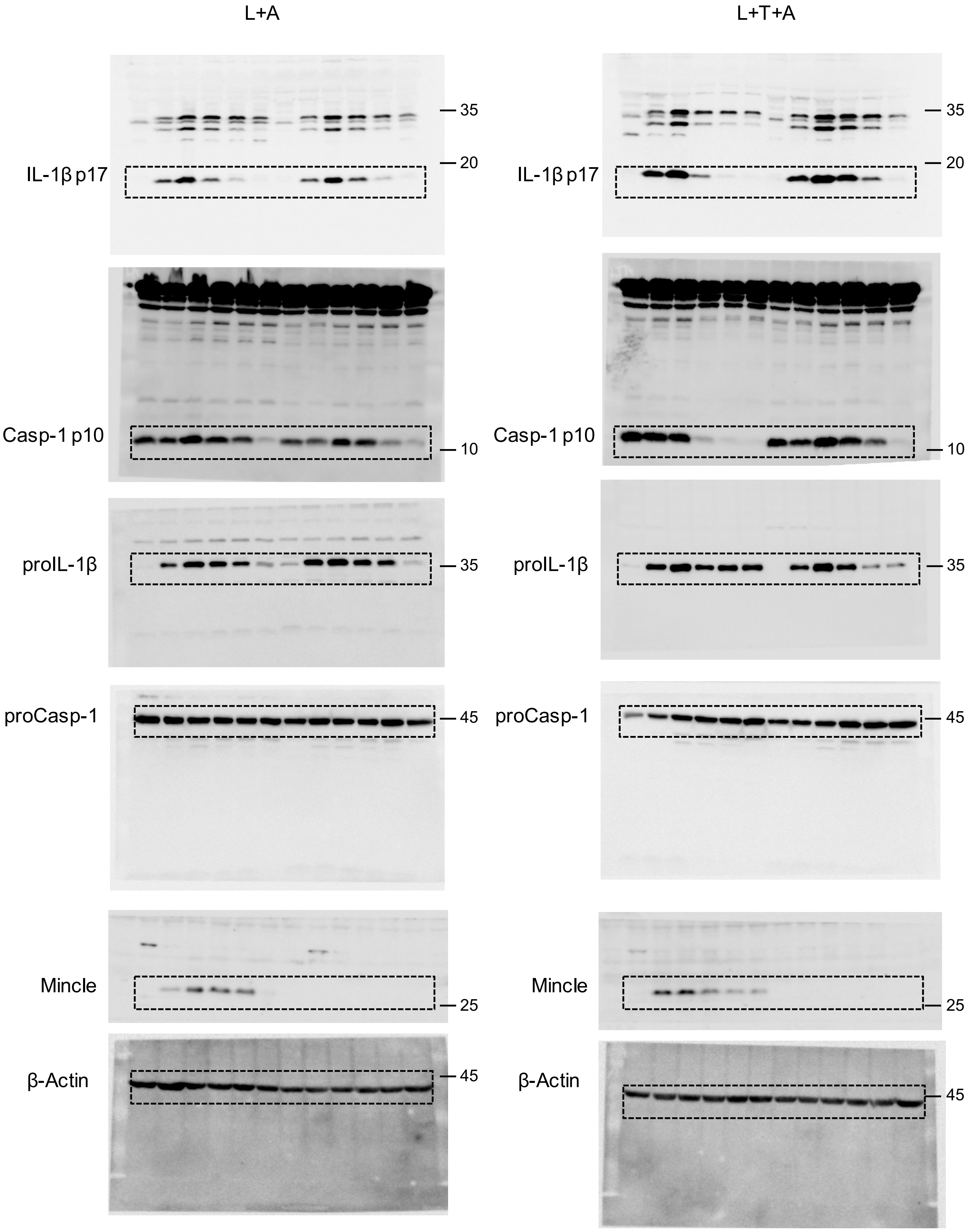

Figure 2b

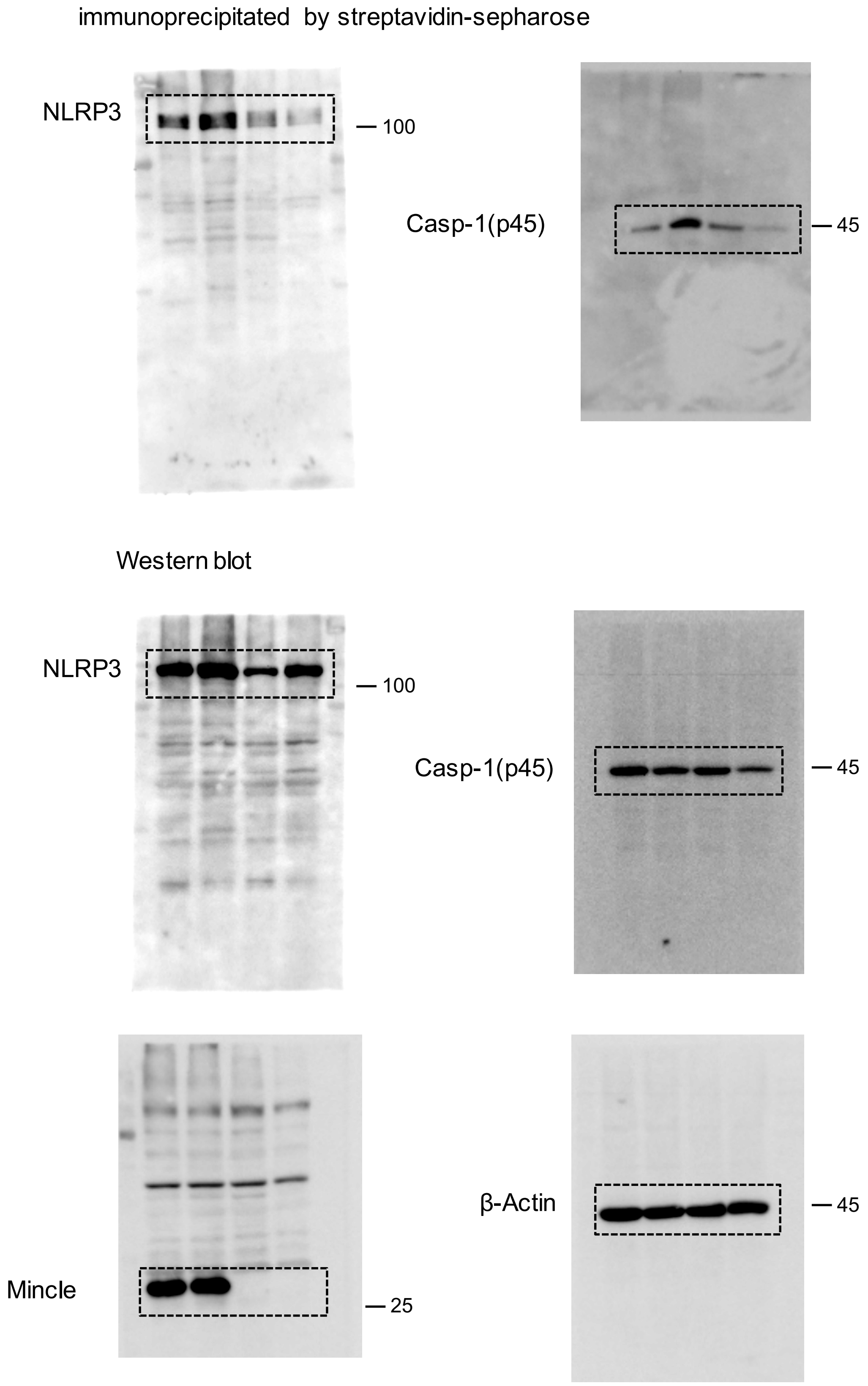
immunoprecipitated by streptavidin-sepharose
NLRP3
— 100
Casp-1(p45)
— 45
Western blot
NLRP3
— 100
Casp-1(p45)
— 45
Mincle
— 25
β-Actin
— 45

Figure 2c

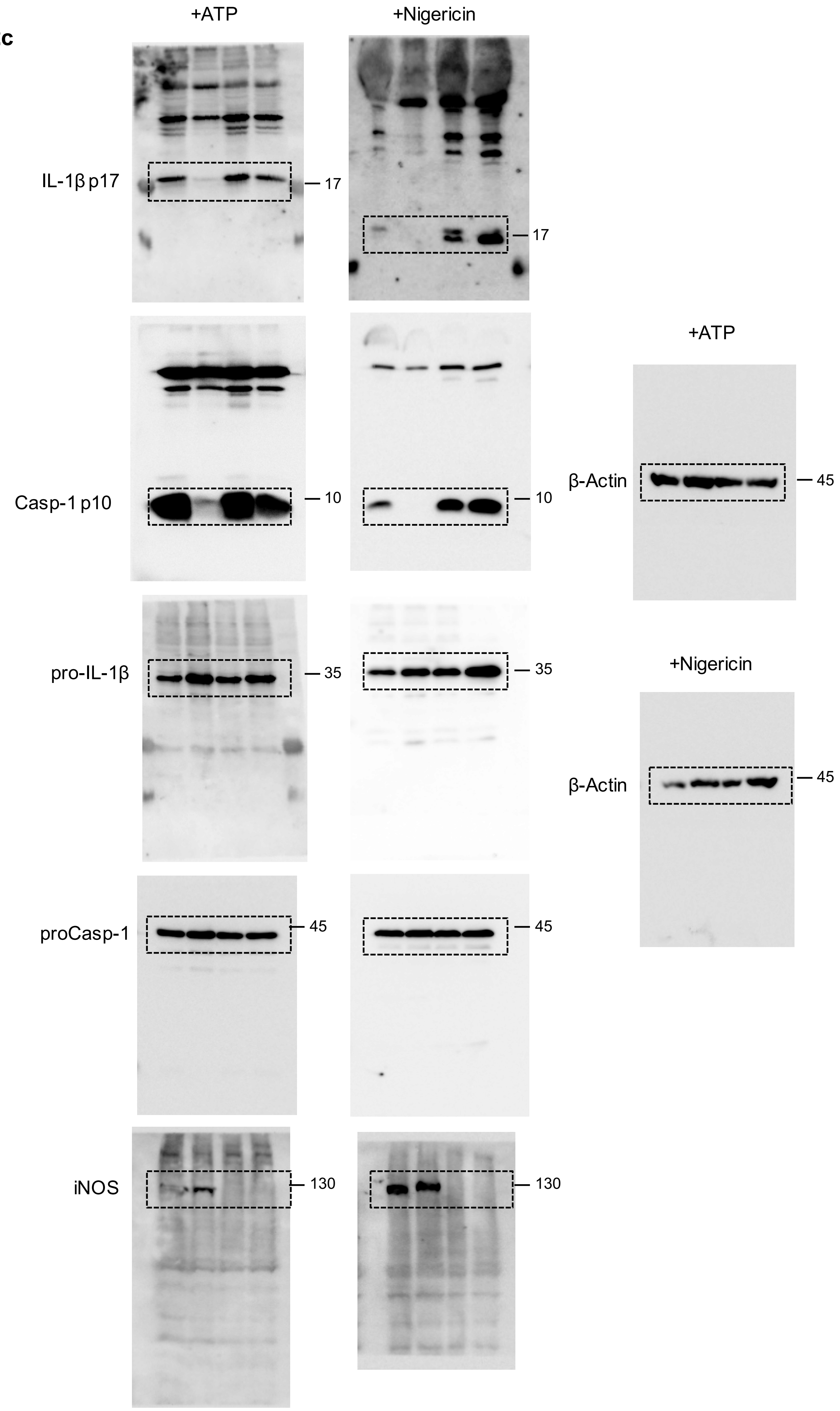

+ATP
+Nigericin
IL-1β p17
17
17
Casp-1 p10
10
10
pro-IL-1β
35
35
proCasp-1
45
45
iNOS
130
130
+ATP
β-Actin
45
+Nigericin
β-Actin
45

Figure 3a

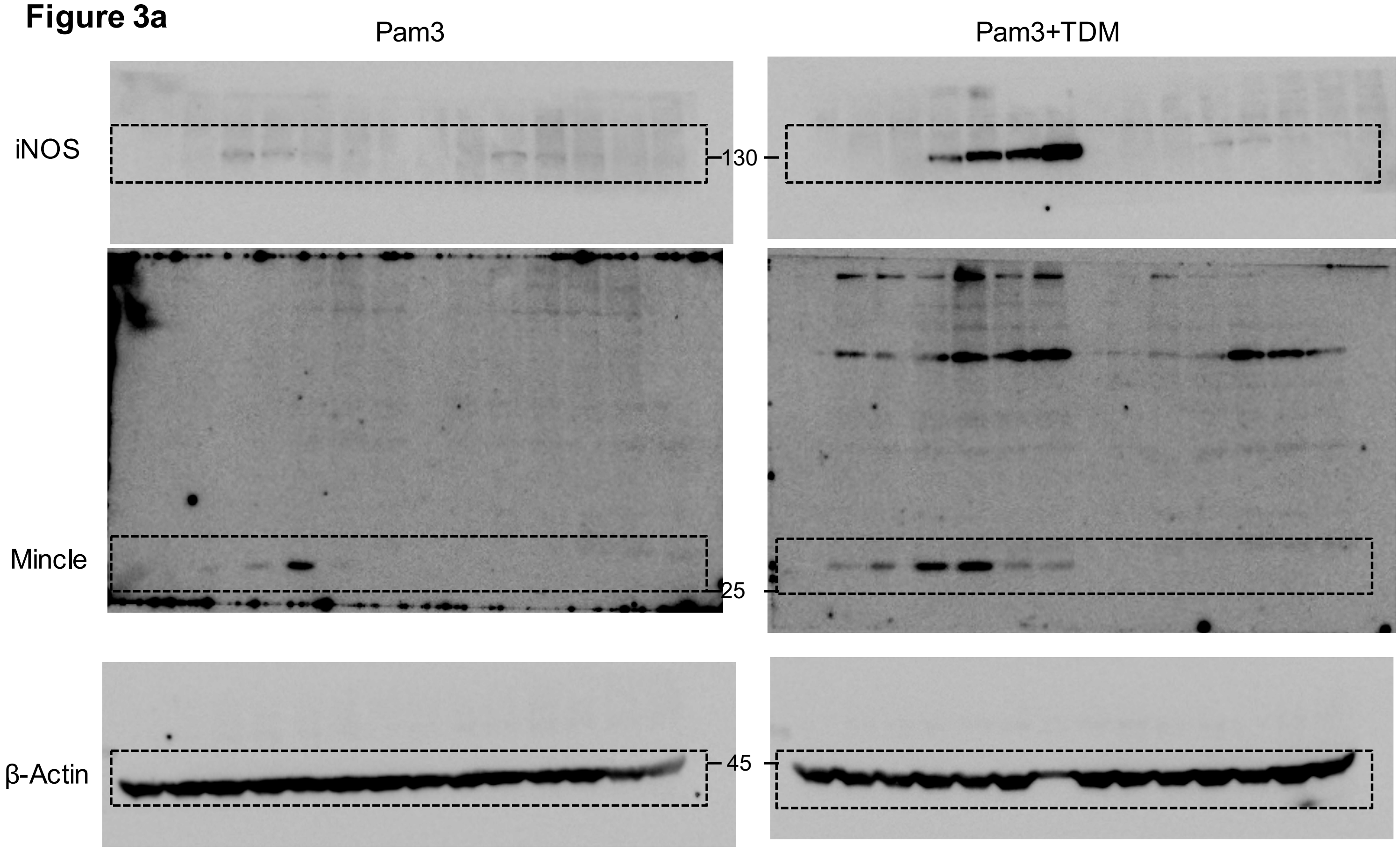

Figure 3d

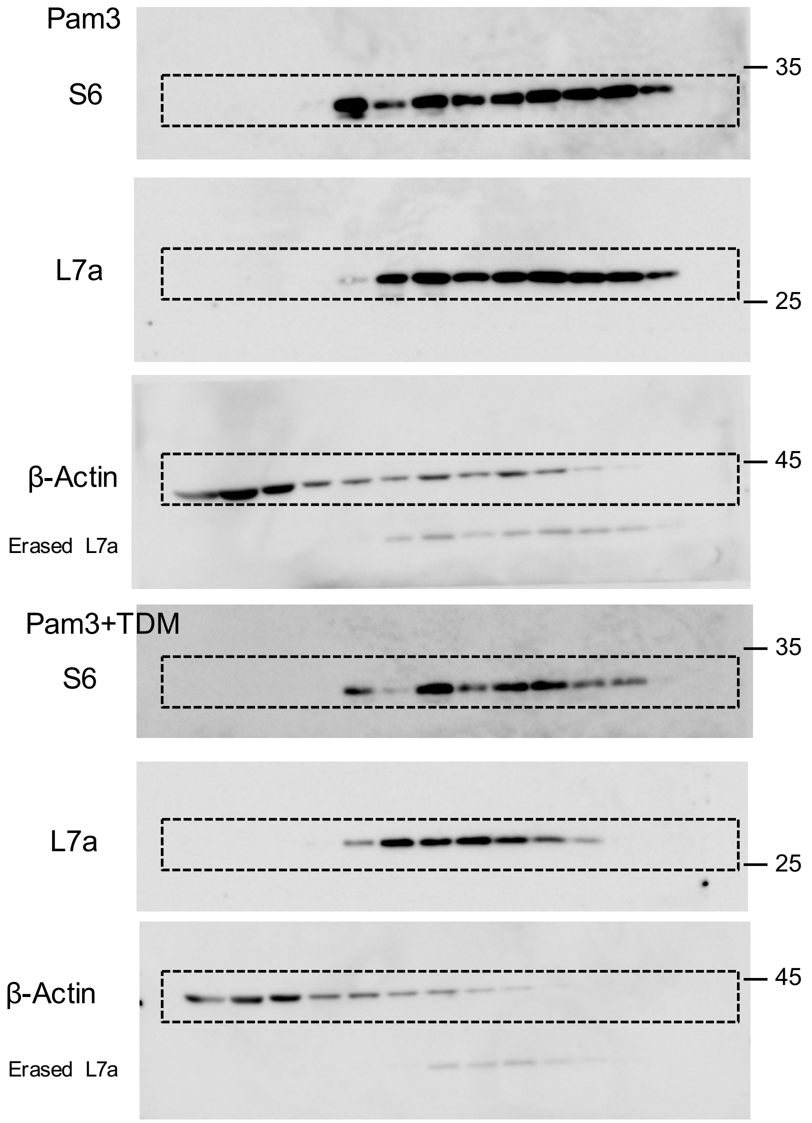

Figure 3c

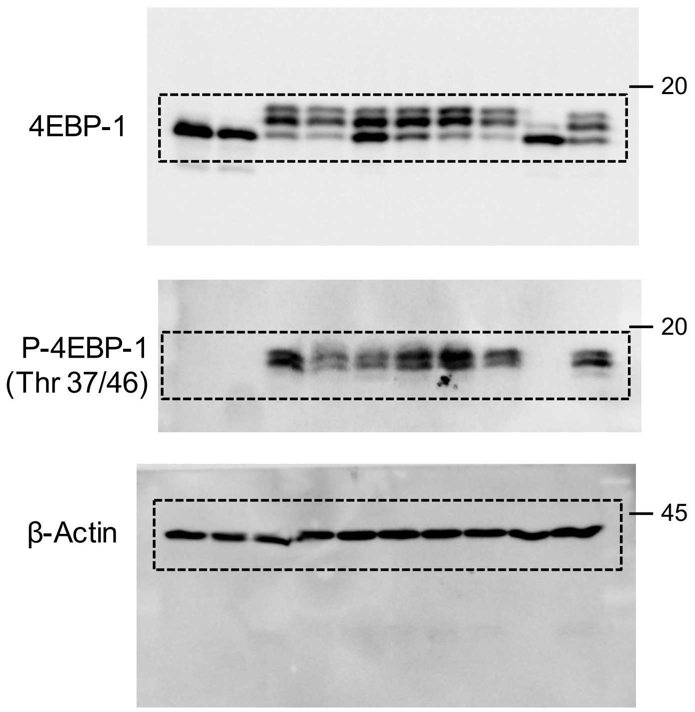

Figure 4b

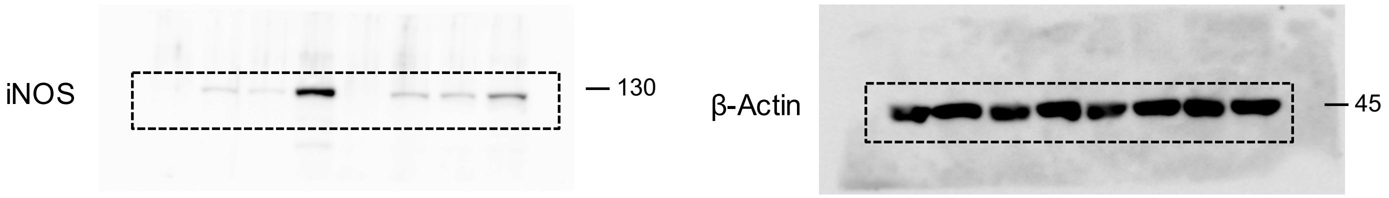

Figure 4c

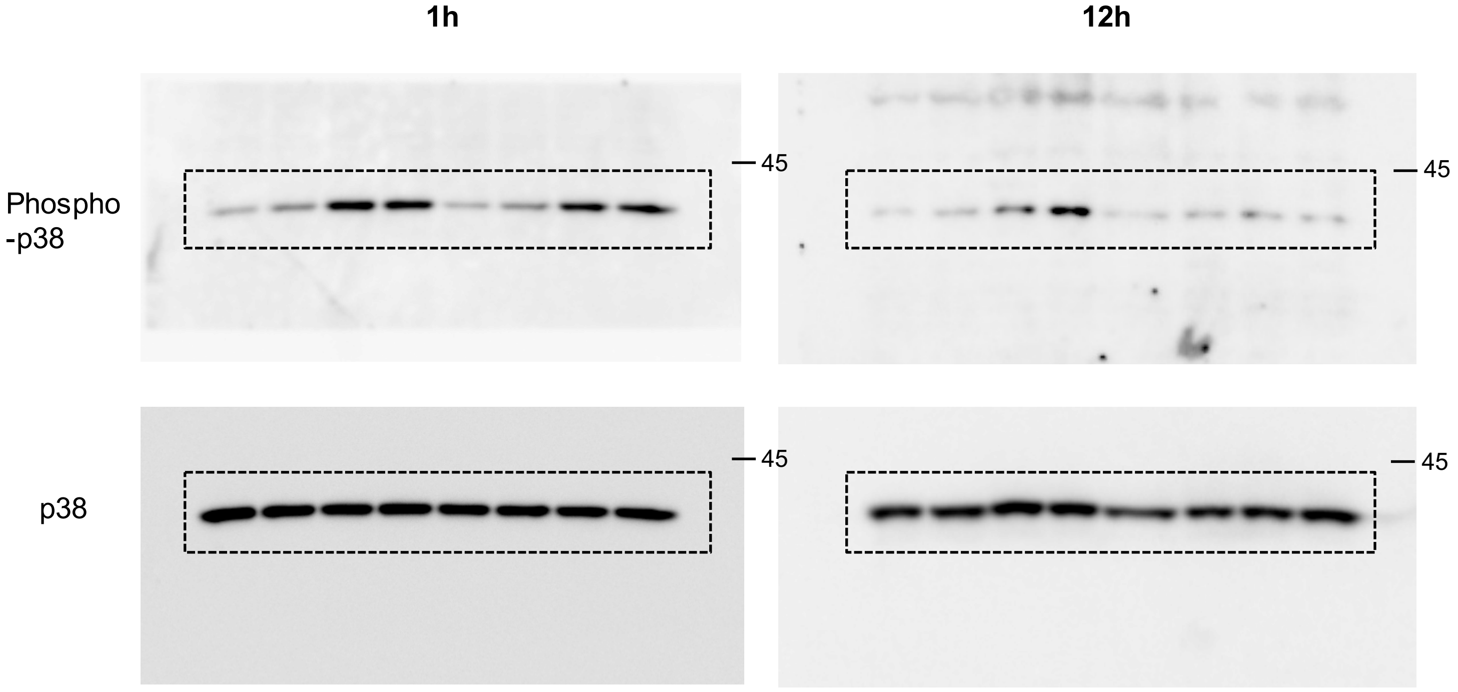

Figure 5b

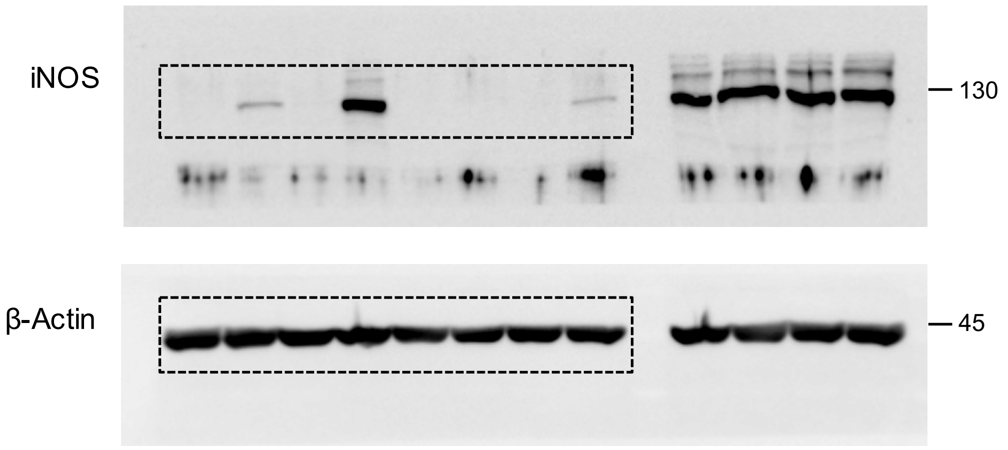

Figure 5c

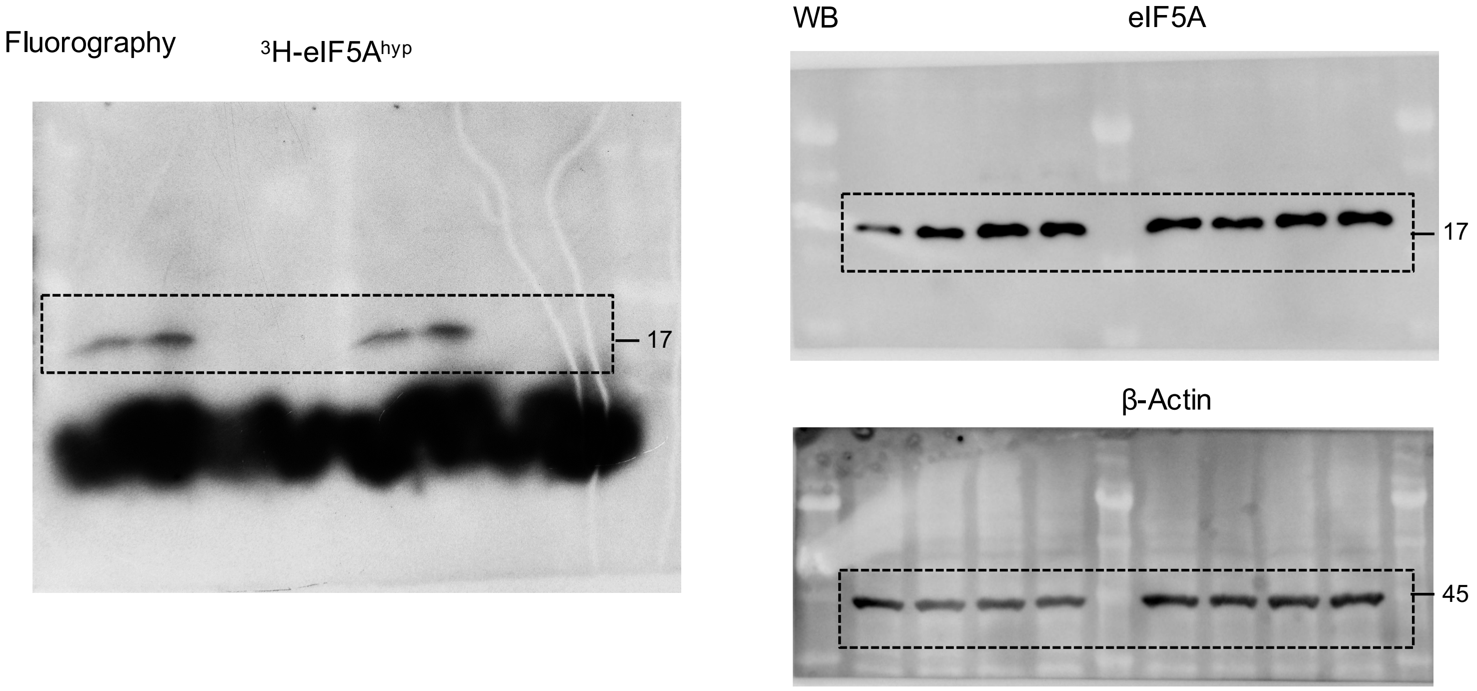

Figure 5d

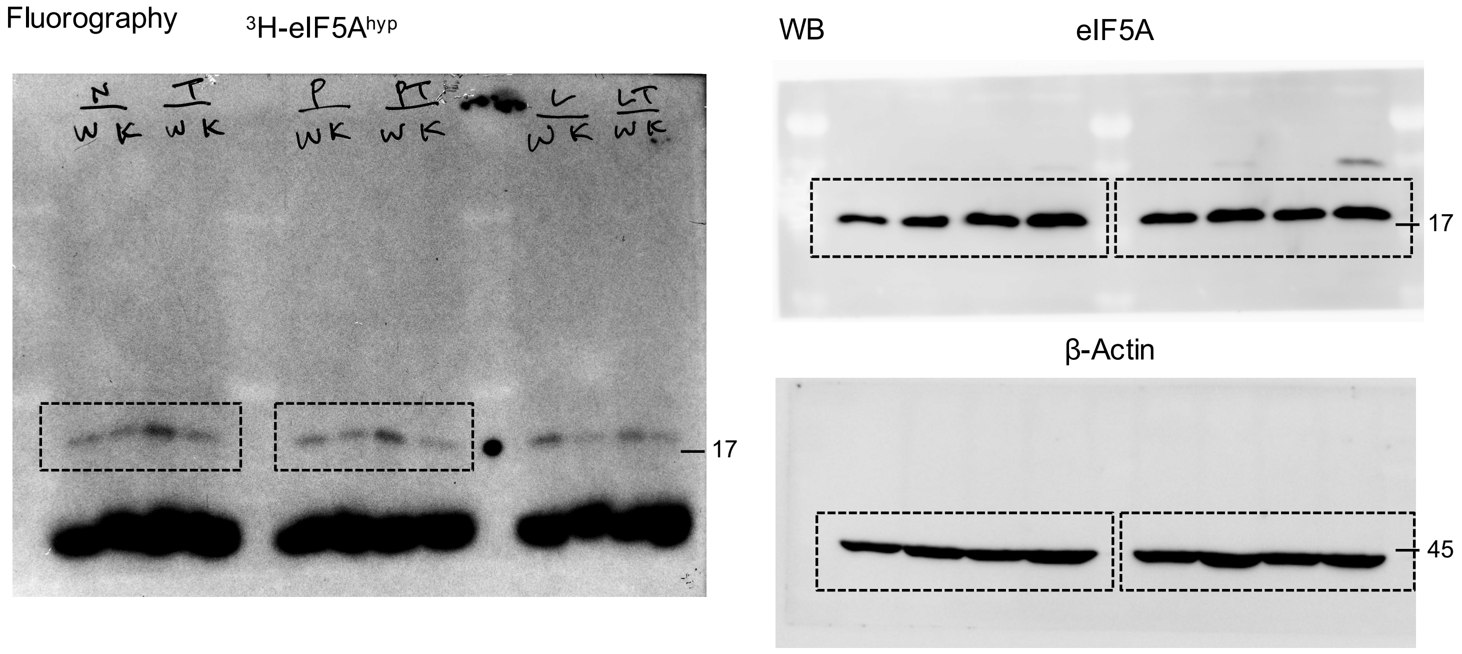

Figure 6c

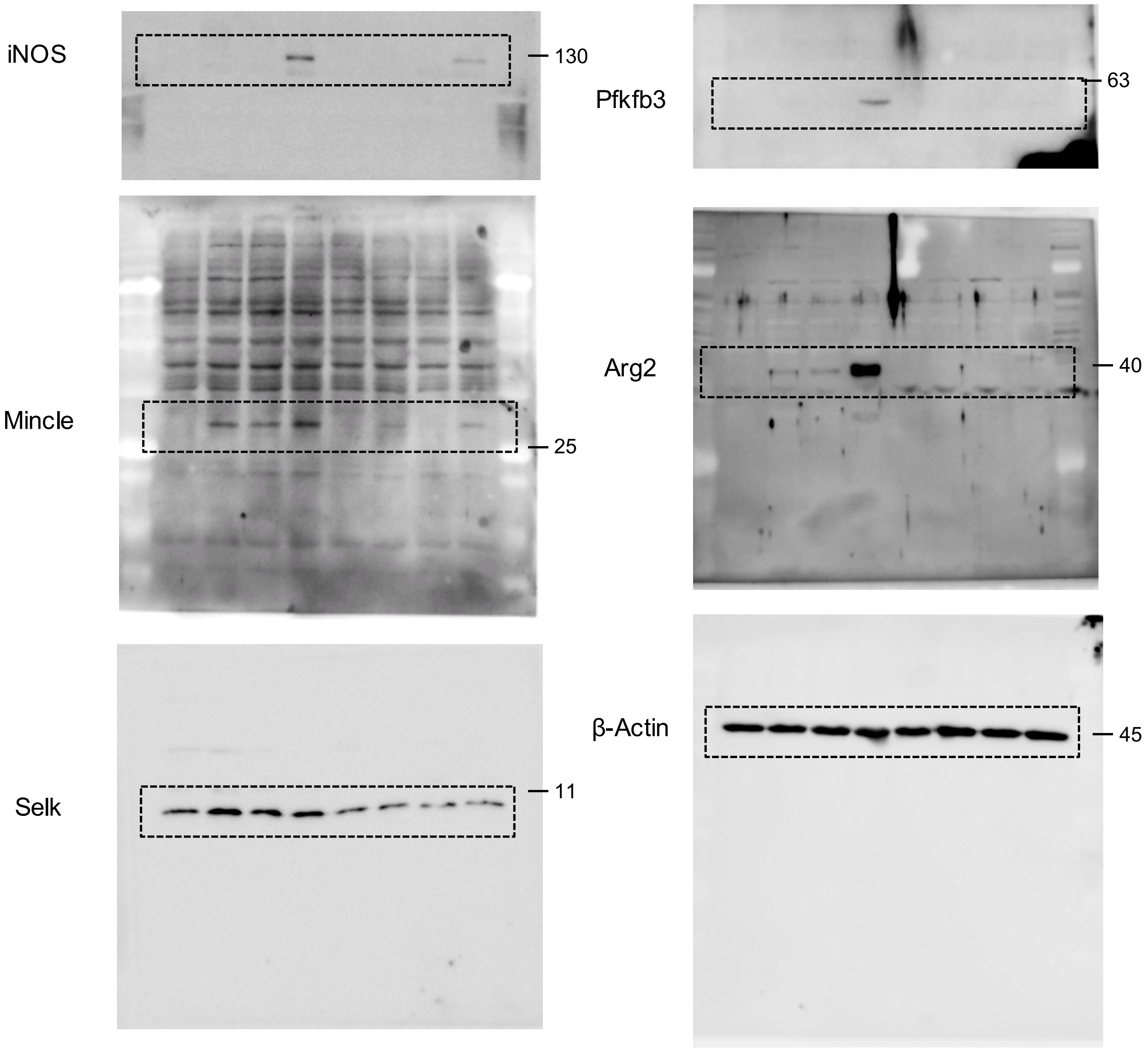

Figure 7e

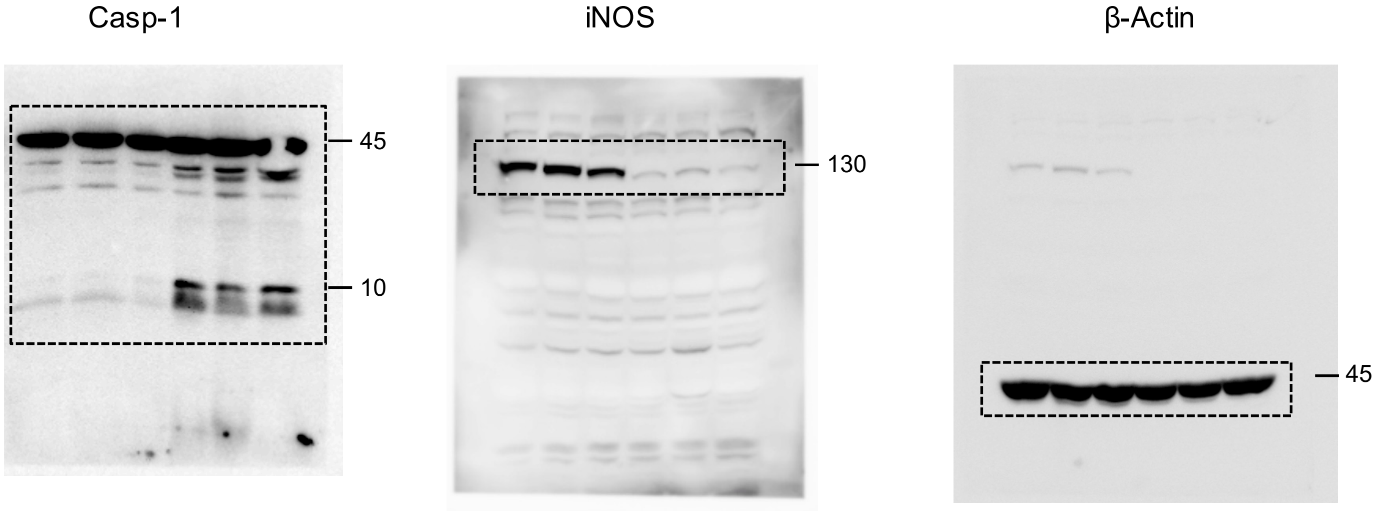

Figure 7i

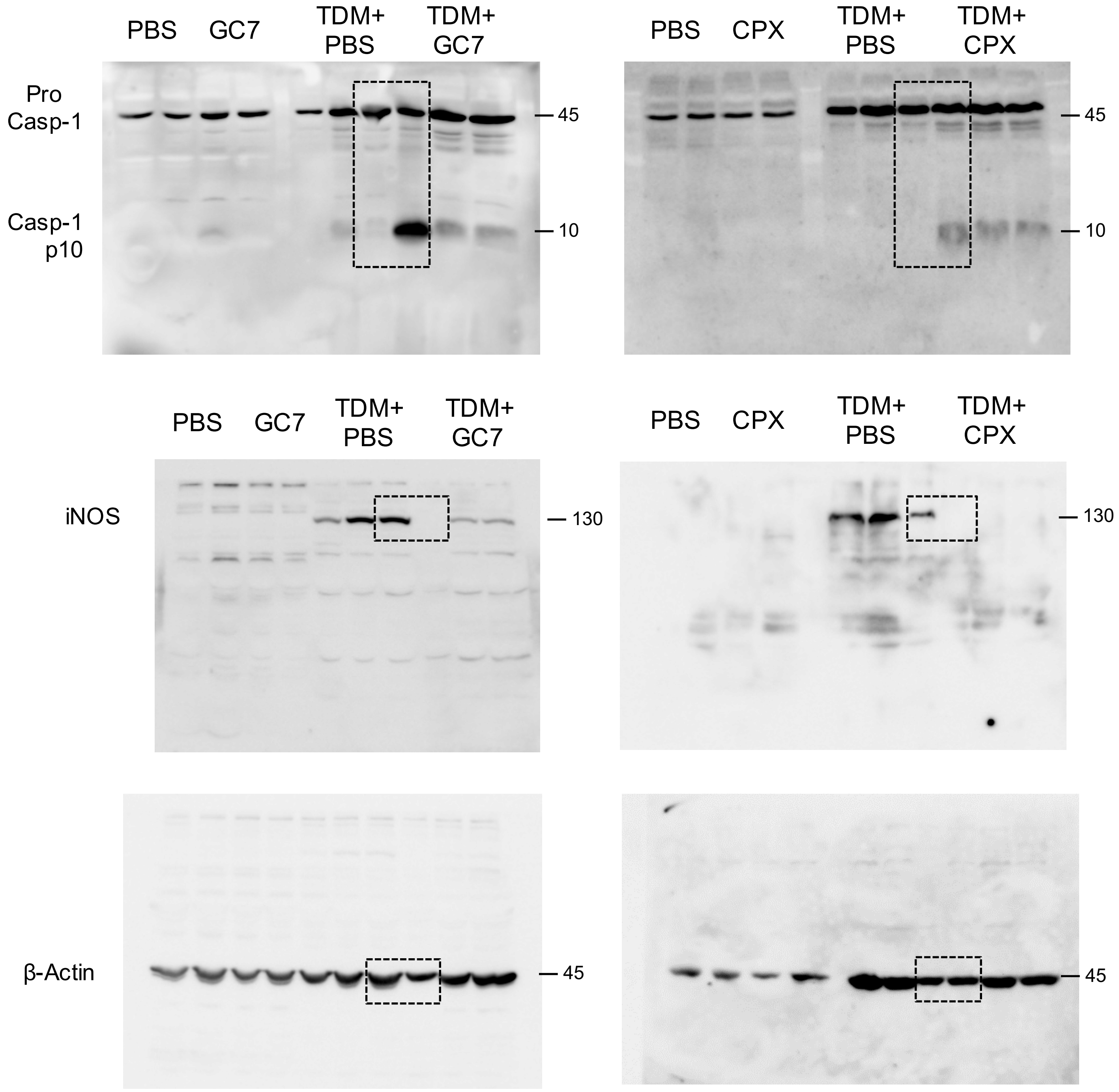

Supplementary Figure 3

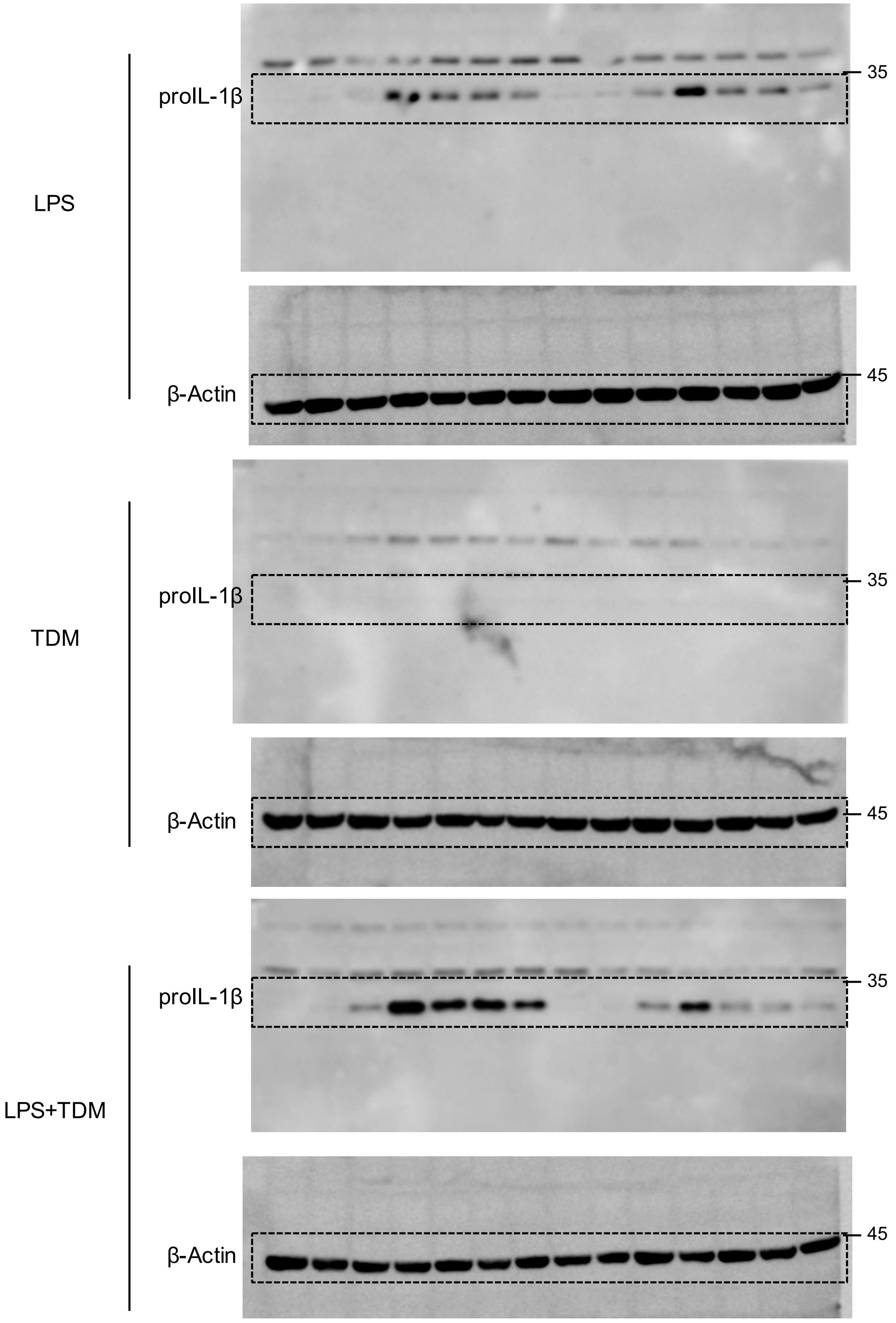

Supplementary Figure 4b

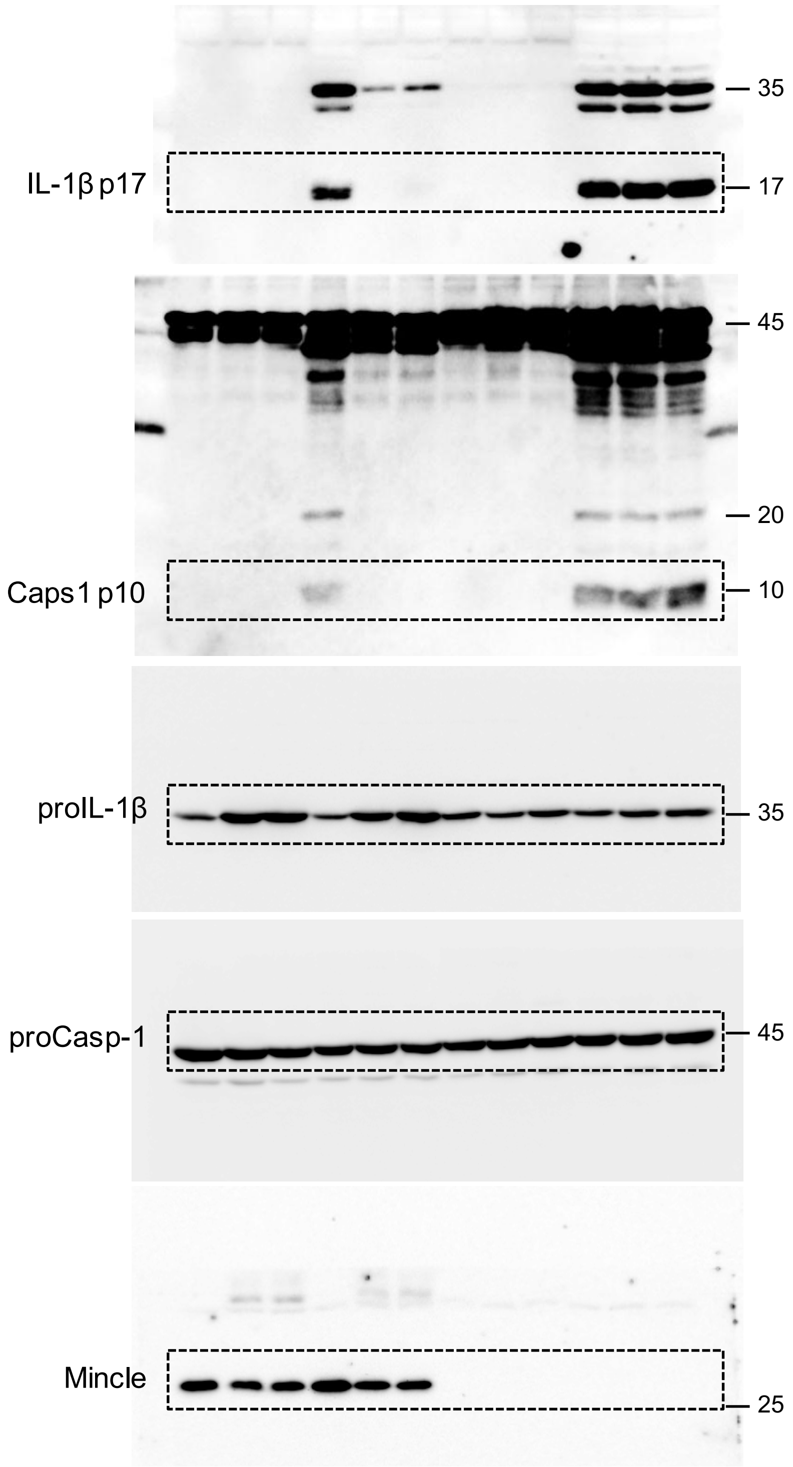

Supplementary Figure 5a

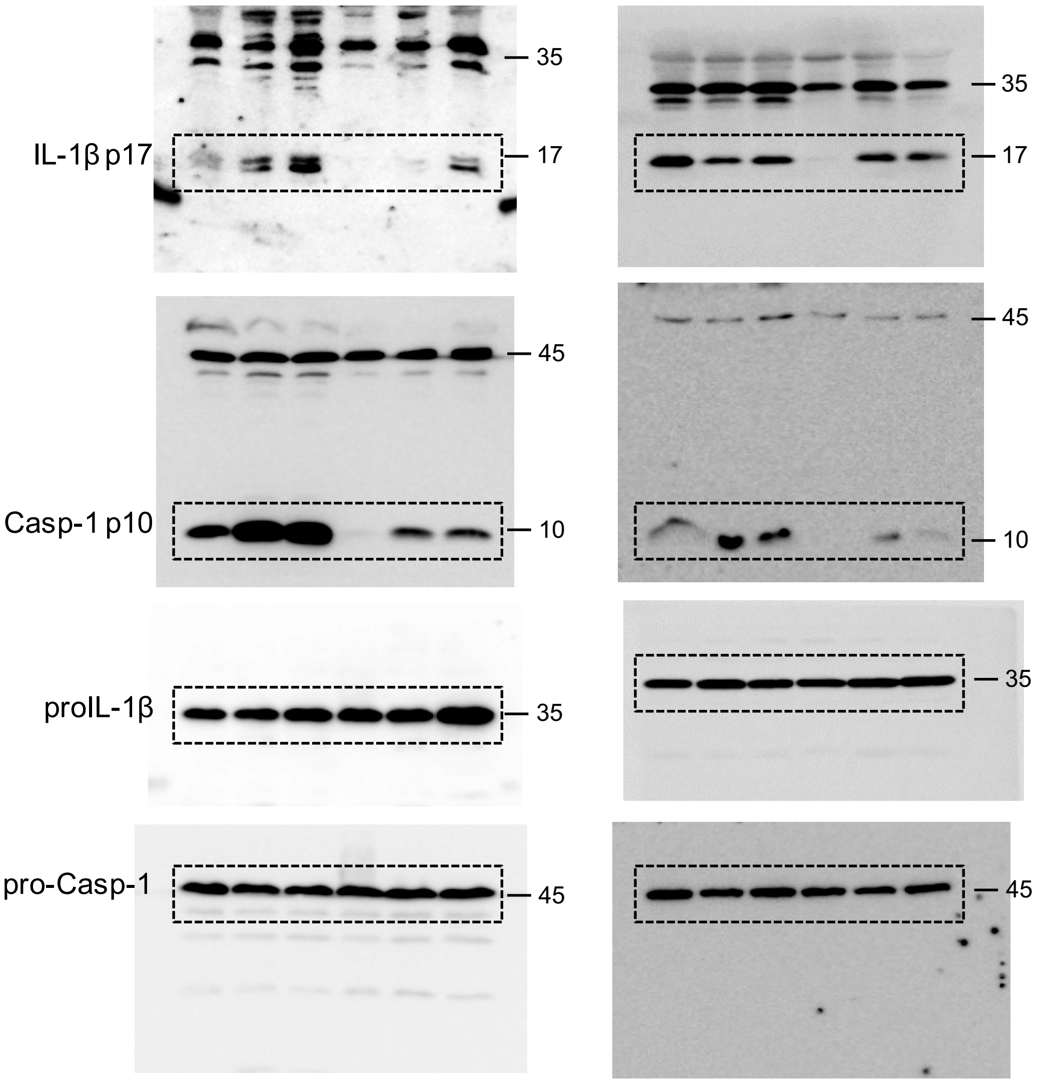

Supplementary Figure 6

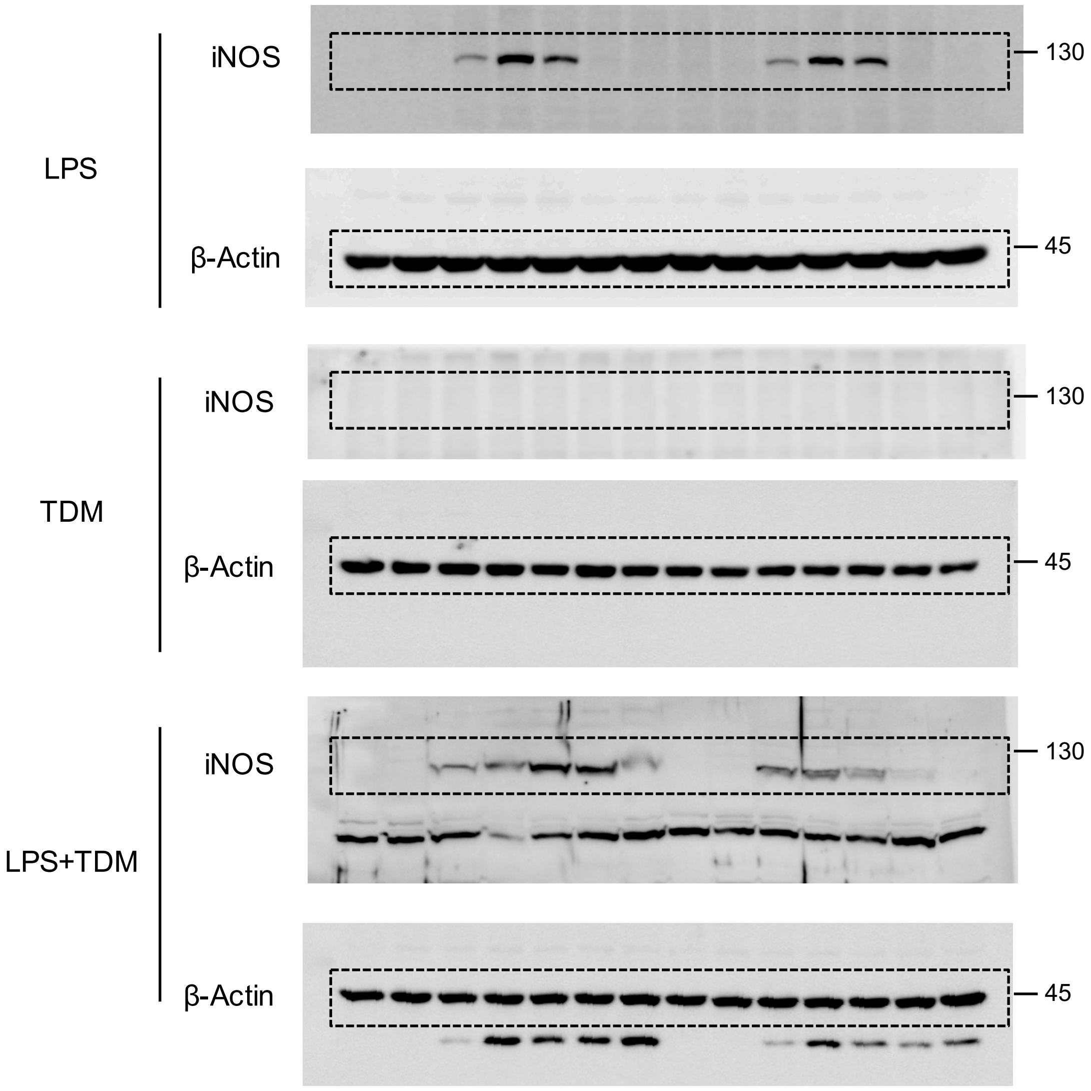

Supplementary Figure 7

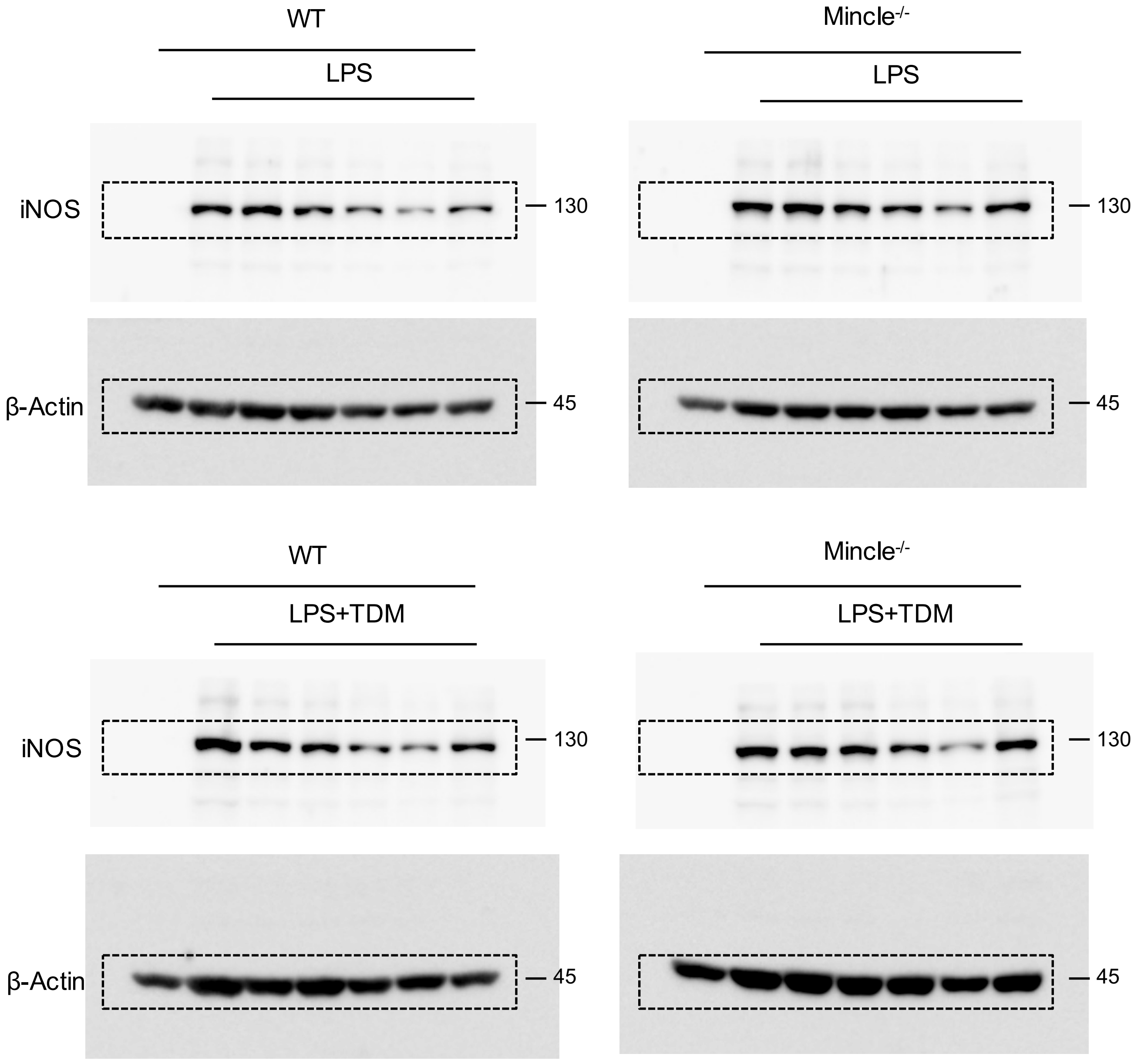

Supplementary Figure 8a

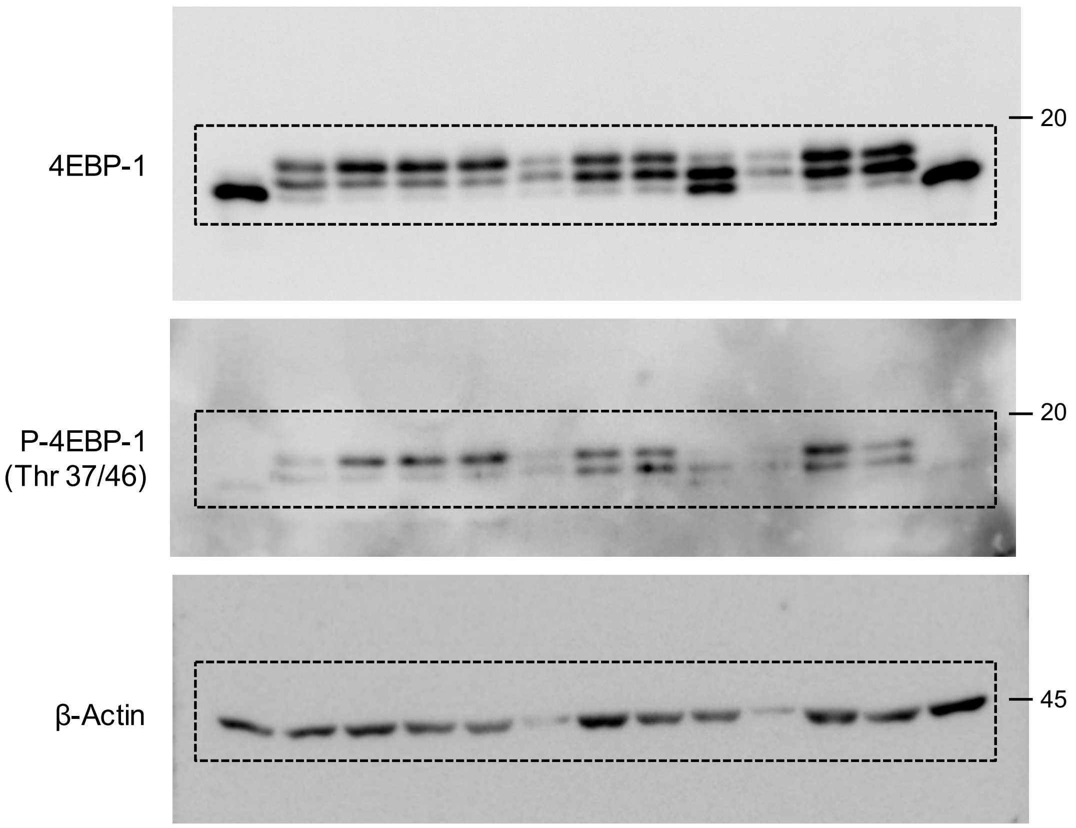

Supplementary Figure 8b

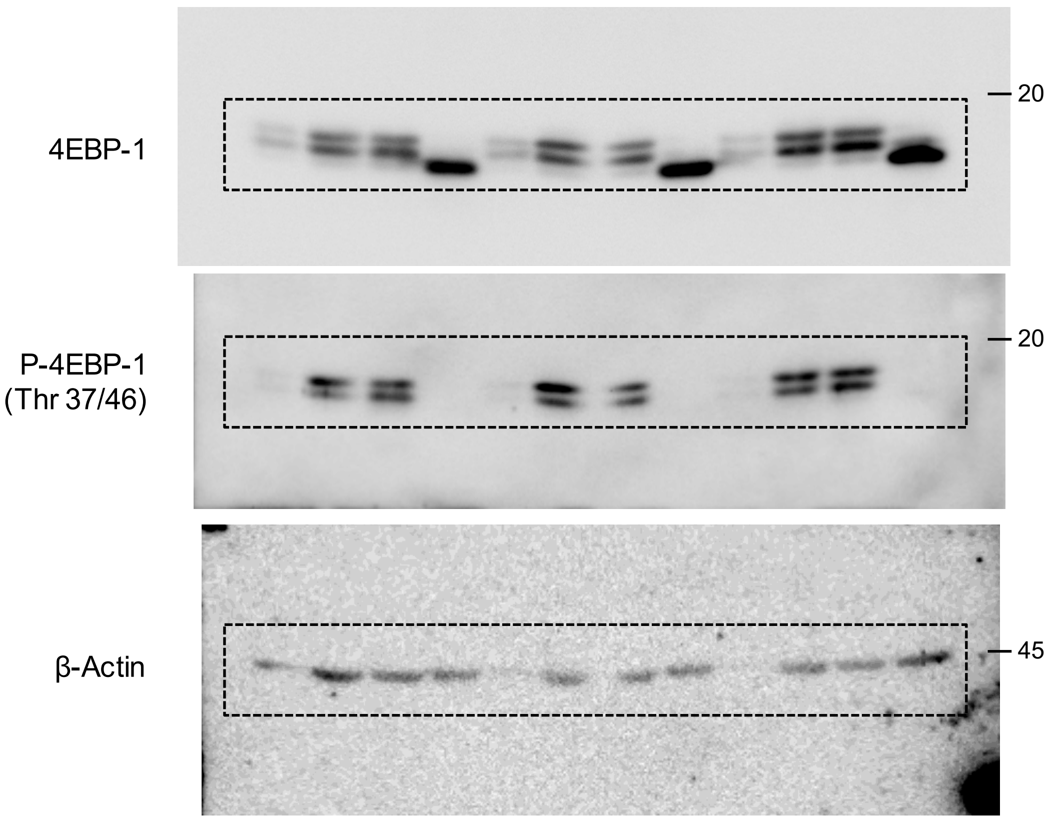

Supplementary Figure 11b

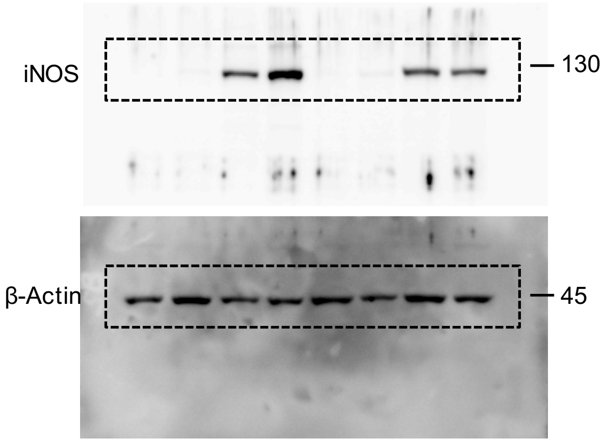

Supplementary Figure 12b

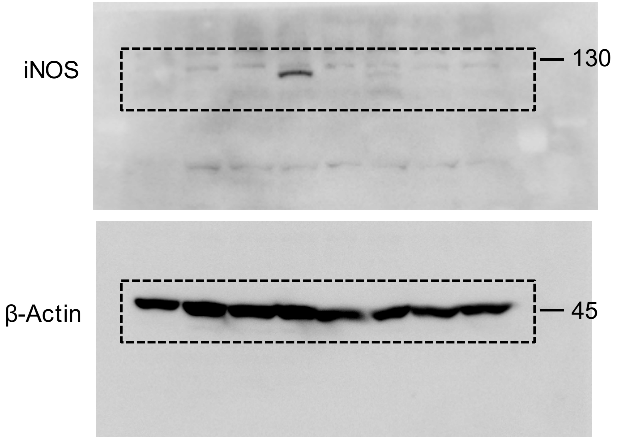

Supplementary Figure 13

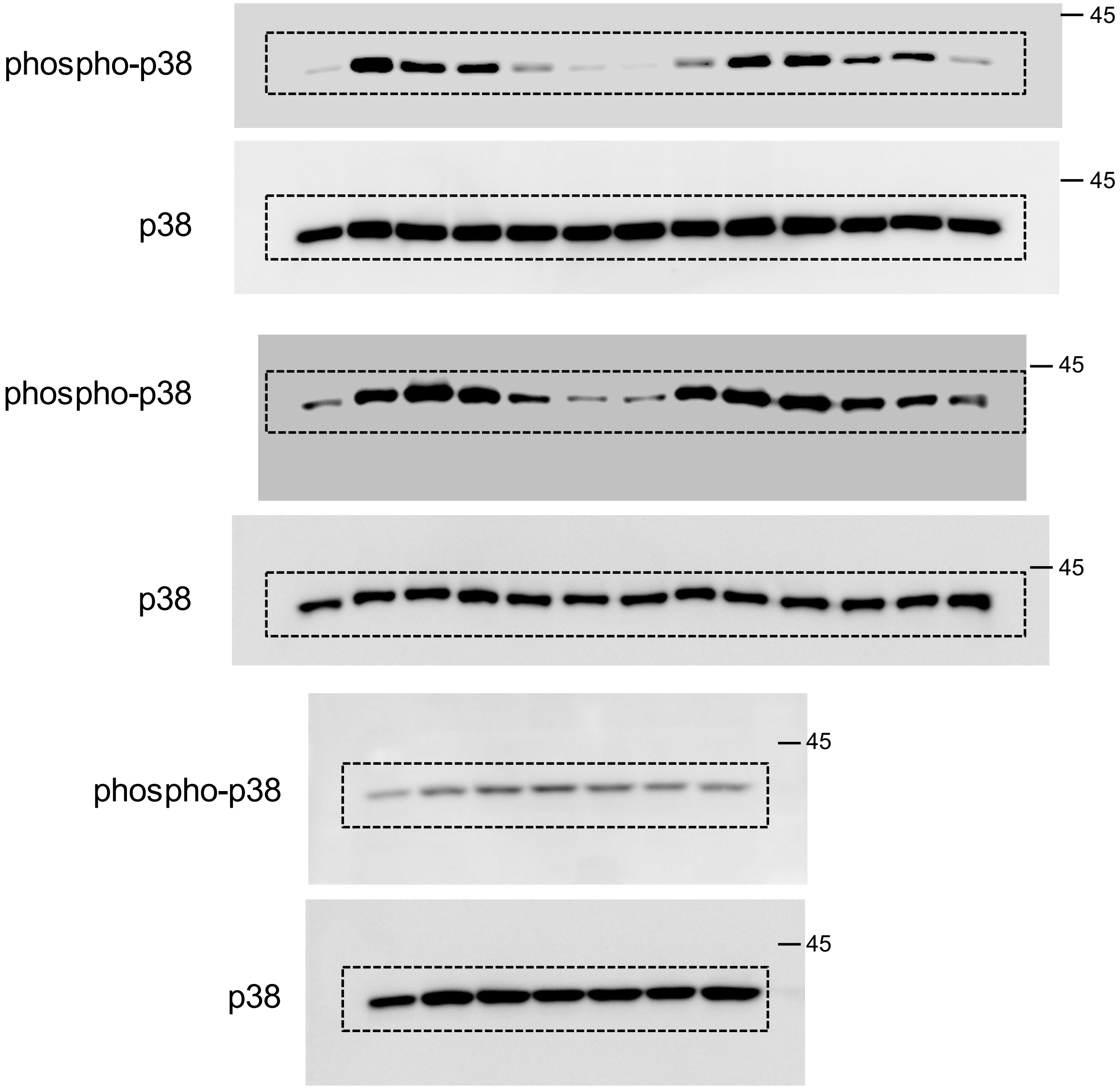

Supplementary Figure 14a

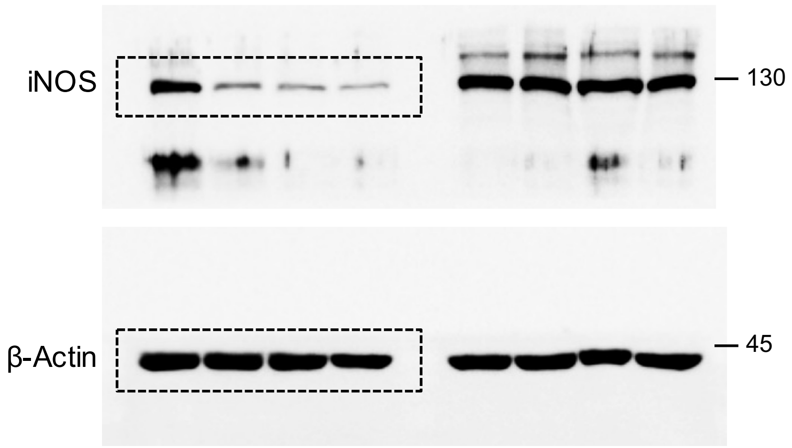

Supplementary Figure 14c

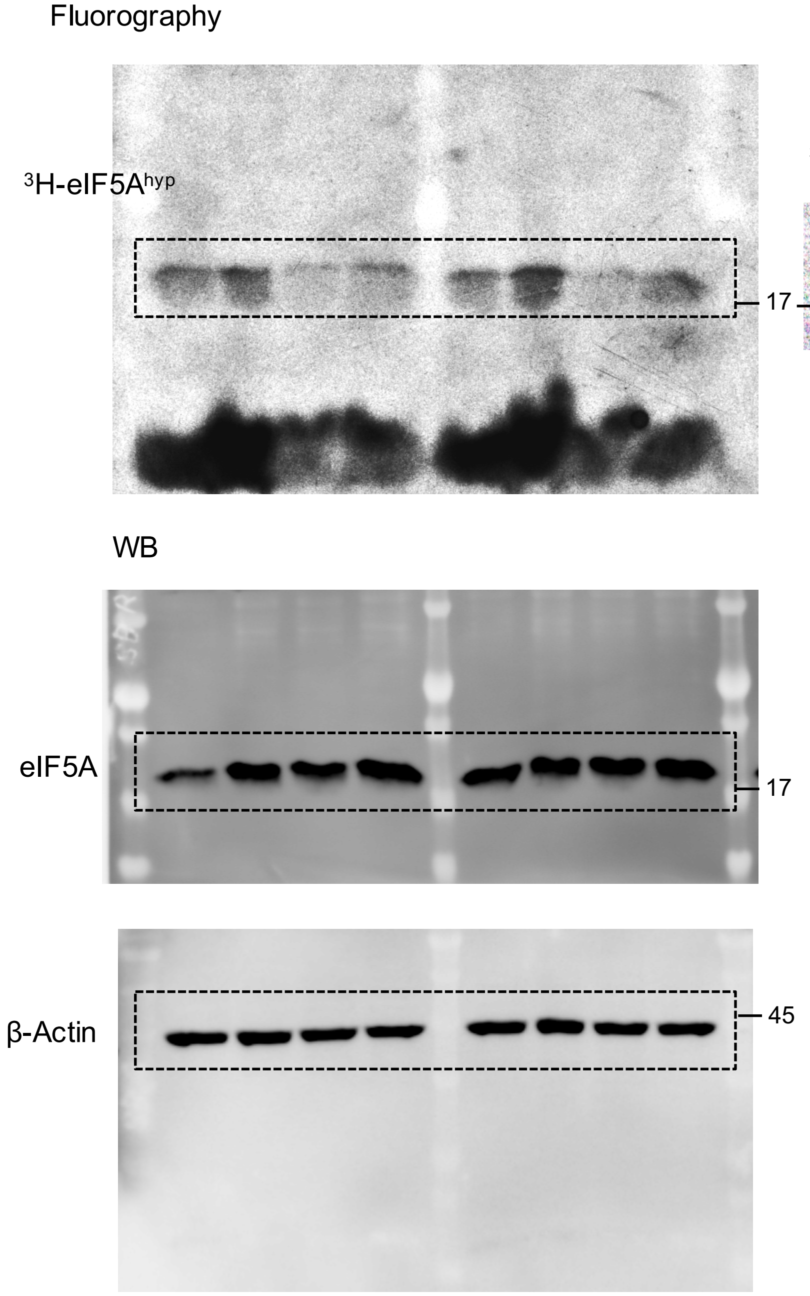

Supplementary Figure 14d

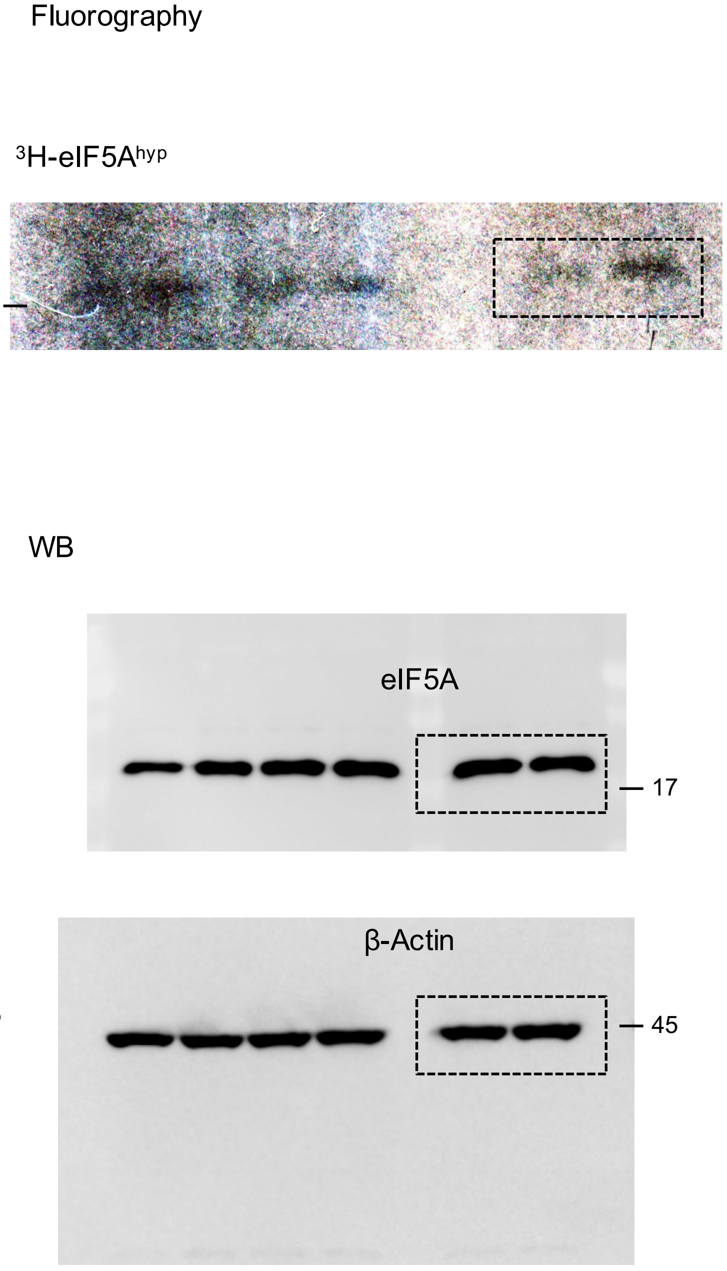

Supplementary Figure 15b

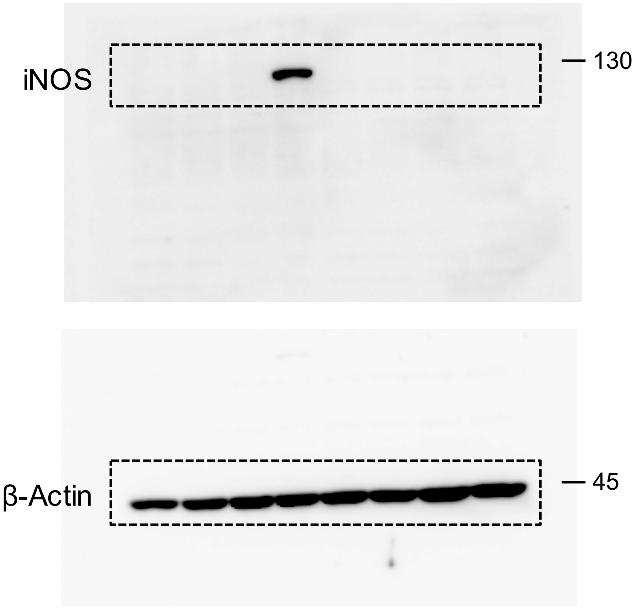

Supplementary Table 1
Gene ontology analysis from RNAseq.

3 fold increased by Pam3

GO Term ID	GO Term Name	Proportion	GO Term ORA P-Value
GO:0050729	positive regulation of inflammatory response	0.22	<1.0E-5
GO:0045087	innate immune response	0.08	<1.0E-5
GO:0006954	inflammatory response	0.13	<1.0E-5
GO:0030595	leukocyte chemotaxis	0.5	<1.0E-5
GO:0030593	neutrophil chemotaxis	0.27	<1.0E-5
GO:0009615	response to virus	0.11	<1.0E-5

3 fold decreased by Pam3

GO Term ID	GO Term Name	Proportion	GO Term ORA P-Value
GO:0007067	mitotic nuclear division	0.06	<1.0E-5
GO:0022409	positive regulation of cell-cell adhesion	0.27	0.00003

3 fold increased by Pam3 and TDM

GO Term ID	GO Term Name	Proportion	GO Term ORA P-Value
GO:0045087	innate immune response	0.11	<1.0E-5
GO:0006954	inflammatory response	0.15	<1.0E-5
GO:0042060	wound healing	0.13	0.00002
GO:0002544	chronic inflammatory response	0.44	0.00007
GO:0007263	nitric oxide mediated signal transduction	0.31	0.00036
GO:0001516	prostaglandin biosynthetic process	0.25	0.00086

3 fold decreased by Pam3 and TDM

GO Term ID	GO Term Name	Proportion	GO Term ORA P-Value
GO:0035457	cellular response to interferon-alpha	0.56	<1.0E-5
GO:0009615	response to virus	0.1	0.00099
GO:0051607	defense response to virus	0.08	0.00187

Supplementary Table 2
Antibody information

Antibody	Clone	Distributor
anti-β-Actin	#4967	Cell signaling Technology
anti-iNOS	#2982	Cell signaling Technology
anti-S6 (5G10)	#2217	Cell signaling Technology
anti-L7a (E109)	#2415	Cell signaling Technology
anti-Phospho-p38 (D3F9)	#4511	Cell signaling Technology
anti-Pfkfb3	#13123	Cell signaling Technology
anti-Eif1	#12496	Cell signaling Technology
anti-4E-BP1	#9644	Cell signaling Technology
anti-Phospho-4E-BP1 (Thr37/46)	#9459	Cell signaling Technology
anti-Phospho-4E-BP1 (Ser65)	#9451	Cell signaling Technology
anti-Il-1β	5129-100	Biovision
anti-Caspase-1 p10 (M-20)	sc-514	Santa Cruz Biotechnology
anti-p38 (C-20)	sc-535	Santa Cruz Biotechnology
anti-Cox-2 (C-20)	sc-1745	Santa Cruz Biotechnology
anti-eIF5A (EP526Y)	ab32443	Abcam
anti-NLRP3 (Cryo-2)	AG-20B-0014	Adipogen
anti-Mincle	D292-3	MBL international
anti-Stat1	#9172	Cell signaling Technology
anti-Stat2	#07-140	Merk Millipore
anti-Fdps	PA5-28228	Thermo Scientific
anti-Txnip	K0204-3	MBL international
anti-Mmp3	#14351	Cell signaling Technology
anti-Arg2	sc-20151	Santa Cruz Biotechnology
anti-Selk	PA5-34420	Thermo Scientific

Supplementary Table 3
Primers for real-time PCR

Gene	GeneBank_ID	5'Forward sequence3'	3'Reverse sequence5'
Gapdh	NM_008084	GGCAAATTCAACGGCACAGTCAAG	TCGCTCCTGGAAGATGGTGATGG
iNOS	NM_010927	AATCTTGGAGCGAGTTGTGG	CAGGAAGTAGGTGAGGGCTTG
Actb	NM_007393	AGATGTGGATCAGCAAGCAGGAG	GCTCAGTAACAGTCCGCCTAGAAG
Pfkfb3	NM_001177753	GTCGCCGAATACAGCTACGA	GAGCCCCACCATCACAATCA
Mincle	NM_019948	AGTGAGGCATCAGGTTCAGTCAAG	GACCAGGTCAAGGTTGTCGTAGAG
TNFa	NM_013693	ATGTCCATTCCTGAGTTCTG	AATCTGGAAAGGTCTGAAGG
MIP-2	NM_009140	CATCCCACCCACACAGTGAAAGAG	CCTTCCATGAAAGCCATCCGACTG
Selk	NM_019979	CGGTCTTCTCTGTCGCTAGG	TTCCTGCTGTCCAACACCTG
Scd2	NM_009128	AGATGATCTATATGACCCCACCT	CCCAGGGCGCTGATTACATA
Eif1	NM_011508	ACAGGTTGAGCAGTAAGCCC	GACATAGCTGGGGCAGTGTT
Mmp3	NM_010809	CTCATGCCTATGCACCTGGA	GGCTGAGTGGTAGAGTCCCA
Acsl1	NM_007981	CGGCCGCGACTCCTTAAAT	TCCATGGTTCTGGGTTGGTG
Acsl4	NM_019477	GCTCCAGAGCTAGCGGG	TGTCTGAAGTGGGCTTAGCTT
Mmp14	NM_008608	CGCGCTCTAGGAATCCACAT	TTCTCATGTCCCTCCCGGAT
Hk1	NM_010438	GGATGGGAACTCTCCCCTGA	GCATACGTGCTGGACCGATA
IL-1a	NM_010554	AGGGAGTCAACTCATTGGCG	CTTCCCGTTGCTTGACGTTG
IL-1b	NM_008361	TCAACCAACAAGTGATATTCTC	ACACAGGACAGGTATAGATTC
Ptgs2	NM_011198	GCCCGTGCTGCTCTGTCTTAAC	GTTGCTCTAGGCTTTGCTGGCTAC
Rps28	NM_016844	CAGCCCATCAAGCTGGCTA	CCAGAACCCAGCTGCAAGAT
